# Baicalin Alleviates Piglet Immunosuppression Induced by *Glaesserella parasuis* via Promoting CD163/Tumor Necrosis Factor-like Weak Inducer of Apoptosis-Mediated Autophagy

**DOI:** 10.3390/biom15050722

**Published:** 2025-05-15

**Authors:** Shulin Fu, Ronghui Luo, Jingyang Li, Yunjian Fu, Qiaoli Dong, Siyu Liu, Yamin Sun, Ling Guo, Jin Hu, Yinsheng Qiu

**Affiliations:** 1 Wuhan Engineering and Technology Research Center of Animal Disease-Resistant Nutrition, School of Animal Science and Nutritional Engineering, Wuhan Polytechnic University, Wuhan 430023, China; 20230411035@whpu.edu.cn (R.L.); 20220411050@whpu.edu.cn (J.L.); 20230411033@whpu.edu.cn (Y.F.); 20230411045@whpu.edu.cn (Q.D.); 20220411030@whpu.edu.cn (S.L.); 20230412014@whpu.edu.cn (Y.S.); guoling1101@whpu.edu.cn (L.G.); hujin@whpu.edu.cn (J.H.); qiuyinsheng6405@whpu.edu.cn (Y.Q.); 2 Hubei Key Laboratory of Animal Nutrition and Feed Science, School of Animal Science and Nutritional Engineering, Wuhan Polytechnic University, Wuhan 430023, China

**Keywords:** baicalin, *Glaesserella parasuis*, immunosuppression, CD163/TWEAK, autophagy

## Abstract

*Glaesserella parasuis* (*G. parasuis*) causes vascular inflammation in piglets, resulting in vascular damage. However, the mechanism causing vascular inflammation remains unclear. Baicalin possesses an anti-inflammatory function. Tumor necrosis factor-like weak inducer of apoptosis (TWEAK) has been implicated in immunosuppression. CD163, a scavenger receptor expressed on macrophages that acts as a decoy receptor for TWEAK, plays a crucial role in the regulation of autophagy and inflammation. This research investigated the efficacy of baicalin in reducing immunosuppression elicited by *G. parasuis* through the regulation of CD163/TWEAK-mediated autophagy. The data demonstrated that *G. parasuis* altered routine blood indicators and biochemical parameters, increased cytokine production, and induced blood vessel tissue damage. *G. parasuis* reduced the CD3+ T cell proportion, CD3+CD4+ T cell proportion, and CD3+CD8+ T cell proportion in piglet blood. The proteomic analysis revealed that CD163 was differentially expressed in the blood vessels of challenged piglets. Baicalin was found to regulate CD163/TWEAK axis expression, inhibit Notch/Wnt signaling pathway activation, promote autophagy, and reduce NLRP3/Caspase 1 signaling pathway activation. Baicalin also decreased cytokine production and alleviated pathological tissue damage in the blood vessels of *G. parasuis*-challenged piglets. Taken together, this study indicates that baicalin alleviates *G. parasuis*-induced immunosuppression and might promote CD163/TWEAK-mediated autophagy. This finding suggests that baicalin could serve as a potential therapeutic agent to control *G. parasuis infection* and related vascular inflammation.

## 1. Introduction

*Glaesserella parasuis* (*G. parasuis*) is a Gram-negative bacterium that colonizes the pig upper respiratory tract [1]. *G. parasuis* is widely distributed in pig farms and is responsible for Glässer’s disease [2]. This disease can cause significant morbidity and mortality, leading to serious economic losses in piglet raising. The typical characteristics of Glässer’s disease are fibrinous polyserositis, pneumonia, arthritis, and meningitis [3]. According to the agar gel diffusion serotyping method, *G. parasuis* can be classified into 15 serotypes, but up to 20% of isolates cannot be typed [4]. Serovar 5 was considered the highly virulent strain [5]. The pathogenic mechanism of *G. parasuis* remains unclear at present, and thus, understanding the molecular mechanisms of *G. parasuis* infection is crucial for developing better prevention and control strategies.

*G. parasuis* infection in piglets can lead to immunosuppression of the host, but the immunosuppression mechanism remains unclear. CD163 is a scavenger receptor that is predominantly expressed on the surface of macrophages [6]. CD163 has important functions in hemoglobin–haptoglobin complex recognition and clearance [7]. Previous research reported that CD163 participates in various pathological processes, such as inflammation and immune responses [8]. Tumor necrosis factor-like weak inducer of apoptosis (TWEAK) is a member of the tumor necrosis factor superfamily [9]. CD163 can act as a decoy receptor for TWEAK, modulating TWEAK-induced signaling pathways [10]. The crosstalk of CD163 and TWEAK has been implicated in immunosuppression [11]. CD163-expressing macrophages could be affected by TWEAK, as they can internalize TWEAK-bound complexes and contribute to the immunosuppressive environment [12]. Studies have shown that the engagement of TWEAK with FN14 can regulate the efficacy of immune cells, such as macrophages and T cells, leading to the inhibition of immune responses and the progression of various diseases [13]. The TWEAK/Fn14 signaling pathway participates in multiple biological activities, such as angiogenesis and inflammatory responses [14,15]. However, whether CD163/TWEAK is involved in *G. parasuis*-induced immunosuppression has not been investigated.

Autophagy is a cellular process that involves the degradation and recycling of cellular components [16]. Recent studies have suggested that CD163 participates in modulating autophagy [17]. TWEAK regulates the expression of autophagy-related genes or proteins, leading to changes in autophagic flux [18]. However, whether CD163 interacts with TWEAK and regulates autophagy has not been explored in *G. parasuis*-infected piglets.

Baicalin was extracted from the root of *Scutellaria baicalensis* Georgi and has been reported to have important biological functions, such as antioxidant activity, antibacterial and antiviral effects, and the regulation of the immune system [19]. Baicalin has also shown great potential in the treatment of inflammatory diseases [20]. Research has reported that baicalin reduces pulmonary inflammation and injury by attenuating the TLR4/NF-κB pathway in Mycoplasma pneumoniae-infected mice [21]. Baicalin suppresses macrophage JNK-mediated adipose tissue inflammation [22]. Baicalin inhibits monosodium urate crystal-induced pyroptosis in a renal tubular epithelial cell line through Panx-1/P2 × 7 pathways [23]. In addition, baicalin reduces cell damage and affects the H6N6-induced autophagy level of RAW264.7 cells [24]. Baicalin induces the death of non-small cell lung cancer cells via MCOLN3-mediated lysosomal dysfunction and autophagy blockage [25]. Baicalin attenuates diabetic cardiomyopathy by inhibiting autophagy and cell death through SENP1/SIRT3 signaling pathway activation [25]. Thus, it is very interesting to investigate whether baicalin could regulate *G. parasuis*-induced autophagy.

In this study, we performed a detailed study of the efficacy of baicalin in the inhibition of *G. parasuis*-elicited immunosuppression by promoting CD163/TWEAK-mediated autophagy. It was reported that baicalin could regulate *G. parasuis*-induced autophagy, which might be regulated by CD163/TWEAK in piglets, and our results might provide new strategies to prevent *G. parasuis* infection in the clinic.

## 2. Materials and Methods

### 2.1. Ethics Statement

The animal study was approved by the Animal Care and Use Committee of Wuhan Polytechnic University, Hubei Province, China (WPU202308003), approval date 2 August 2023. All piglets used in this study were euthanized at the end of the experiment.

### 2.2. Bacterial

The serovar 5 *G. parasuis* SH0165 isolate used in this study is a highly virulent strain, which was isolated from the lung of a commercial pig with typical arthritis, fibrinous polyserositis, hemorrhagic pneumonia, and meningitis [26]. The SH0165 isolate was plated on TSA plates (Difco Laboratories, USA) or cultured in TSB culture (Difco Laboratories, USA). The culture was supplemented with 10 μg/mL of NAD (Biofroxx, Guangzhou, Guangzhou Saiguo biotech Co., Ltd.) and 10% fetal bovine serum (Sijiqing, Hangzhou, China) at 37 °C.

### 2.3. Drugs

Baicalin used in this study was obtained from Sichuan Xieli Pharmaceutical Co., Ltd. (Pengzhou, China). The drug levamisole was purchased from Beijing Solarbio Science & Technology Co., Ltd. (Beijing, China).

### 2.4. Animal Experiment Design 1

Twenty 30-day old naturally farrowed early-weaned (NFEW) Duroc × Landrace × Large White piglets weighing nine to ten kg were obtained from Wuhan Fenglongxin Breeding Professional Cooperative (Wuhan, China). Twenty piglets were randomly divided into control and infection groups, each comprising ten piglets. The infection group piglets were intraperitoneally infected with 1 × 10^8^ CFU *G. parasuis* resuspended in 1 mL of TSB. The control group piglets were intraperitoneally challenged with an equivalent volume of TSB. All piglets from each group were monitored for three days following challenge by *G. parasuis.* After being challenged at 24 h, 48 h, and 72 h, blood samples were collected for routine hematological and biochemical parameter determination using commercial kits (Shanghai Kehua Bio-Engineering Co., Ltd., Shanghai, China) on a Hitec7100 automatic blood analyzer (Hitachi, Japan) [27].

### 2.5. Histopathological Analysis

After being challenged for three days by *G. parasuis*, the piglets were euthanized, and the aortae were obtained for histopathological analysis. The aortic samples were fixed using immersion in 10% neutral buffered formalin. The 4 μm tissue sections were stained using hematoxylin and eosin [28], then the stained sections were examined with a BX43 light microscope (Olympus, Tokyo, Japan).

### 2.6. Flow Cytometry

To explore the effect of *G. parasuis* on T cell differentiation, the blood was collected at 24 h, 48 h, and 72 h after *G. parasuis* challenge. The collected blood was treated with RBC lysis buffer (TaKaRa, Dalian, China) to remove erythrocytes prior to staining. Following filtration to a single-cell suspension using a 200 μm filter, the samples were stained with mouse anti-porcine CD3ε-FITC (Southern Biotech, Birmingham, AL, USA), mouse anti-porcine CD4-SPRD (Southern Biotech), and mouse anti-porcine CD8a-PE (Southern Biotech). The blood samples were repeated at least three times, and the data were analyzed using CytExpert SRT software.

### 2.7. Proteome Analysis

The piglet aorta tissue samples from the control and the infection group were obtained and immediately put into liquid nitrogen for proteome analysis [29]. Briefly, the blood vessel samples were added to lysis buffer. After collecting supernatant, the protein concentration of the supernatant was assessed using BCA protein assay. Sequence-grade modified trypsin (Promega, Madison, WI, USA) was used to obtain the peptide mixture. The obtained peptides were analyzed using LC-MS/MS with Orbitrap Fusion™ Lumos™ Tribrid™ coupled with the EASY-nLC 1200 system (Thermo Fisher Scientific, MA, USA). Differentially expressed proteins were filtered with the standard of fold change >1.5 and Q value < 0.05. The obtained genes were mapped to the KEGG database by employing FDR ≤ 0.05 as the threshold.

### 2.8. Animal Experiment Design 2

This animal experiment was reported in a previous study [27]. Briefly, sixty 30-day NFEW piglets weighing nine to ten kg were randomly divided into six groups, including the control, infection, levamisole, 25 mg/kg of baicalin, 50 mg/kg of baicalin, and 100 mg/kg of baicalin groups. Before *G. parasuis* challenge, the levamisole, 25 mg/kg of baicalin, 50 mg/kg of baicalin, and 100 mg/kg of baicalin groups were treated via intramuscular injection with 15 mg/kg of body weight (BW) of levamisole, 25 mg/kg of BW of baicalin, 50 mg/kg of BW of baicalin, and 100 mg/kg of BW of baicalin, respectively. Two hours after administration on the first day, all treatment groups except for the control group were intraperitoneally infected with 1 × 10^8^ CFU *G. parasuis* resuspended in one mL of TSB. The control group received one mL of TSB only intraperitoneally. Hence, the five groups, except the control group, used intramuscular injection with the same drugs after being challenged for six hours. Each treatment was administered for two days and was adopted twice a day. The piglets were observed for seven days following *G. parasuis* infection.

### 2.9. RT-PCR(Reverse Transcription-Polymerase Chain Reaction)

The piglet aorta tissue samples were collected for gene expression level determination by using RT-PCR [30]. Briefly, the total RNA from the blood vessels was extracted by using TRIzol reagent (Invitrogen, USA). The concentration of the isolated RNA was measured on a Qubit 2.0 fluorometer (Thermo Fisher Scientific, USA). Following reverse transcription into complementary DNA (cDNA) using reverse transcriptase (TaKaRa, Dalian, China), the RT-PCR was carried out by using a SYBR Green PCR Kit (TaKaRa) based on the manufacturer’s instructions. Each sample transcription was repeated at least three times. The internal gene adopted in this study was *GAPDH*. The relative expression levels of the gene were evaluated by employing the threshold cycle (CT) method. The gene relative expression fold changes were measured by employing the 2^−ΔΔCT^ CT formula. The primers for RT-PCR used in this study are presented in Table 1.

### 2.10. Western Blot

The levels of protein expression were assessed by using Western blot [31]. Briefly, aorta total proteins were isolated using RIPA lysis buffer. The concentration of proteins was determined by using a BCA protein assay kit (Beyotime Biotechnology, China). Following transfer onto PVDF membranes (Millipore, USA), the proteins were separated by utilizing 8 to 12% concentration of SDS-PAGE. The membranes were blocked with 15% nonfat milk and incubated with anti-CD163 (#16646-1, 1:1000, Protenitech), anti-JAG1 (#WL03261, 1:1000, Wanleibio), anti-Notch1 (#A19090, 1:1500, ABclonal), anti-HES1 (#A11718,1:1000, ABclonal), anti-WNT3A (#A0642, 1:1000, ABclonal), anti-GSK-3β (#A2081, 1:2000, ABclonal), anti-RBP-J (#WL04414, 1:1000, Wanleibio), anti-c-Myc (#HA721182, 1:1000, Huaan Biotechnology), anti-P62 (#A19700, 1:40,000, ABclonal), anti-LC3B (#A19665, 1:4000, ABclonal), anti-Beclin1 (#A21191, 1:10,000, ABclonal), anti-Caspase1 (#A0964, 1:1000, ABclonal), anti-NLRP3 (#A24294, 1:1000, ABclonal), and anti-β-catenin antibodies (#51067-2, 1:2000, Protenitech), respectively, overnight at 4 °C. After washing five times with TBST, the membranes were incubated with a second antibody, HRP Goat-Anti Rabbit IgG (1:10,000, Abbkine), for 60 min under 37 °C. The band signals of proteins were detected using an enhanced chemiluminescence detection kit (ABclonal, Wuhan, China). The protein expression level data were evaluated using a FluorChemFC2 AIC system (Alpha Innotech, USA).

### 2.11. Detection of CD163 Expression by Immunohistochemistry

The CD163 expression level was also measured using immunohistochemistry analysis. Briefly, aorta samples were prepared from paraffin-embedded blocks. The slices were cut and incubated with anti-CD163 (#16646-1, 1:500, protenitech) immunohistochemistry (IHC) at 4 °C overnight. CD163 was detected with HRP goat anti-rabbit secondary antibody and visualized using diaminobenzidine (DAB) (Servicebio, Wuhan, China). The negative control received only the second antibody. Following counterstaining with hematoxylin (Servicebio, Wuhan, China) for three min, microscopic examination was carried out with a BX43 light microscope, and Image 1.52a software was employed to determine the optical density sum of the brown area.

### 2.12. Statistical Analysis

The experimental data were displayed as mean ± SD. Differences were analyzed using analysis of variance (ANOVA). A value of *p* < 0.05 displays statistical significance.

## 3. Results

### 3.1. G. parasuis Altered the Routine Blood Indicators and Biochemical Parameters, Increased Cytokines Production, and Induced Blood Vessel Tissue Damage

**Figure 1:**
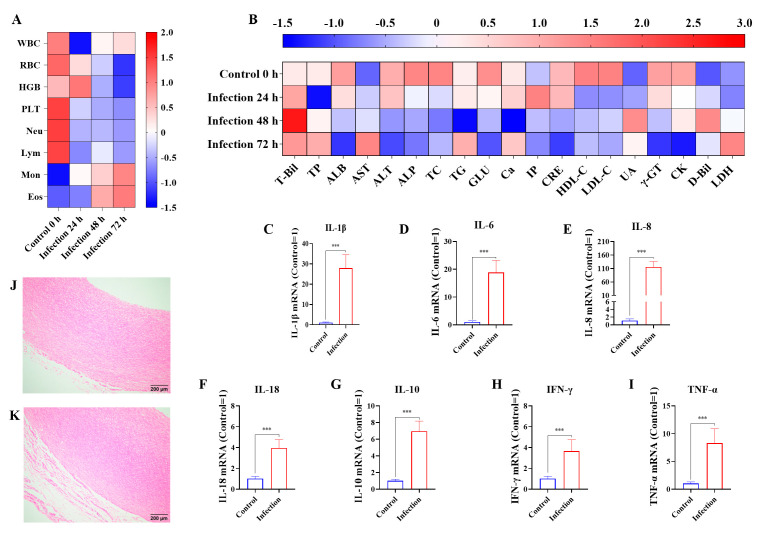
The effects of *G. parasuis* on the routine blood indicators, biochemical parameters, cytokine production, and blood vessel tissue damage of piglets. On 24 h, 48 h, 72 h infected by *G. parasuis*, the blood were collected and the routine blood indicators (**A**) and biochemical parameters (**B**) were determined. At 72 h after being challenged by *G. parasuis*, the mRNA levels of IL-1β (**C**), IL-6 (**D**), IL-8 (**E**), IL-18 (**F**), IL-10 (**G**), IFN-γ (**H**), and TNF-α (**I**) in the blood vessels were assessed using RT-PCR, and the blood vessels from the control group (**J**) and the infection group (**K**) were obtained for pathological analysis. Hemoglobin: HGB; eosinophils: Eos; total protein: TP; total cholesterol: TC; triglycerides: TG; calcium: Ca; uric acid: UA; *** *p* < 0.001.

Compared to the control group, for those infected by *G. parasuis* for 24 h, the white blood cells (WBCs), red blood cells (RBCs), platelets (PLTs), neutrophils (Neus), and lymphocytes (Lyms) were significantly reduced in the infection group, while the monocytes (MONs) were raised in the infection group (*p* < 0.05), as shown in Figure 1A. At 72 h, Lym and Neu continued to reduce in the infection group compared to the control group (*p* < 0.05).

At 24 h, albumin (ALB), glucose (GLU), high-density lipoprotein cholesterol (HDL-C), γ-glutamyl transpeptidase (γ-GT), and creatine kinase (CK) levels were reduced, whereas total bilirubin (T-Bil), aspartate aminotransferase (AST), inorganic phosphate (IP), and direct bilirubin (D-Bil) were raised in the infection group (*p* < 0.05), as shown in Figure 1B. At 72 h, T-Bil, AST, D-Bil, and lactate dehydrogenase (LDH) levels were increased, and the levels of ALB, alanine aminotransferase (ALT), alkaline phosphatase (ALP), GLU, creatinine (CRE), low-density lipoprotein cholesterol (LDL-C), γ-GT, and CK were decreased in the infection group compared to the control group (*p* < 0.05).

At 72 h after being infected by *G. parasuis*, the mRNA levels of IL-1β, IL-6, IL-8, IL-10, IL-18, IFN-γ, and TNF-α in the blood vessels were upregulated in the infection group compared to the control group (*p* < 0.001), as shown in Figure 1C–I. Pathological analysis of vessel tissue indicated that the vessels from the infection group underwent serious tissue damage with massive inflammatory cell infiltration and severe hemorrhage, while some minor tissue damage was present in the control group, as seen in Figure 1J,K.

### 3.2. G. parasuis Reduced the Proportion of CD3+, CD3+CD4+, and CD3+CD8+ T Cells in the Blood of Piglets

**Figure 2:**
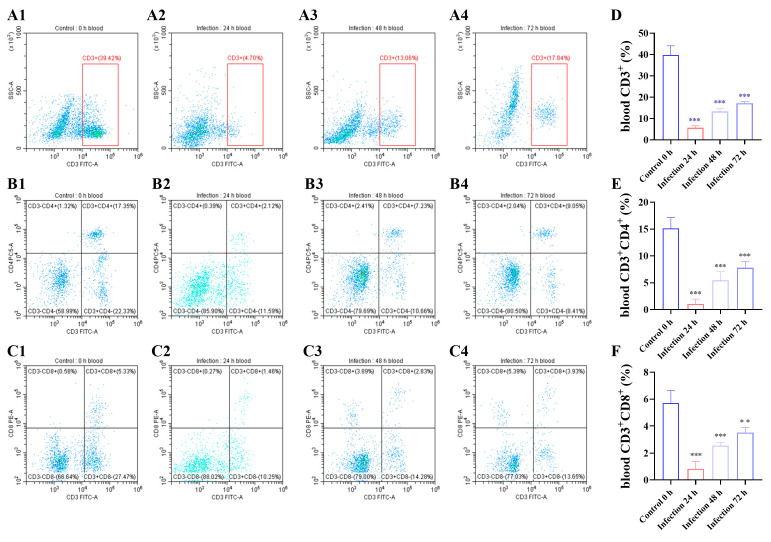
Following infected by *G. parasuis* at 24 to 72 h, piglet bloods were collected to determine CD3+, CD3+CD4+, and CD3+CD8+ T cell proportions. (**A1**–**A4**,**D**): the CD3+ T cells; (**B1**–**B4**,**E**): the CD3+ CD4+ T cells; (**C1**–**C4**,**F**): the CD3+ CD8+ T cells; ** *p* < 0.01; *** *p* < 0.001.

The results showed that compared to the control group at 24 h after infection with *G. parasuis*, the CD3+, CD3+CD4+, and CD3+CD8+ T cell proportions were reduced in the infection group (*p* < 0.001), as shown in Figure 2. The CD3+, CD3+ CD4+, and CD3+ CD8+ T cell proportions increased from 48 h to 72 h.

### 3.3. G. parasuis Infection Dysregulated Proteins in the Blood Vessels of Piglets

**Figure 3:**
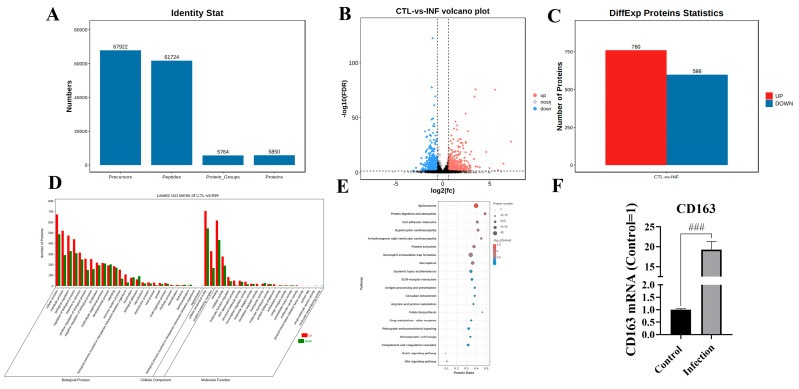
Proteomic analysis of piglet blood vessels after *G. parasuis* challenge. (**A**) The quantified proteins from the proteomic analysis; (**B**) volcano plot of the dysregulated proteins; (**C**) differentially expressed proteins (DEPs) in the infection group and the control group; (**D**) GO enrichment analysis of the DEPs; (**E**) KEGG analysis of the differentially expressed proteins; (**F**) detection of the CD163 expression level by RT-PCR; INF: the infection group; CTL: the control group; ^###^ *p* < 0.001.

Proteomics was used to evaluate the host’s immunosuppression mechanism triggered by *G. parasuis* in the blood vessels of piglets. The data indicated that 61,724 peptides were obtained, which corresponds to 5764 protein groups, as shown in Figure 3A. As shown in Figure 3B,C, 1358 differentially expressed proteins were identified from the blood vessels of the infection group, 760 differentially expressed proteins were upregulated, and 598 differentially expressed proteins were diminished according to the standard of |fold change| > 1.5 and Q value < 0.05. The CD163 expression level was significantly upregulated in the infection group, as shown in Figure 3B,F. Gene Ontology (GO) enrichment analysis demonstrated that the differentially expressed proteins were mainly enriched in the cellular process, metabolic process, and biological regulation from the biological processes category, as shown in Figure 3D. And the differentially expressed proteins were mainly enriched in the cellular anatomical entity and protein-containing complex in the cellular component. In the molecular function category, the differentially expressed proteins were dominantly enriched in binding, catalytic activity, and molecular function regulator, and KEGG analysis showed that the differentially expressed proteins were mainly enriched in spliceosome, necroptosis, cell adhesion molecules, Notch signaling pathway, and Wnt signaling pathway, as shown in Figure 3E.

### 3.4. Baicalin Regulated CD163/TWEAK Axis Expression in Blood Vessels from Piglets Infected by G. parasuis

**Figure 4:**
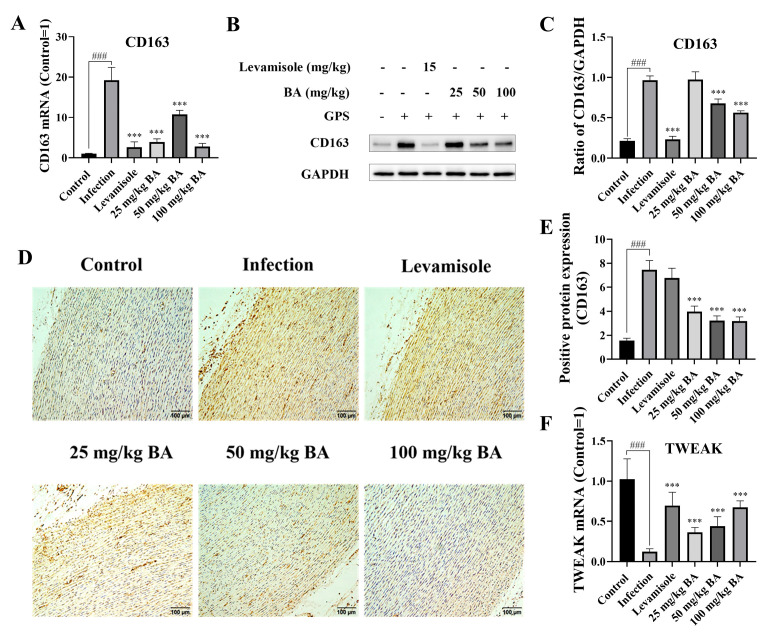
Determination of the function of baicalin on CD163/TWEAK expression in the blood vessels. When the piglets were challenged by *G. parasuis*, the CD163 expression level was assessed using RT-PCR (**A**), Western blot (**B**,**C**), and immunohistochemistry (**D**,**E**). The TWEAK expression level was determined using RT-PCR (**F**). BA: baicalin; ^###^ *p* < 0.001 versus controls; *** significance at *p* < 0.001. Original images can be found in Supplementary Materials.

When the piglets were infected with *G. parasuis*, the CD163 mRNA expression level was significantly increased, and the mRNA level of TWEAK was significantly decreased in the infection group (*p* < 0.001), as shown in Figure 4A,F. Levamisole could attenuate the CD163 mRNA expression level and alter the TWEAK mRNA expression level compared to the infection group (*p* < 0.001). The 25, 50 and 100 mg/kg of baicalin diminished CD163 mRNA expression and increased the TWEAK mRNA expression level from the blood vessels compared to the infection group (*p* < 0.001).

The CD163 protein expression level was determined using Western blot analysis. The data indicated that *G. parasuis* triggered upregulated CD163 protein expression compared to the control group (*p* < 0.001), as shown in Figure 4B,C. Levamisole and 50 to 100 mg/kg of baicalin inhibited the CD163 protein expression level compared to the infection group (*p* < 0.001).

The protein expression level of CD163 was also measured via immunohistochemistry. The results demonstrated that the CD163 protein expression level was raised in the infection group compared to the control group (*p* < 0.001), as shown in Figure 4D,E, while the protein expression level of CD163 was decreased by 25 to 100 mg/kg of baicalin compared to the infection group (*p* < 0.001).

### 3.5. Baicalin Inhibited Notch/Wnt Signaling Pathways Activation in the Blood Vessels from Piglets Challenged by G. parasuis

**Figure 5:**
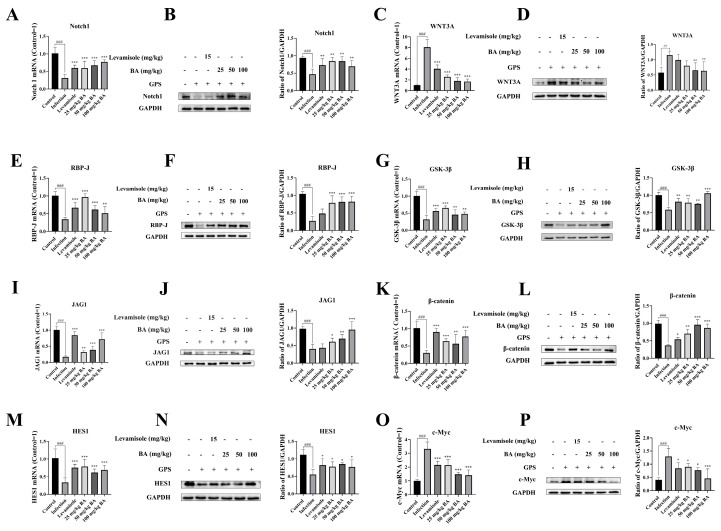
The effects of baicalin on Notch/Wnt signaling pathways’ activation in piglets’ blood vessels. Notch pathway-related molecule expression levels (Notch 1, RBP-J, JAG1, and HES1) and the expression levels of Wnt pathway-related molecules (WNT3A, GSK-3β, β-catenin, and c-Myc) were assessed using RT-PCR and Western blot. (**A**): Notch 1, (**E**): RBP-J, (**I**): JAG1, (**M**): HES1, (**C**): WNT3A, (**G**): GSK-3β, (**K**): β-catenin, (**O**): c-Myc at mRNA level; (**B**): Notch 1, (**F**): RBP-J, (**J**): JAG1, (**N**): HES1, (**D**): WNT3A, (**H**): GSK-3β, (**L**): β-catenin, (**P**): c-Myc at protein level; BA: baicalin; ^##^ *p* < 0.01 versus controls; ^###^ *p* < 0.001 versus controls; * significance at *p* < 0.05; ** significance at *p* < 0.01; *** significance at *p* < 0.001. Original images can be found in Supplementary Materials.

Previous research reported that CD163 could regulate Notch signaling pathway activation [10]. Notch 1 is a key receptor in the Notch 1 signaling pathway. After *G. parasuis* infection of piglets, the mRNA expression level of Notch 1 from the blood vessels was observed to decrease compared to the control group (*p* < 0.001), as shown in Figure 5A. The RBP-J could interact with Notch 1 and adjust the expression of downstream target genes. The blood vessels from the infection group showed a decrease in RBP-J mRNA expression compared to the control group (*p* < 0.001), as shown in Figure 5E. JAG1, a ligand in the Notch signaling pathway, was found to be downregulated at mRNA levels after *G. parasuis* infection (*p* < 0.001), as shown in Figure 5I, which could further enhance the Notch pathway activation. HES1, as the downstream target gene of the Notch signaling pathway, was found to show a decrease in mRNA expression from the blood vessels of the *G. parasuis*-challenged piglets (*p* < 0.001), as shown in Figure 5M, which indicated that the Notch pathway actively regulated its target genes. The levels of Notch 1, RBP-J, JAG1, and HES1 mRNA expression were significantly raised in the vessels from the levamisole group compared to the infection group (*p* < 0.001), as shown in Figure 5A,E,I,M. The use of 25 to 100 mg/kg of baicalin upregulated Notch 1, RBP-J, JAG1, and HES1 mRNA expression levels from the blood vessels compared to the infection group (*p* < 0.01). In the infection group, Notch 1, RBP-J, JAG1, and HES1 protein levels were significantly inhibited compared to the control group, while 25 to 50 mg/kg of baicalin increased the protein expression levels of Notch 1, RBP-J, JAG1, and HES1 compared to the infection group (*p* < 0.05), as shown in Figure 5B,F,J,N.

Compared to the control group, the WNT3A mRNA expression level was raised in the infection group (*p* < 0.001), as shown in Figure 5C. β-catenin is an effector of the Wnt signaling pathway. In the infection group, the mRNA expression level of β-catenin was significantly upregulated in the blood vessels compared to the control group (*p* < 0.001), as shown in Figure 5K. GSK-3β is a kinase that phosphorylates β-catenin, marking it for degradation. The expression of GSK-3β was found to be downregulated after infection (*p* < 0.001), as shown in Figure 5G. The mRNA expression level of c-Myc, as a well-known downstream target gene in the Wnt signaling pathway, was increased from the infection group (*p* < 0.001), as shown in Figure 5O. The mRNA levels of GSK-3β and β-catenin were significantly increased, and WNT3A and c-Myc mRNA levels were significantly downregulated in the levamisole group compared to the infection group (*p* < 0.001), as shown in Figure 5C,G,K,O. The 25 to 100 mg/kg of baicalin could enhance GSK-3β and β-catenin mRNA expressions and attenuate WNT3A and c-Myc mNRA expression levels compared to the infection group (*p* < 0.01).

The protein expressions of WNT3A, GSK-3β, β-catenin, and c-Myc were also assessed using Western blot. The data indicated that *G. parasuis* upregulated WNT3A and c-Myc protein levels and downregulated the GSK-3β and β-catenin protein levels in the infection group (*p* < 0.01), as shown in Figure 5D,H,L,P. Treatment with 25 mg/kg–50 mg/kg of baicalin raised the protein levels of GSK-3β and β-catenin and inhibited the protein levels of WNT3A and c-Myc compared to the infection group (*p* < 0.05).

### 3.6. Baicalin Promoted Autophagy in the Blood Vessels of G. parasuis-Infected Piglets

**Figure 6:**
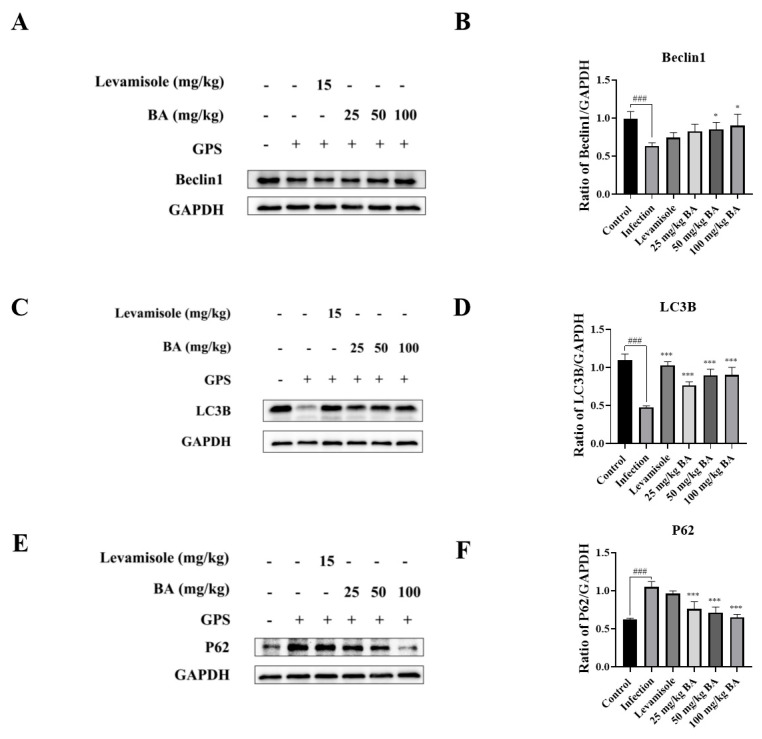
The function of baicalin on autophagy in the blood vessels of *G. parasuis*-infected piglets. Beclin1 (**A**,**B**), LC3B (**C**,**D**), and p62 (**E**,**F**) expression levels were assessed using Western blot. BA: baicalin; ^###^ *p* < 0.001 versus control; * significance present at *p* < 0.05; *** significance present at *p* < 0.001. Original images can be found in Supplementary Materials.

Following infection with *G. parasuis*, the protein levels of Beclin1 and LC3B in the blood vessels of piglets were decreased significantly compared to the control group (*p* < 0.001), as shown in Figure 6A–D. In contrast, the protein expression level of P62 was increased compared to the control group (*p* < 0.001), as shown in Figure 6E,F. When the piglets were treated with levamisole, the protein level of LC3B was upregulated compared to the infection group (*p* < 0.001), as shown in Figure 6C,D. Overall, 50 to 100 mg/kg baicalin could reduce p62 protein levels and promote Beclin1 and LC3B protein expression compared to the infection group (*p* < 0.05), as shown in Figure 6A–D.

### 3.7. Baicalin Inhibited NLRP3/Caspase 1 Signaling Pathway Activation in the Blood Vessels of G. parasuis-Infected Piglets

**Figure 7:**
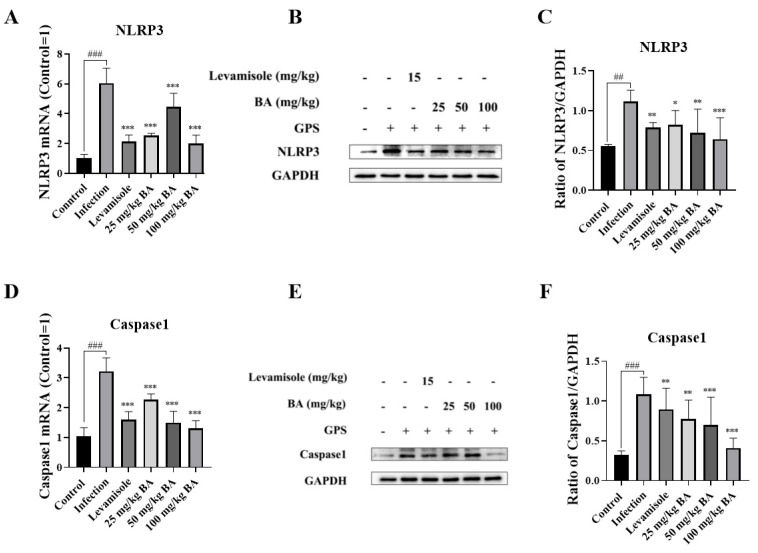
The function of baicalin on NLRP3/Caspase 1 signaling pathway activation in the blood vessels of *G. parasuis*-infected piglets. The NLRP3 and Caspase 1 expression levels were measured using RT-PCR method and Western blot. (**A**): NLRP3 mRNA level; (**B**,**C**): NLRP3 protein level; (**D**): Caspase 1 mRNA level; (**E**,**F**): Caspase 1 protein level; BA: baicalin; ^##^ *p* < 0.01 versus control; ^###^ *p* < 0.001 versus control; * significance present at *p* < 0.05; ** significance present at *p* < 0.01; *** significance present at *p* < 0.001. Original images can be found in Supplementary Materials.

As displayed in Figure 7A,D, the mRNA expression levels of NLRP3 and Caspase 1 in the infection group were raised compared to the control group (*p* < 0.001). Compared to the infection group, levamisole and 25 to 100 mg/kg of baicalin attenuated NLRP3 and Caspase 1 mRNA expression levels (*p* < 0.001). At the protein levels, NLRP3 and Caspase 1 were raised from the infection group compared to the control group (*p* < 0.01), as shown in Figure 7B,C,E,F. Compared to the infection group, treatment with levamisole and 25 mg/kg–50 mg/kg of baicalin reduced the NLRP3 and Caspase 1 expression levels (*p* < 0.05).

### 3.8. Baicalin Decreased Cytokine Production and Diminished Pathological Tissue Damage in Blood Vessels of G. parasuis-Infected Piglets

**Figure 8:**
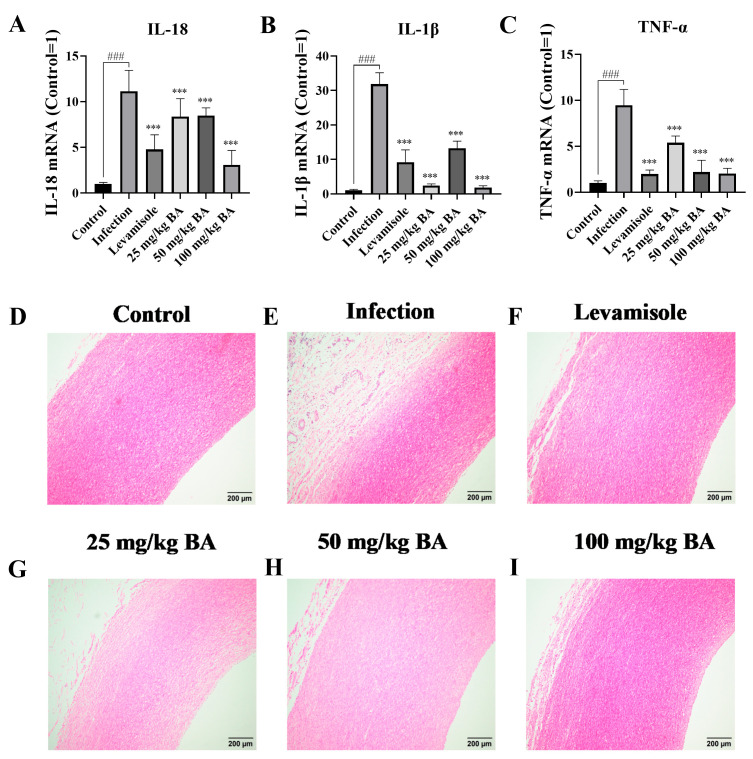
The effect of baicalin on cytokine production and pathological tissue injury in the blood vessels of *G. parasuis*-infected piglets. IL-18 (**A**), IL-1β (**B**), and TNF-α (**C**) expression levels were determined by RT-PCR. The pathological tissue damage assessment in blood vessels was carried out by histopathological analysis. (**D**): the control group; (**E**): the infection group; (**F**): the levamisole group; (**G**): 25 mg/kg of baicalin group; (**H**): 50 mg/kg of baicalin group; (**I**): 100 mg/kg of baicalin group; BA: baicalin; ^###^ *p* < 0.001 versus control; *** significance present at *p* < 0.001.

The data showed that *G. parasuis* significantly induced the mRNA expression levels of IL-1β, IL-18, and TNF-α in the blood vessels (*p* < 0.001), as shown in Figure 8A–C. When the piglets were administered levamisole or baicalin, the mRNA expression levels of IL-1β, IL-18, and TNF-α in the blood vessels were weakened compared to the infection group (*p* < 0.001).

The alleviating effect of baicalin on blood vessel damage was also measured. The data indicated that in the infection group, inflammatory cell infiltration and hemorrhage were detected in the blood vessels (Figure 8E), while there was little injury displayed in the control group (Figure 8D). Only some minor damage was seen in the levamisole group or baicalin groups, as shown in Figure 8F–I.

## 4. Discussion

*G. parasuis* is a significant pathogen in the pig industry, causing Glässer’s disease with significant clinical signs [32]. In this study, *G. parasuis* infection in piglets led to a significant decrease in the proportion of CD3+, CD3+CD4, and CD3+CD8+ T cells in the blood. This indicates that *G. parasuis* could elicit immunosuppression in the host, which has also been reported in previous work [27,29]. It has been reported that human immunodeficiency virus (HIV) primarily attacks CD4+ T cells, CD3+ T cells, and CD8+ T cells, resulting in a sharp decline in numbers and leading to immunosuppression [33]. The imbalance and abnormal function of CD4+ and CD8+ T cells have important functions in the regulation of the development of systemic lupus erythematosus [34]. In the tumor microenvironment, the distribution and function of CD3+, CD4+, and CD8+ T cells also change [35], and tumor cells might suppress T cell activity through immune checkpoint molecule production and immunosuppressive cytokine secretion [36]. It was therefore hypothesized that *G. parasuis* could directly affect the host’s immune response’s contribution to the progression of immunosuppression. The mechanism underlying immunosuppression remains unclear. However, it is crucial to understand for developing effective prevention and control strategies.

Our previous study showed that the PD-1/PD-L1 axis triggered immunosuppression via the PI3K/Akt/mTOR signaling pathway [29]. Whether there are any other molecules that can induce host immunosuppression needs to be explored. It was found that CD163 was significantly upregulated in the vessels of piglets infected by *G. parasuis* through vessel proteomics analysis. CD163 is a scavenger receptor predominantly expressed on macrophages [7]. CD163 acts as a decoy receptor for TWEAK [37], which is considered a multifunctional pro-inflammatory cytokine and is significantly expressed on macrophages [38]. The CD163/TWEAK axis is involved in various pathological processes of disease. The lipid droplet (LD)-laden macrophages (LLMs) were present with immunosuppressive phenotypes, accompanied by extensive expression of CD163, and reduced the antitumor activities of CD8+ T cells [39]. The CD163+ macrophages are thought to counteract tumor immunity by enhancing immunosuppressive mechanisms [40]. In this study, the CD163 expression level was increased, while the mRNA level of TWEAK was decreased in the infection group, suggesting that CD163 and TWEAK may be involved in *G. parasuis*-induced immunosuppression. Baicalin regulated the expression of the CD163/TWEAK axis, indicating that baicalin may alleviate *G. parasuis*-induced immunosuppression by modulating the CD163/TWEAK axis. Further studies are needed to elucidate the detailed mechanism of action of baicalin on the CD163/TWEAK axis, and other pig breeds or ages will also be used to study the function of baicalin on the regulation of CD163/TWEAK.

The CD163/TWEAK axis was reported to be one of the chief regulators of Notch signaling during the inflammation process [41]. The Notch signaling pathway was thought to be related to dextran sodium sulfate-induced colitis in mice [42]. Blocking the Notch signaling pathway reduced PRRSV infection both in vitro and in vivo [43], so the Notch signaling pathway can act as a potential novel therapeutic target for kidney and liver diseases [44,45]. The Wnt signaling has important roles in liver regeneration, tumorigenesis, and cardiovascular disease [46,47]; thus, regulation of Wnt signaling has become an attractive method to control liver disease and cardiovascular disease. However, the mechanism by which the CD163/TWEAK axis might regulate Notch and Wnt signaling pathways in response to *G. parasuis* infection remains largely unexplored. This study notably observed a significant regulation of key components of the Notch and Wnt signaling pathway. The use of levamisole and baicalin provided further insights into the modulation of these pathways. Levamisole, which is known to have immunomodulatory effects, significantly regulated the expression of key Notch and Wnt pathway components. These findings suggest that levamisole and baicalin might have therapeutic potential in modulating the Notch and Wnt signaling pathways in response to *G. parasuis* challenges.

Autophagy, a critical cellular process involving the degradation and recycling of cellular components, plays an important role in pathological conditions [48]. The crosstalk between autophagy and Wnt/Notch signaling controls cardiac differentiation [49]. Notch and Wnt signaling pathways can interact with autophagy and affect the survival and death of tumor cells [50]. Autophagy was involved in mouse kidney development and podocyte differentiation, which was regulated by Notch signaling [51]. Wnt signaling activation regulated autophagy due to a dietary magnesium deficiency in injury-induced osteoarthritis [52]. This study found that when piglets were infected with *G. parasuis*, the Notch and Wnt signaling pathways were altered, and baicalin could regulate Notch/Wnt signaling and autophagy, suggesting that Notch and Wnt signaling pathways may regulate autophagy in *G. parasuis* infection.

## 5. Conclusions

In conclusion, our study showed that baicalin regulated CD163/TWEAK axis expression, inhibited Notch/Wnt signaling pathways’ activation, promoted autophagy, reduced NLRP3/Caspase 1 signaling pathway activation, decreased cytokine production, and alleviated pathological tissue damage in blood vessels. This study provides initial evidence that the CD163/TWEAK axis could induce host immunosuppression, and baicalin promotes autophagy in piglets challenged by *G. parasuis*. Understanding the role of autophagy in *G. parasuis* infection could offer a new view of the mechanism of disease and potential treatment targets to control *G. parasuis* infection in clinical settings. We will focus on exploring the relationship between autophagy and *G. parasuis*-induced immunosuppression in our future studies.

## Figures and Tables

**Table 1:** Primer sequences used for qRT-PCR analysis.

Gene		Nucleotide Sequence (5′-3′)	Tm (°C)	Length (bp)
IL-1β	Forward	TCTGCATGAGCTTTGTGCAAG	59.7	155
	Reverse	ACAGGGCAGACTCGAATTCAAC	60.9	
IL-6	Forward	CTTCTGGTGATGGCTACTG	52.7	134
	Reverse	TTGCCGAGGATGTACTTAA	50	
IL-8	Forward	ACAGCAGTAACAACAACAAG	50.2	117
	Reverse	GACCAGCACAGGAATGAG	53.2	
IL-10	Forward	CGTGGAGGAGGTGAAGAGTG	55.4	178
	Reverse	TTAGTAGAGTCGTCATCCTGGAAG	55.6	
IL-18	Forward	AGTAACCATATCTGTGCAGTGT	54	155
	Reverse	TCTTATCACCATGTCCAGGAAC	53	
TNF-α	Forward	CGCTCTTCTGCCTACTGCACTTC	60.7	164
	Reverse	CTGTCCCTCGGCTTTGACATT	57.8	
Caspase1	Forward	TACAAGAATCCCAGGCGGTG	57.5	128
	Reverse	CCTTTGGGCTATGTCTGGGG	58.6	
NLRP3	Forward	CAGGCTTCTGGGACACCTTT	59.9	110
	Reverse	GTGCAGCCCTAGTCAGAGTC	58.8	
TWEAK	Forward	AGGCCAAGGCAGGCCAGCG	68.1	100
	Reverse	GTCGGAGCCAGAGGCGGAGG	66.8	
HES1	Forward	GTGAGTGCATGAACGAGGT	59.2	118
	Reverse	GTCATGGCGTTGATCTGGGT	60.4	
JAG1	Forward	TTTCAGGGCGACCTTGCATC	61	121
	Reverse	CCACACCACACCTTCGAGC	61	
RBP-J	Forward	CTTGAACTTACAGGACAGAAT	53.2	105
	Reverse	GACGACACAGAGCATACT	53.7	
GSK-3β	Forward	CAGAACCACCTCCTTTGCG	58.8	100
	Reverse	GGTCACCTTGCTGCCATCC	61.1	
GAPDH	Forward	GGCACAGTCAAGGCGGAGAAC	61.9	105
	Reverse	AGCACCAGCATCACCCCATTTG	61	
c-Myc	Forward	CTGCCAAGAGGGCTAAGTT	54.6	135
	Reverse	TCTGGCGTTCCAAGACATT	56	
WNT3A	Forward	TACTCCTCTGCAGCCTGAAGCA	63.4	322
	Reverse	ATGGCGTGGACAAAGGCCGAC	65.8	
Notch1	Forward	GATGGCATCAATTCCTTTAC	52.2	149
	Reverse	TGAGGGCAGGTACACTTGT	53	
β-catenin	Forward	AAGGCAATCCTGAAGAAGA	52	148
	Reverse	ATAGCAGCTCGTACCCTCT	51.4	
CD163	Forward	TGCTGTAGTCGCTGTTCT	55.9	117
	Reverse	ACTTTCACCTCCACTCTTC	54	

## Data Availability

The original contributions presented in this study are included in the article.

## Supplementary Materials

The following supporting information can be downloaded at: https://www.mdpi.com/article/10.3390/biom15050722/s1, File S1: original western blot images.
