# Supplementary material for: Baicalin Alleviates Piglet Immunosuppression Induced by Glaesserella parasuis via Promoting CD163/Tumor Necrosis Factor-like Weak Inducer of Apoptosis-Mediated Autophagy
Source: Biomolecules. 2025 May 15;15(5):722. doi: 10.3390/biom15050722 (PMC12108983; doi:10.3390/biom15050722)

Fig 4

Fig 4. B:

GAPDH (repeat 1)

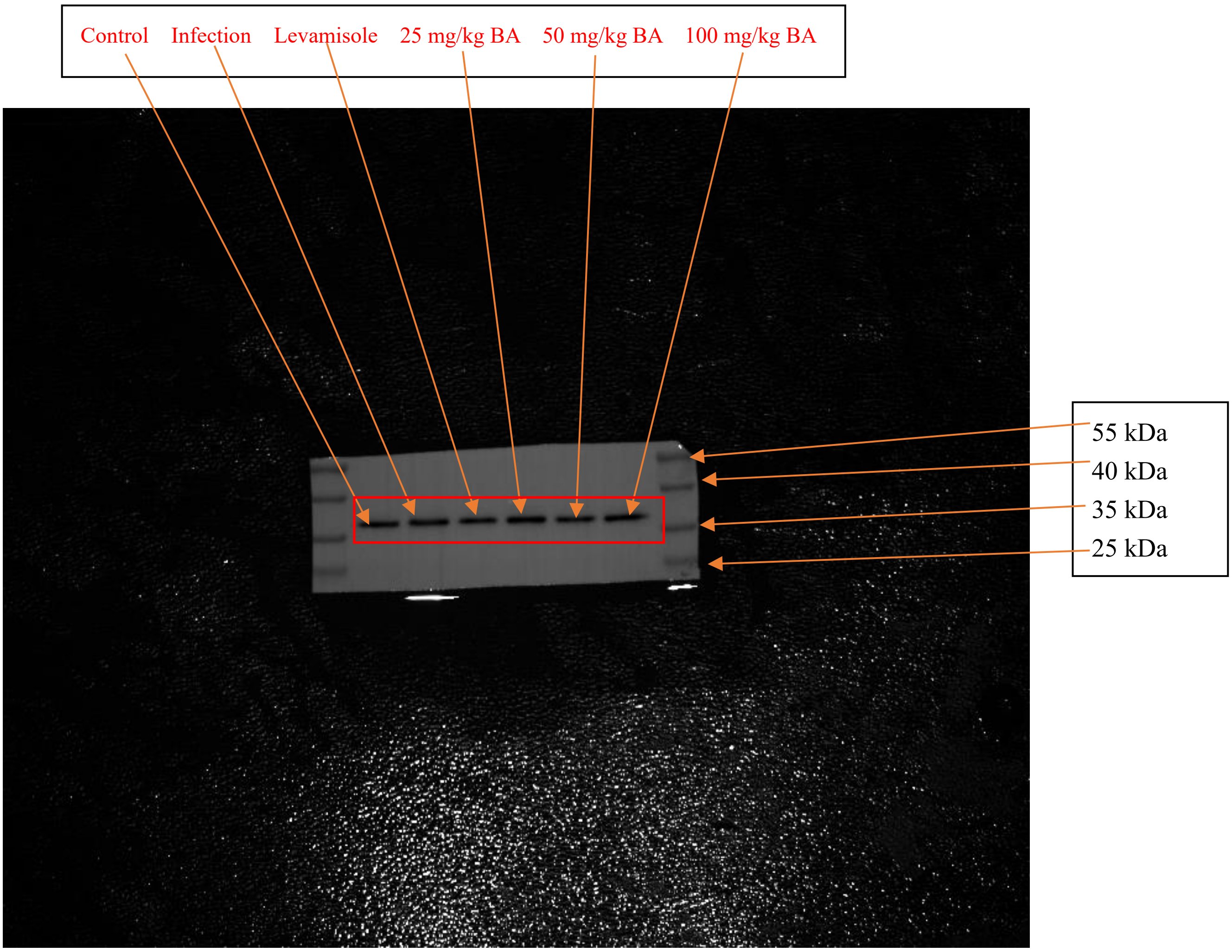

Fig 4

Fig 4. B:

GAPDH (repeat 2)

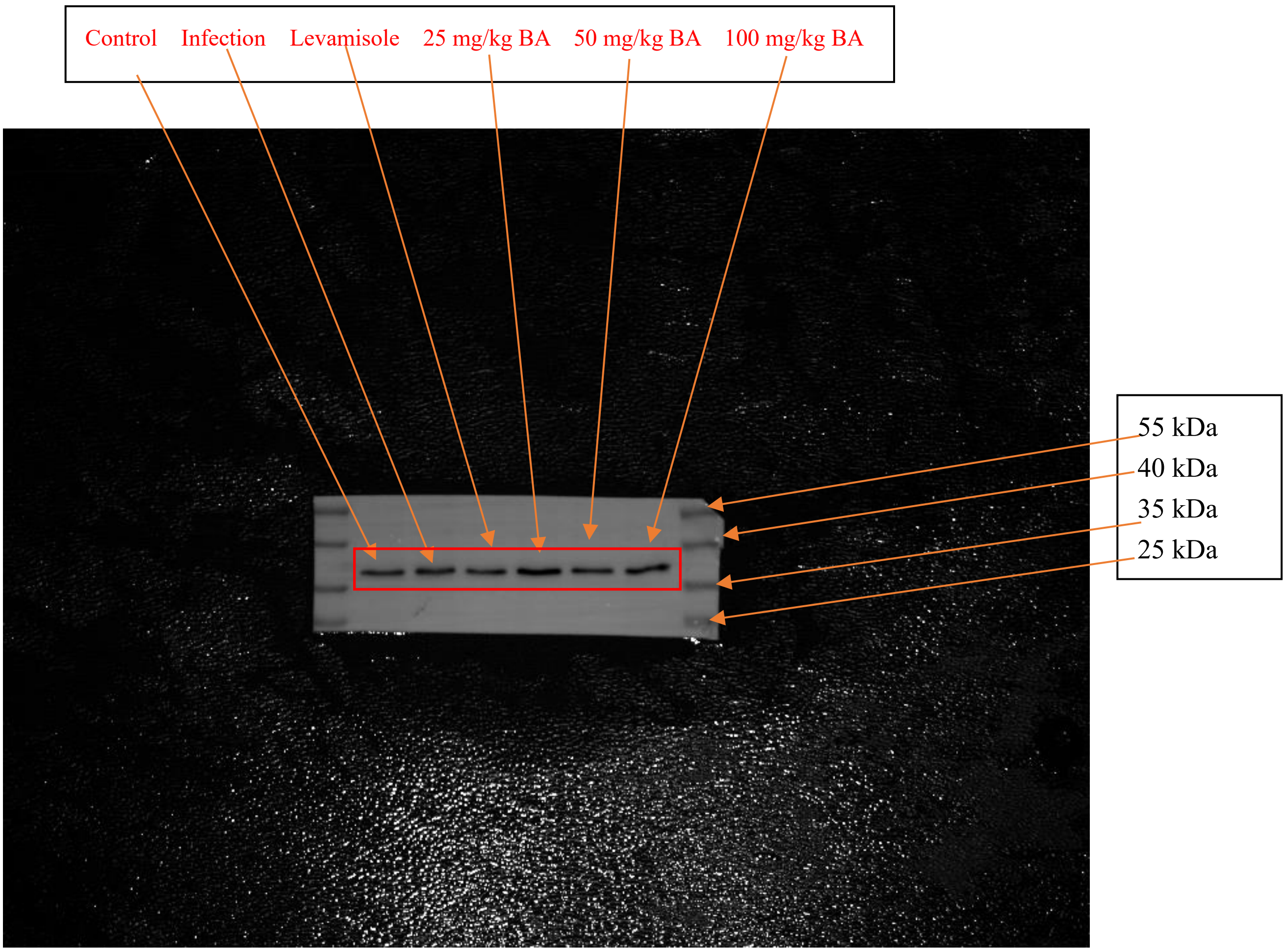

Fig 4

Fig 4. B:

GAPDH (repeat 3)

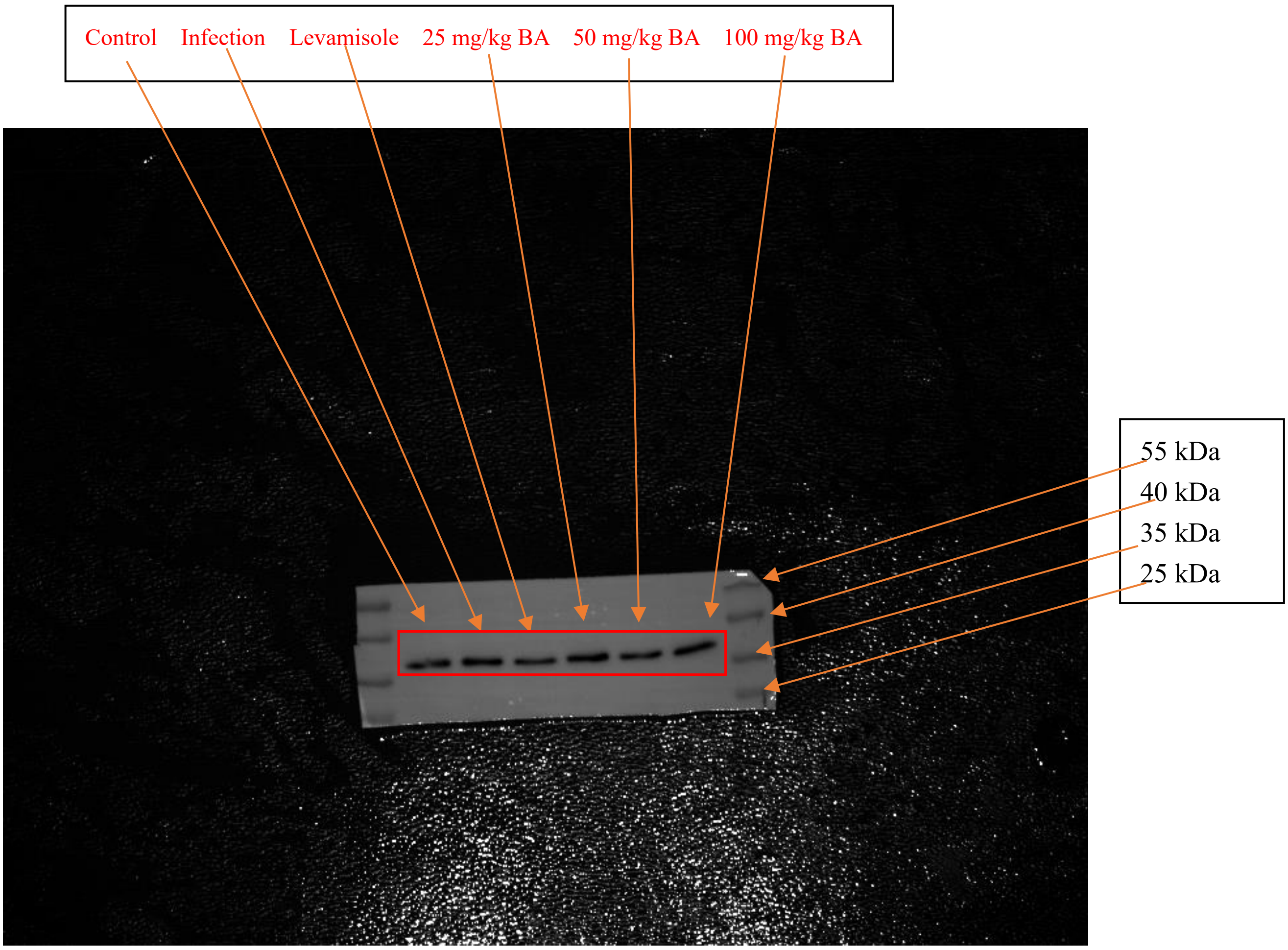

Fig 4

Fig 4. B:

CD163 (repeat 1)

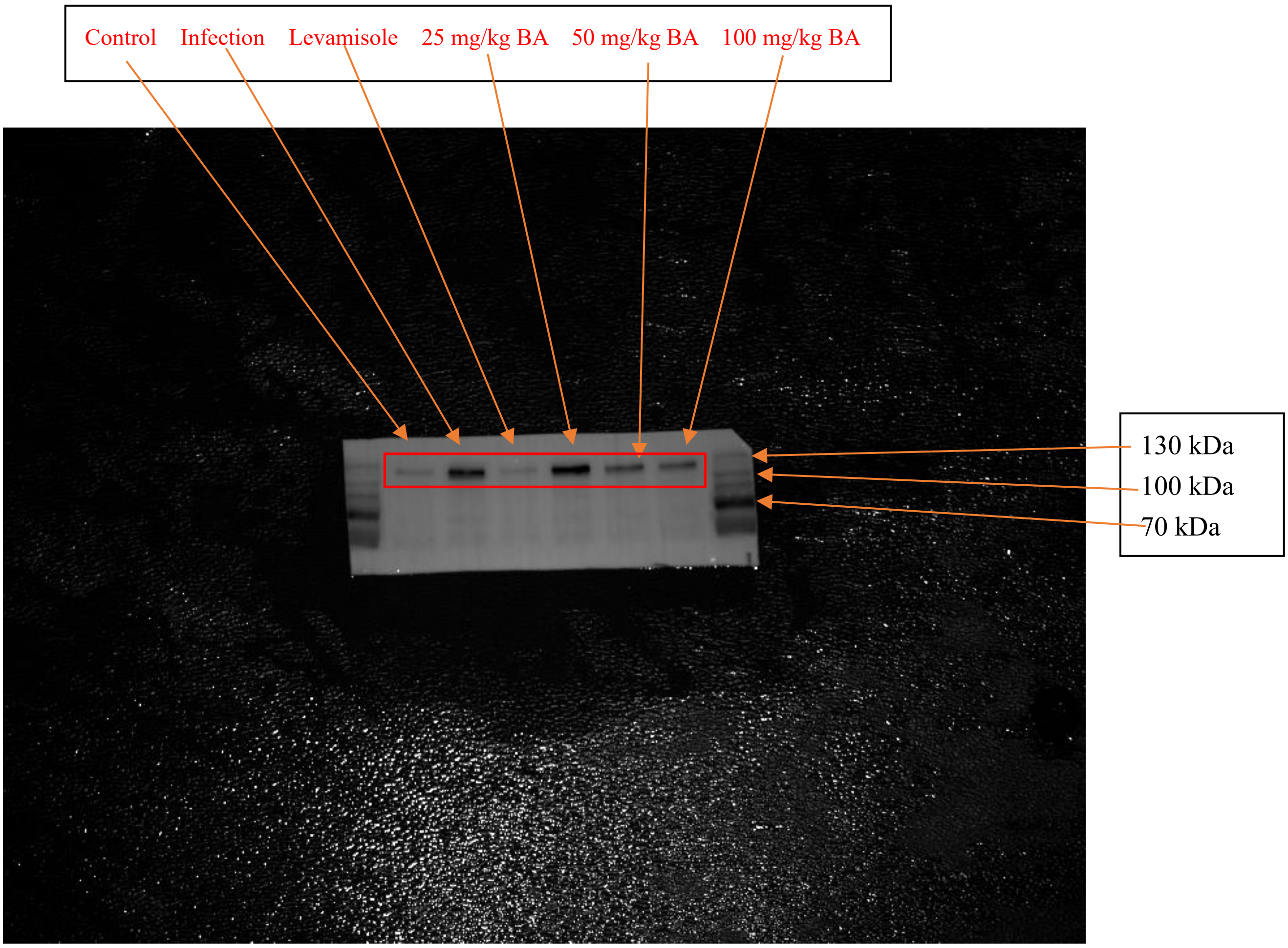

Fig 4

Fig 4. B:

CD163 (repeat 2)

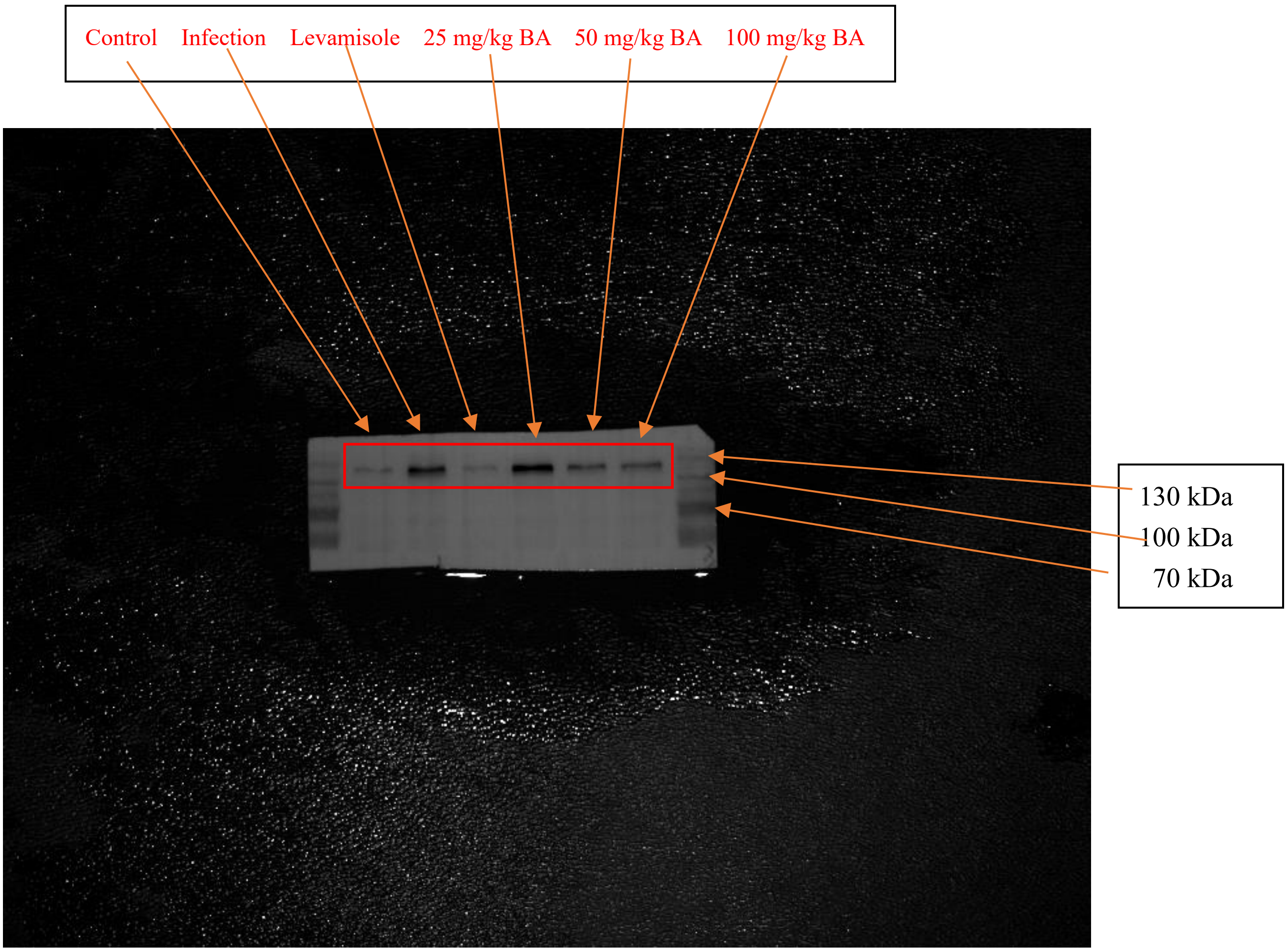

Fig 4

Fig 4. B:

CD163 (repeat 3)

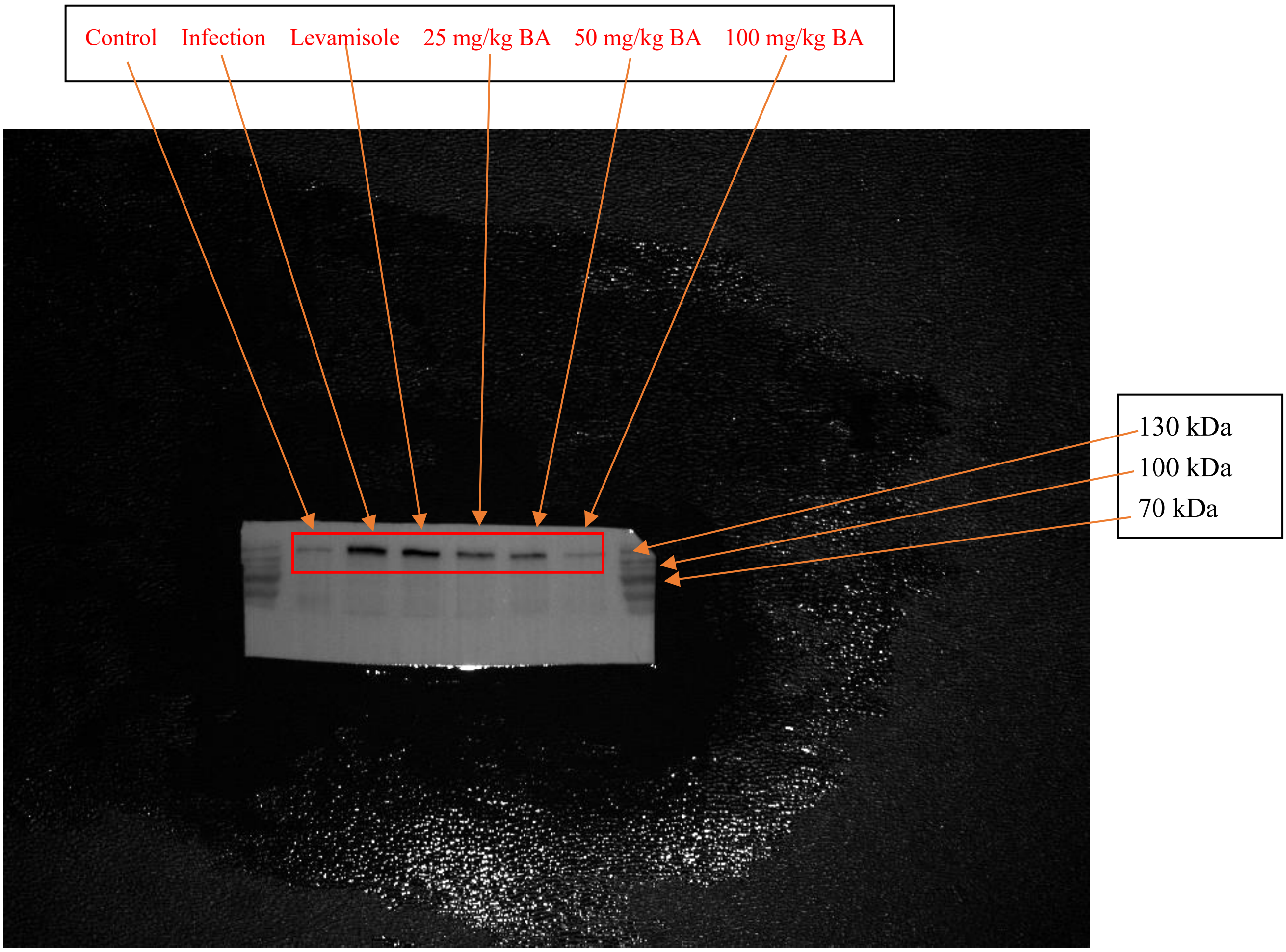

Fig 5

Fig 5. B:

GAPDH (repeat 1)

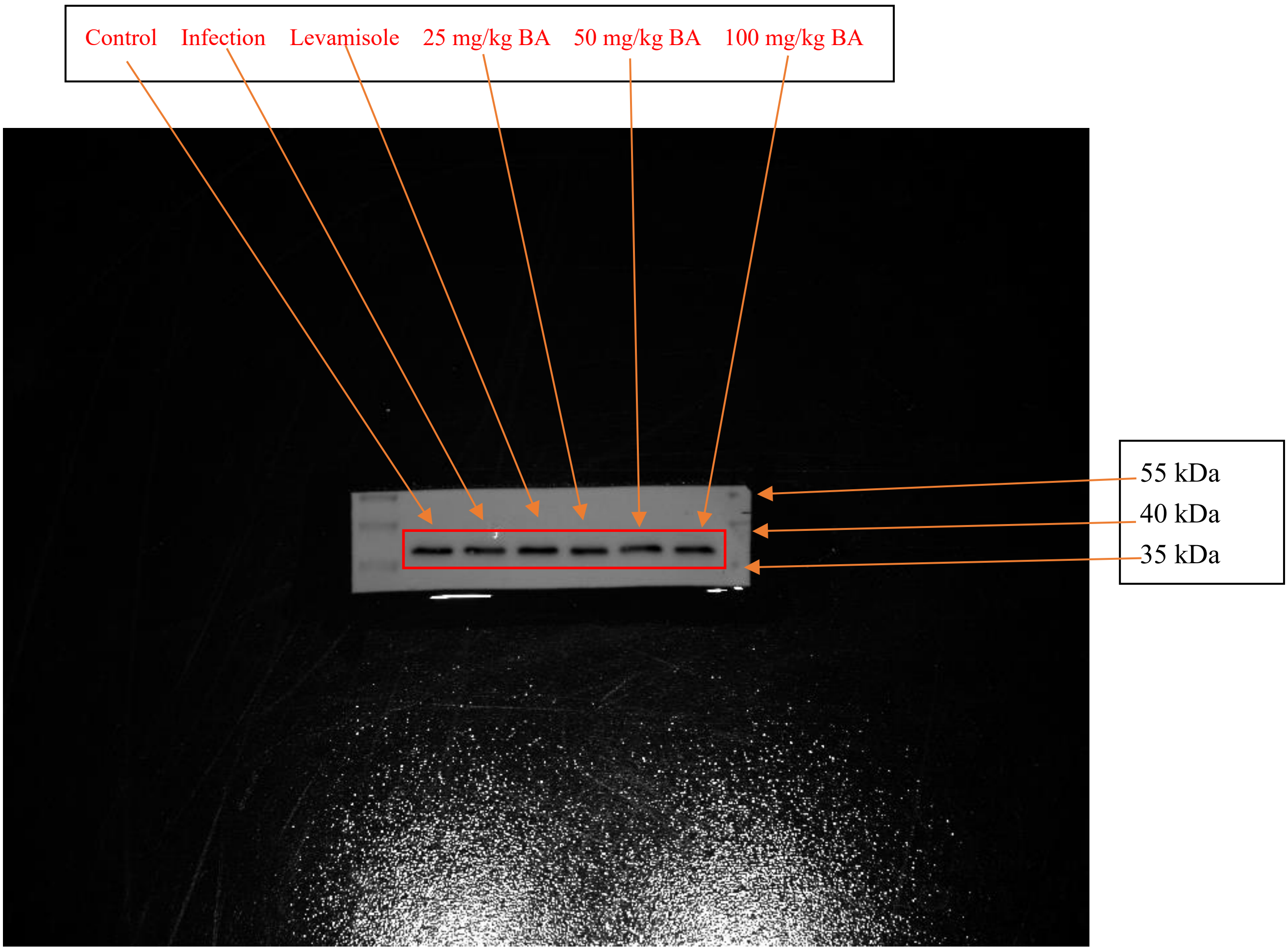

Fig 5

Fig 5. B:

GAPDH (repeat 2)

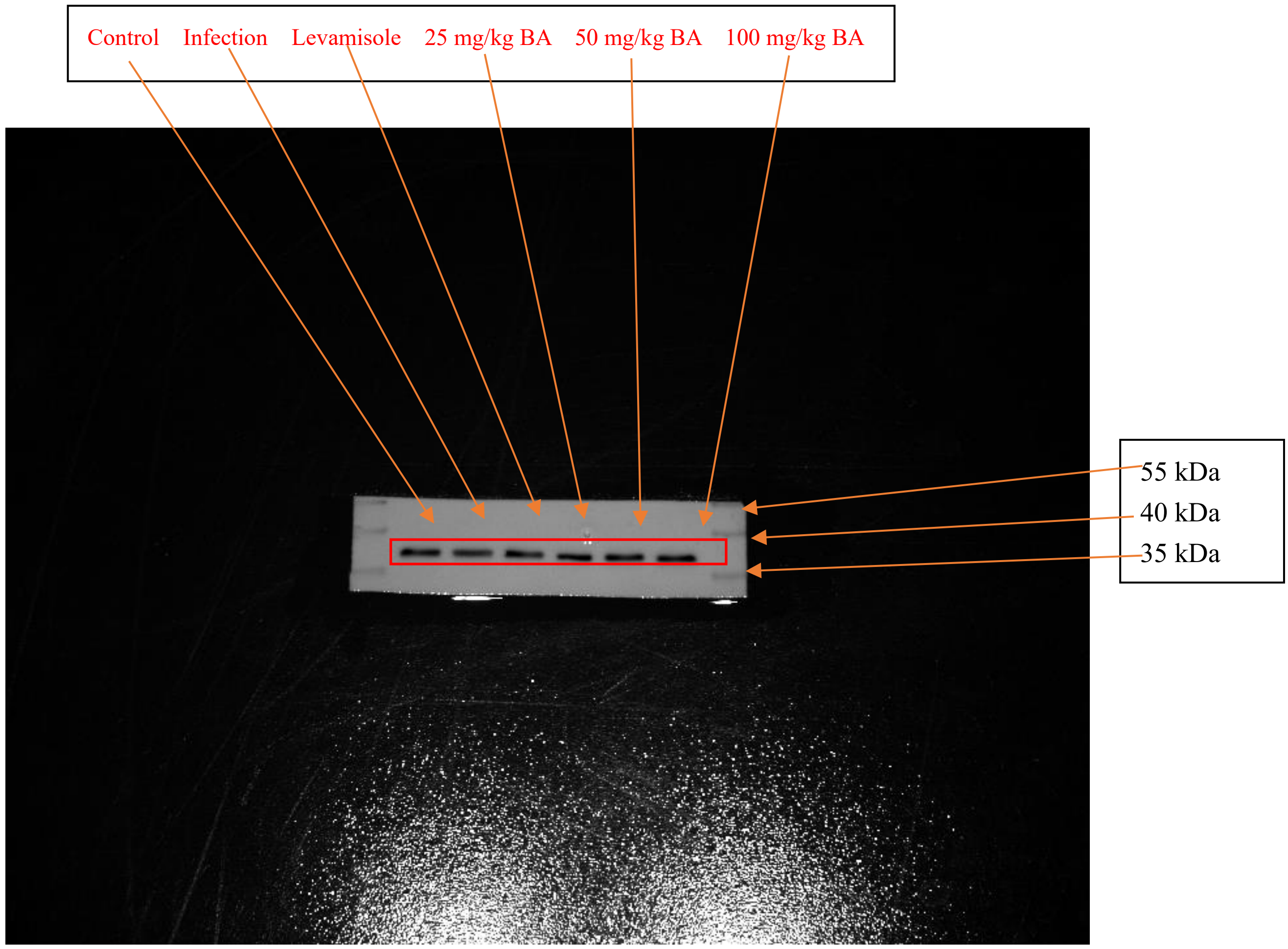

Fig 5

Fig 5. B:

GAPDH (repeat 3)

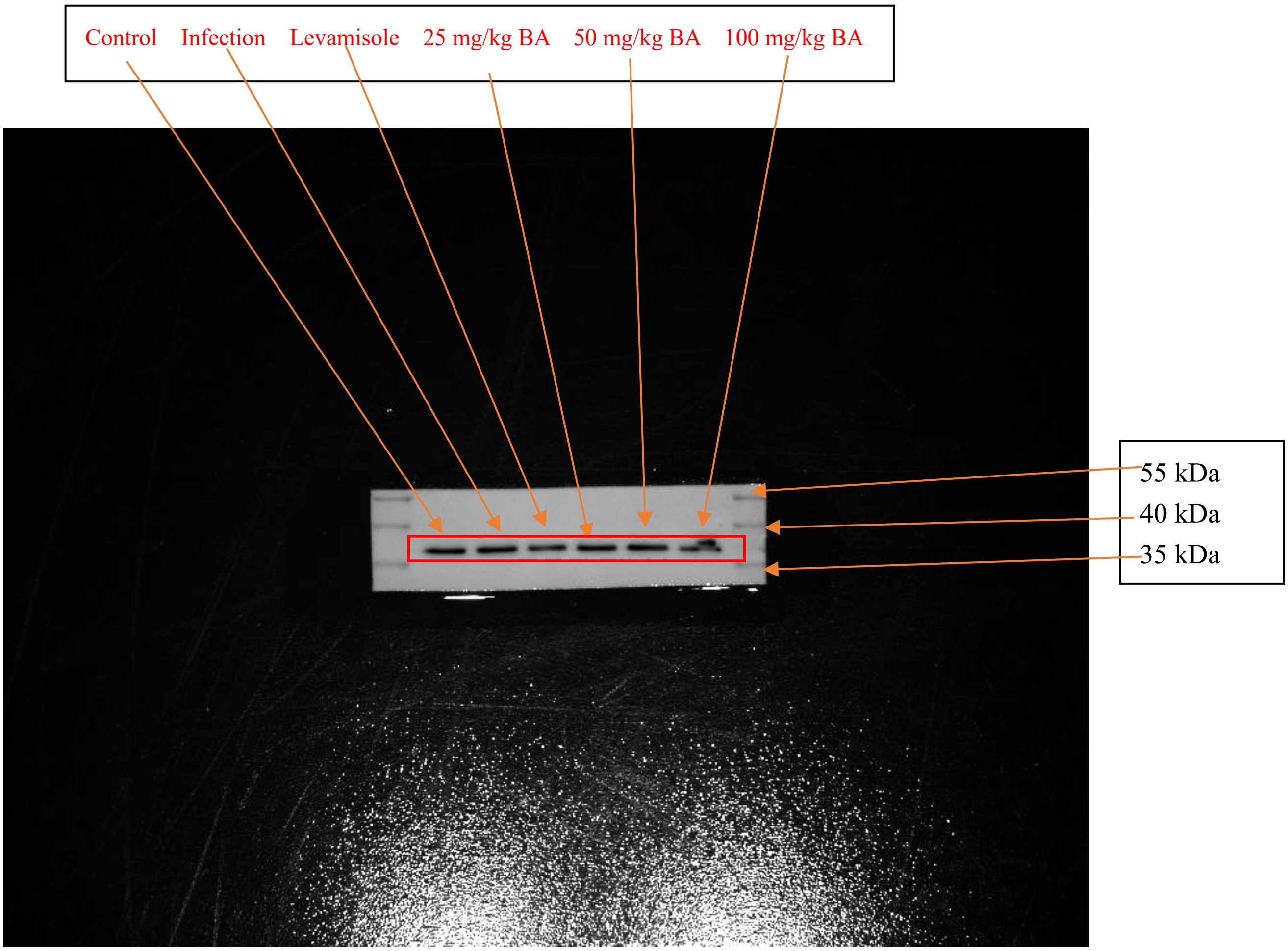

Fig 5

Fig 5. B:

Notch1 (repeat 1)

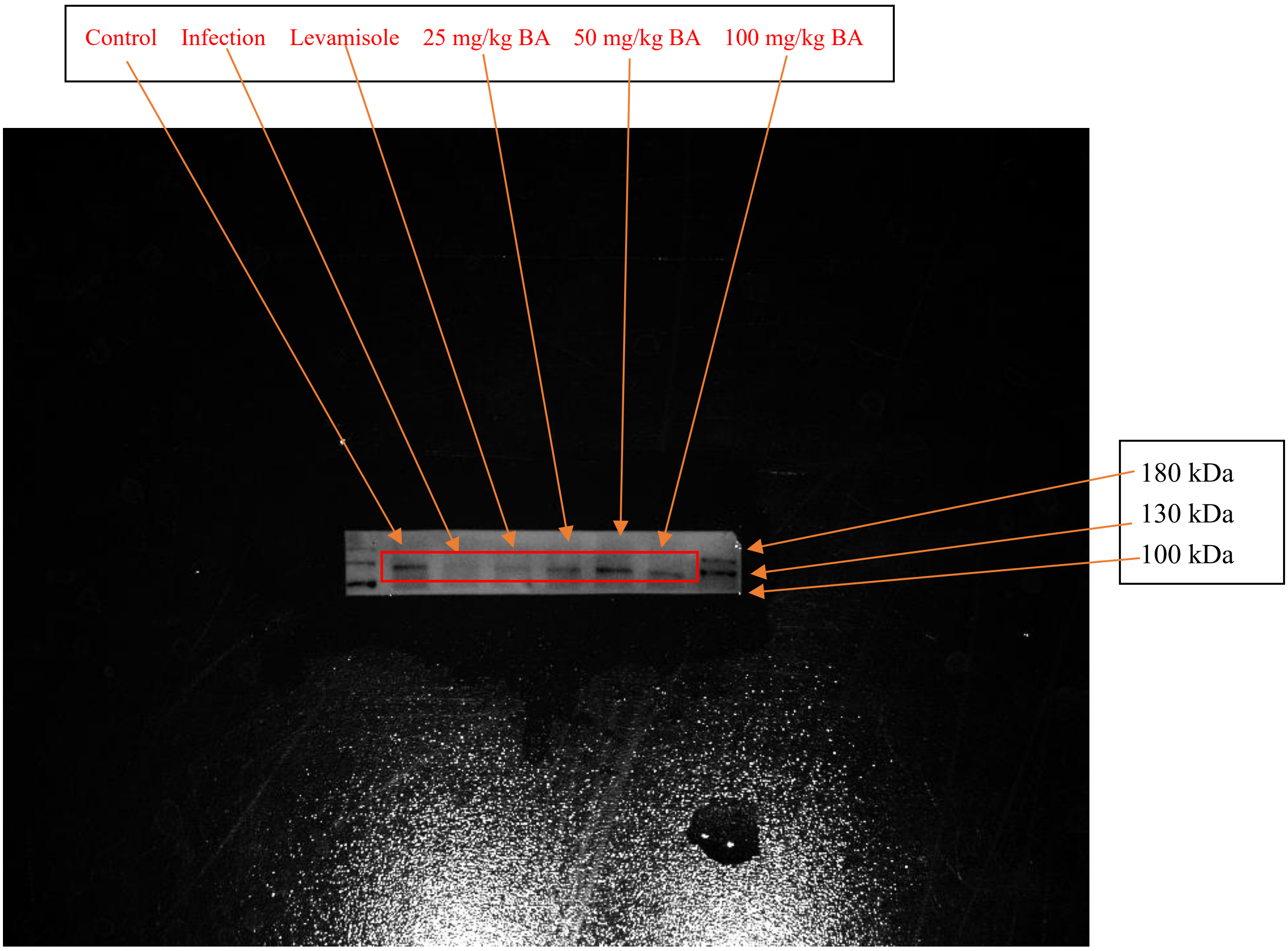

Fig 5

Fig 5. B:

Notch1 (repeat 2)

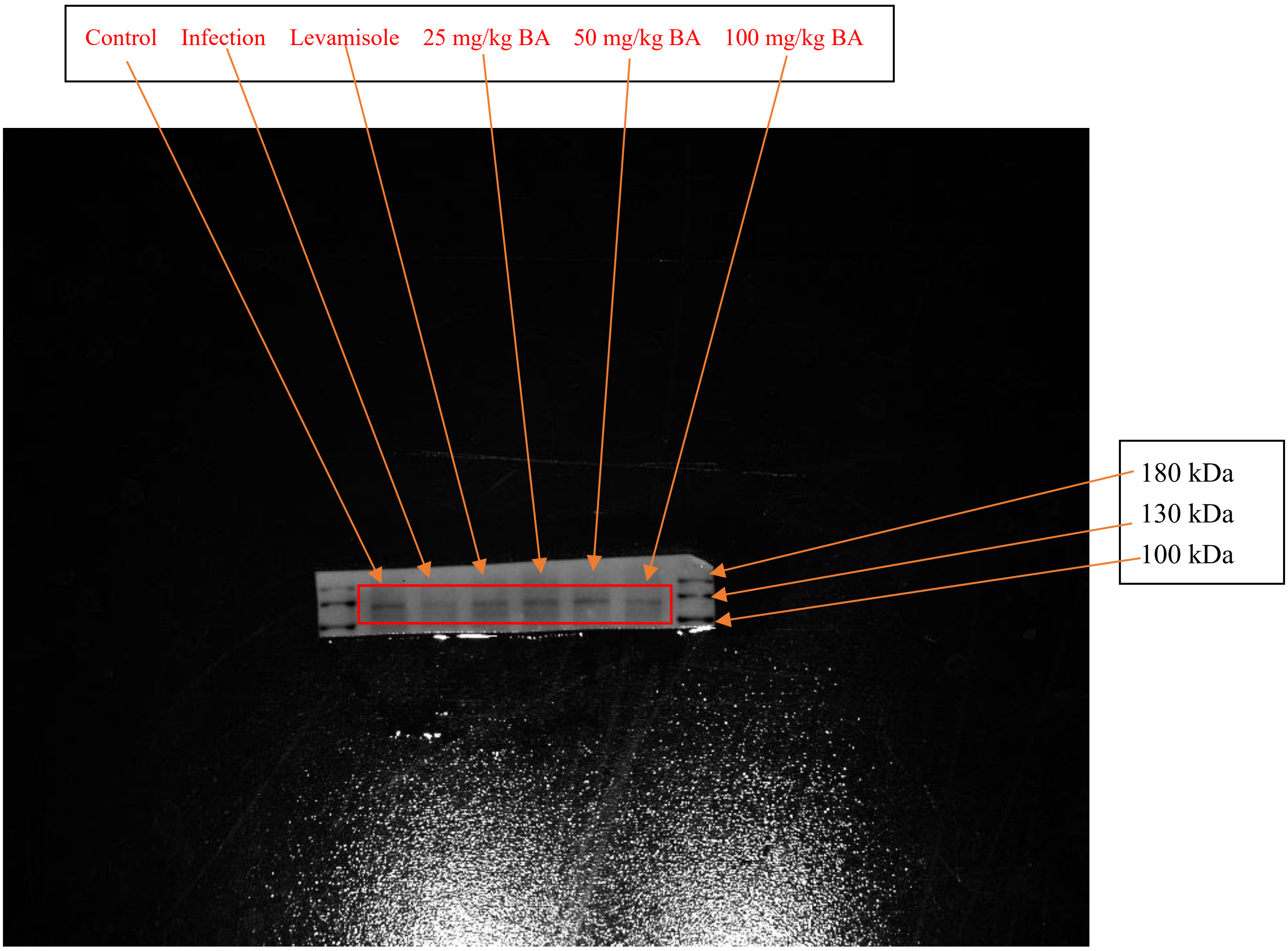

Fig 5

Fig 5. B:

Notch1 (repeat 2)

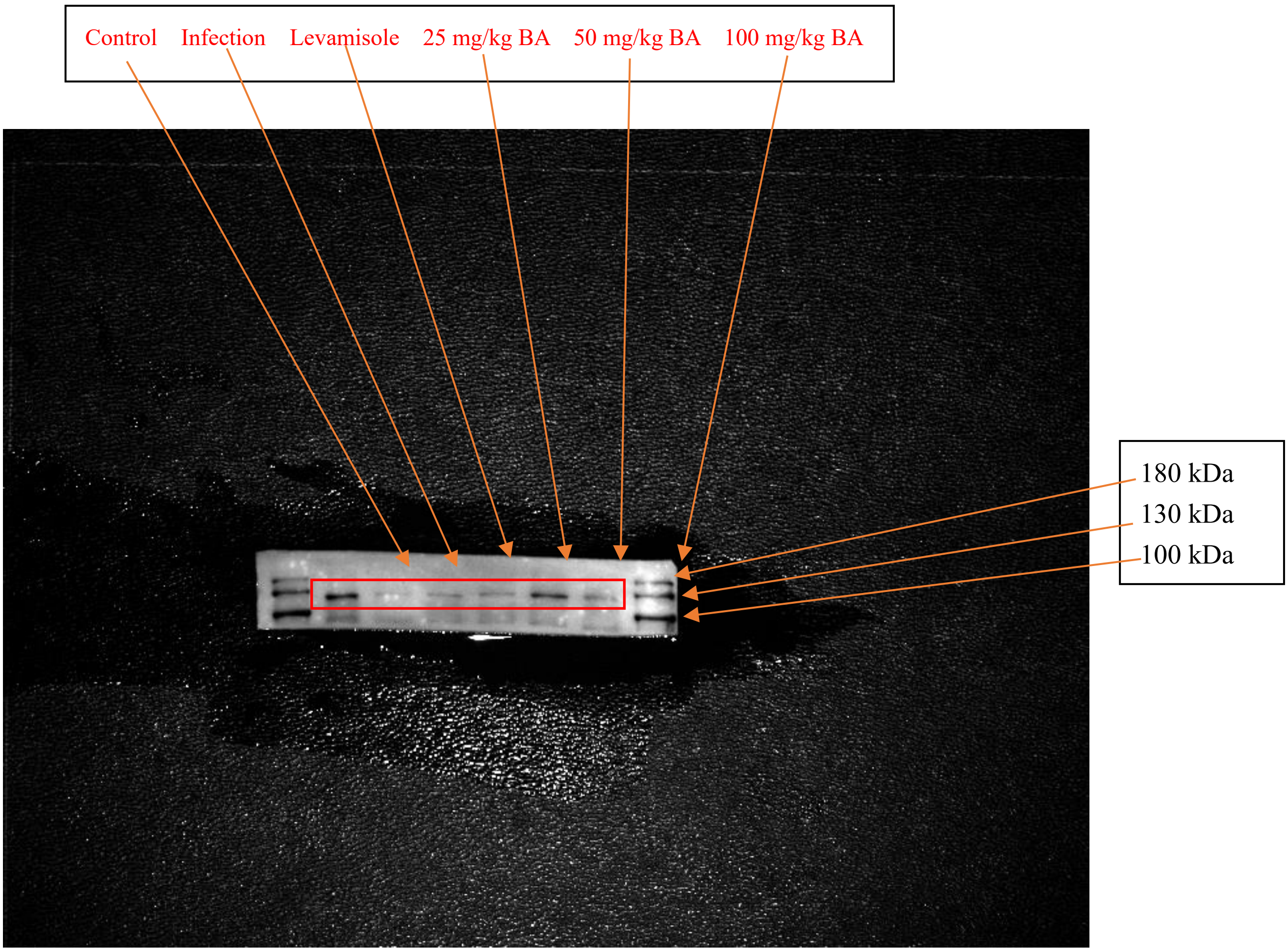

Fig 5

Fig 5. D:

GAPDH (repeat 1)

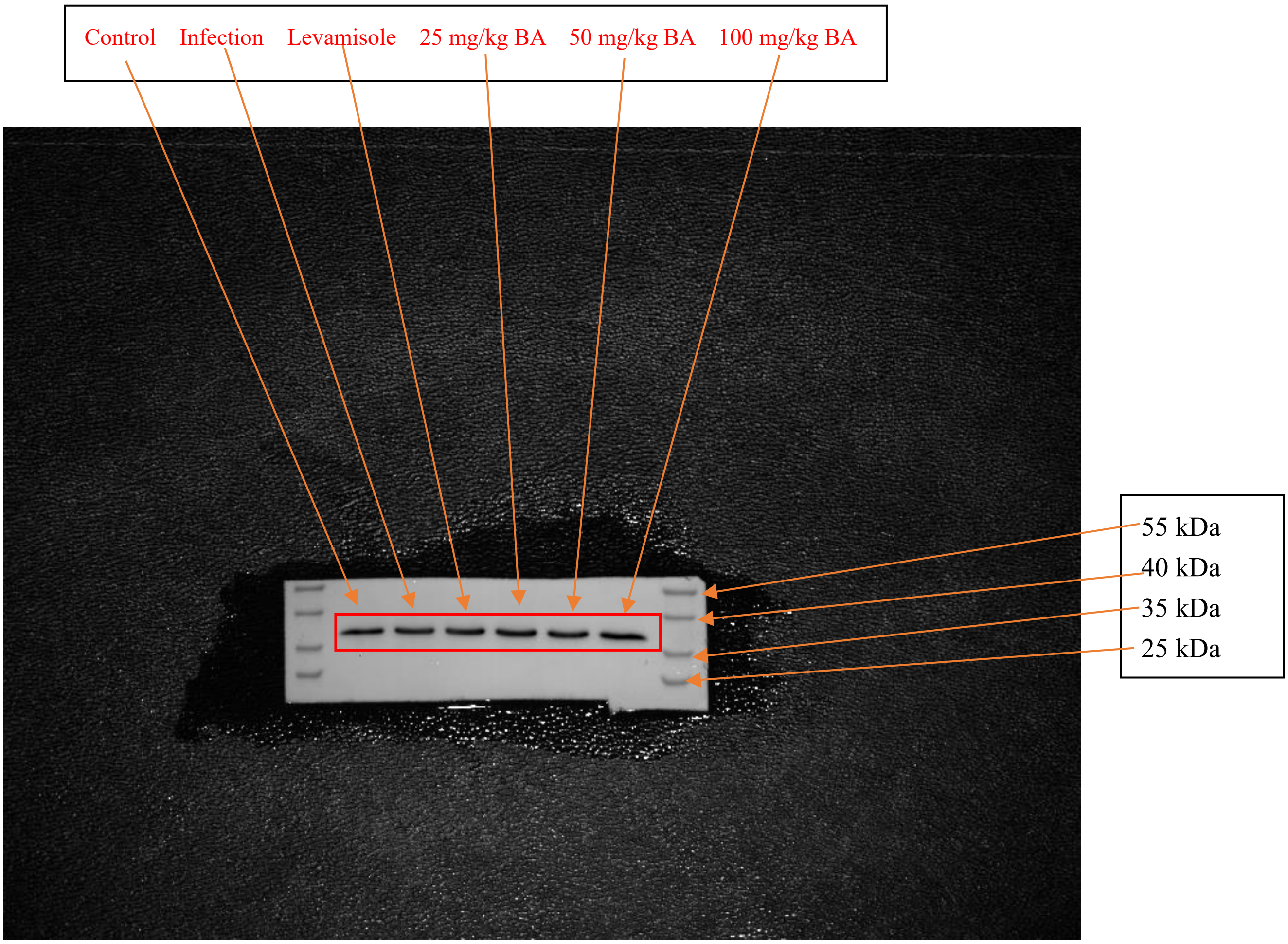

Fig 5

Fig 5. D:

GAPDH (repeat 2)

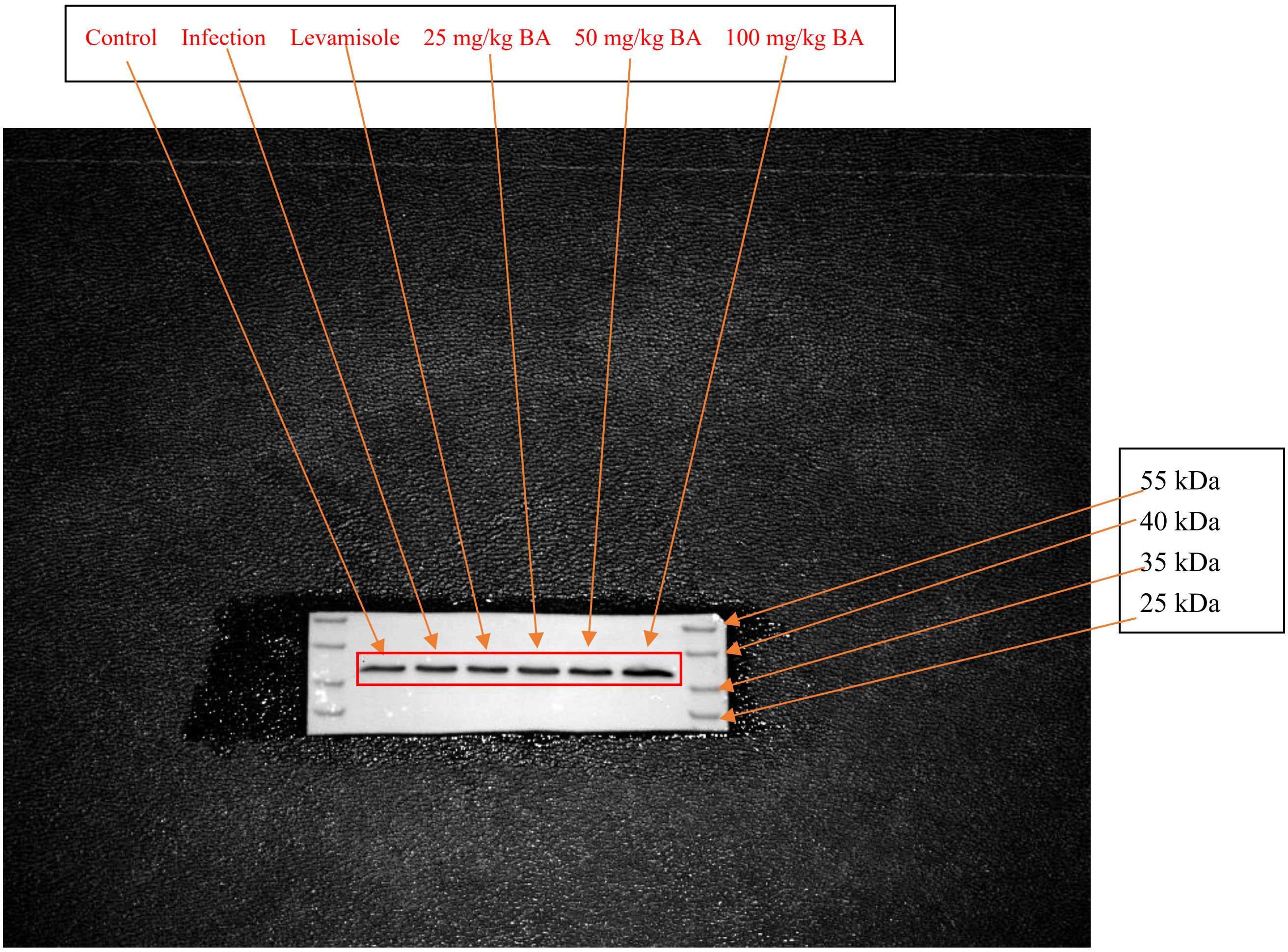

Fig 5

Fig 5. D:

GAPDH (repeat 3)

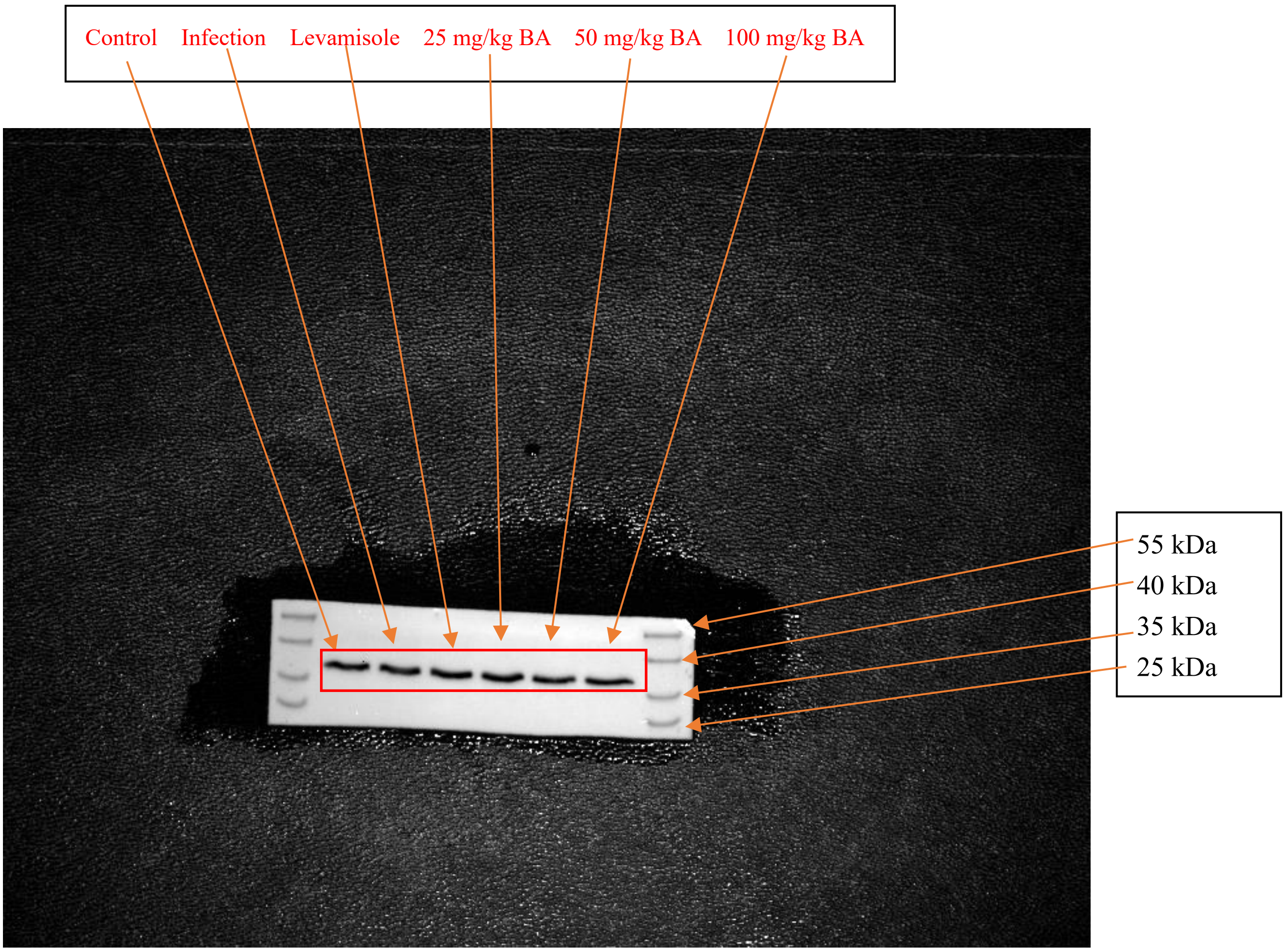

Fig 5

Fig 5. D:

WNT3A (repeat 1)

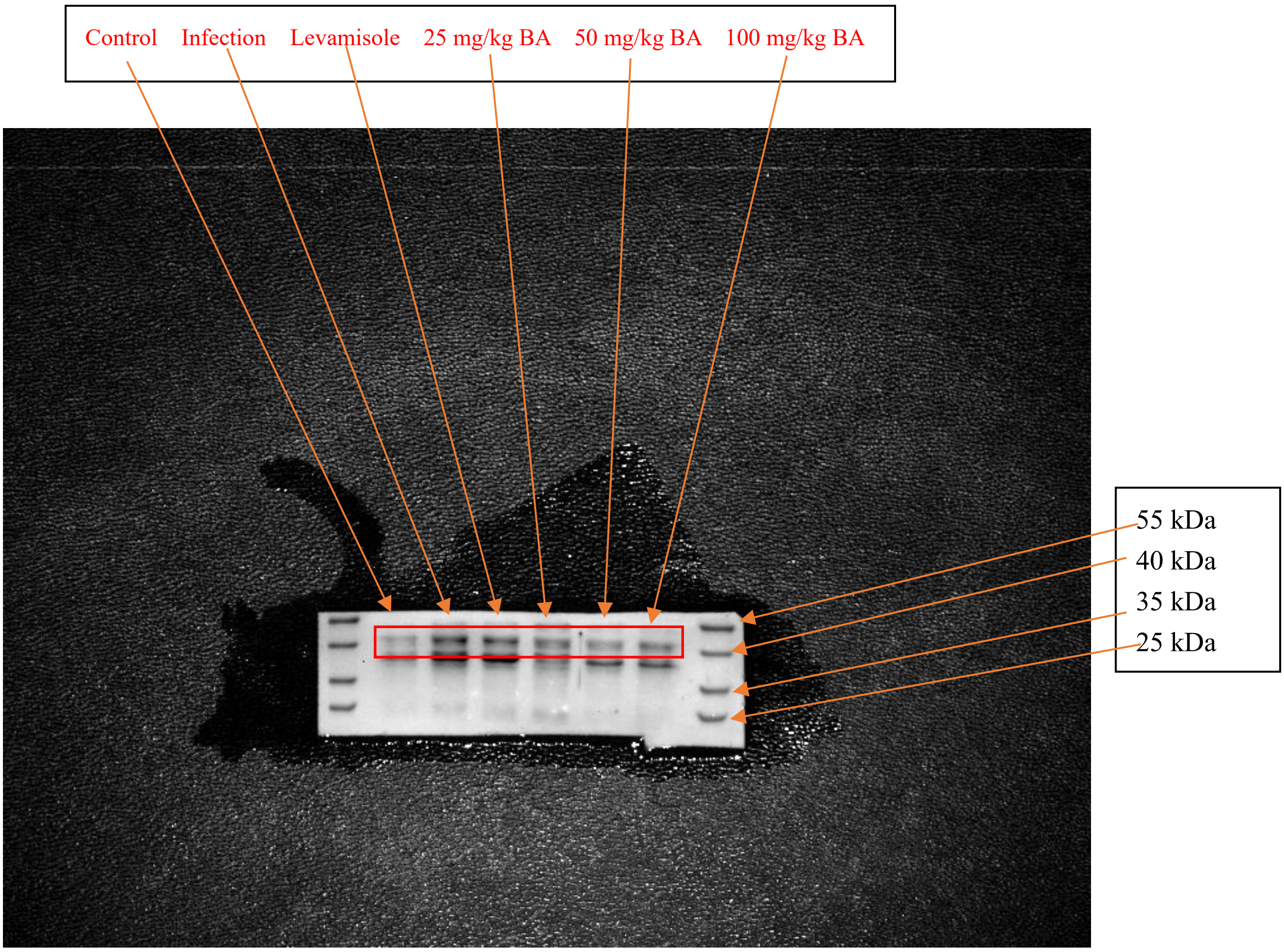

Fig 5

Fig 5. D:

WNT3A (repeat 2)

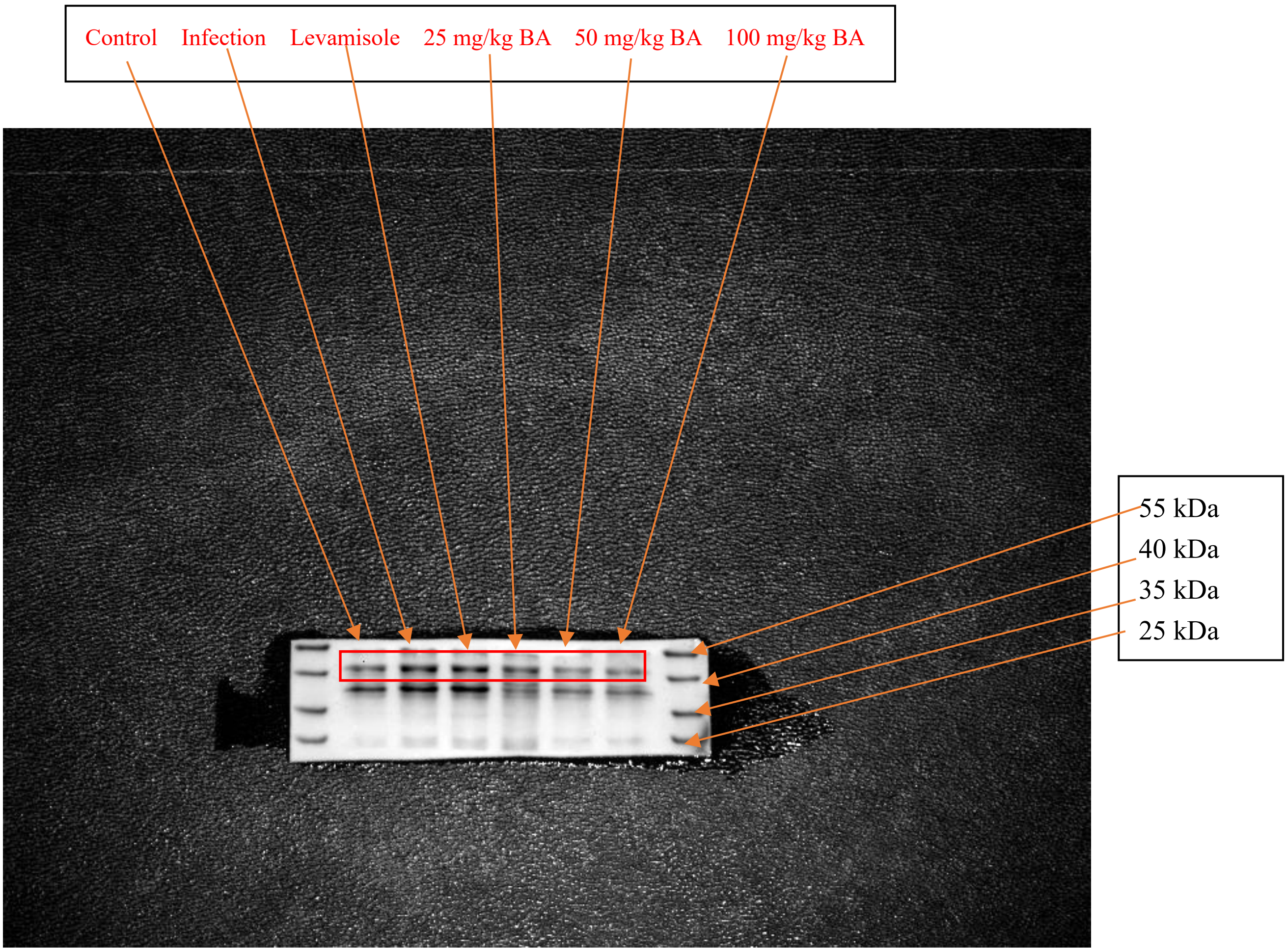

Fig 5

Fig 5. D:

WNT3A (repeat 3)

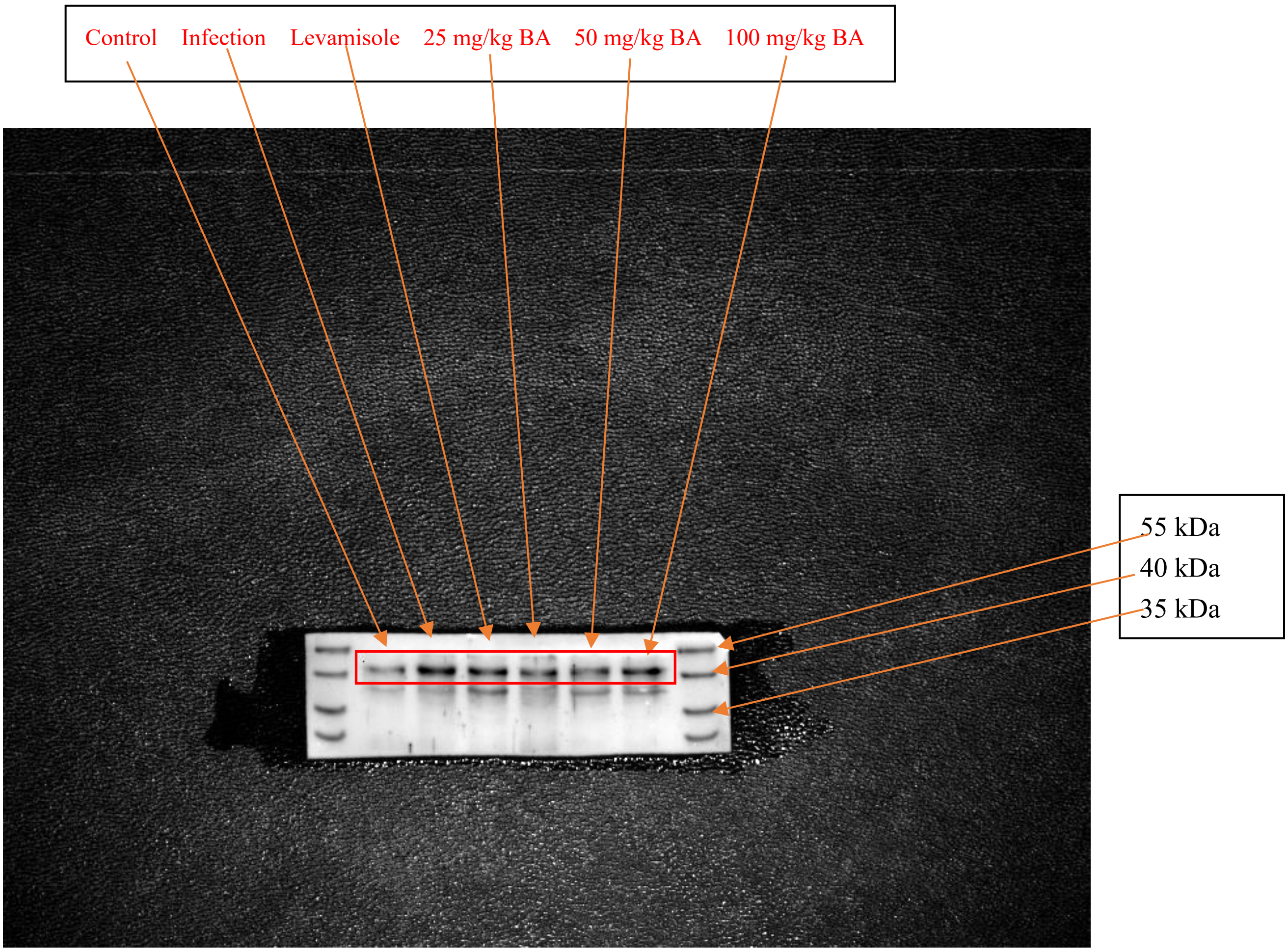

Fig 5

Fig 5. F:

GAPDH (repeat 1)

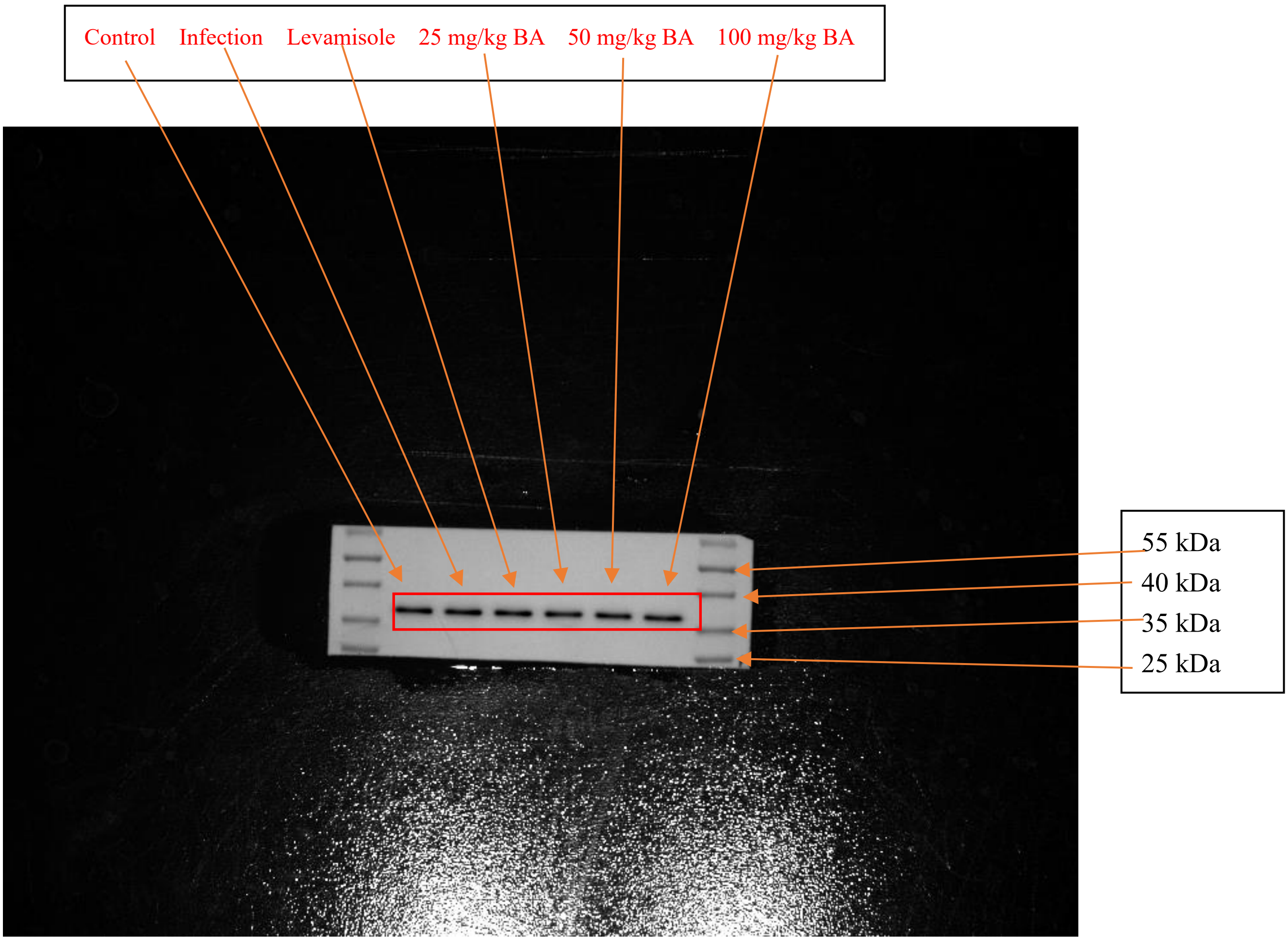

Fig 5

Fig 5. F:

GAPDH (repeat 2)

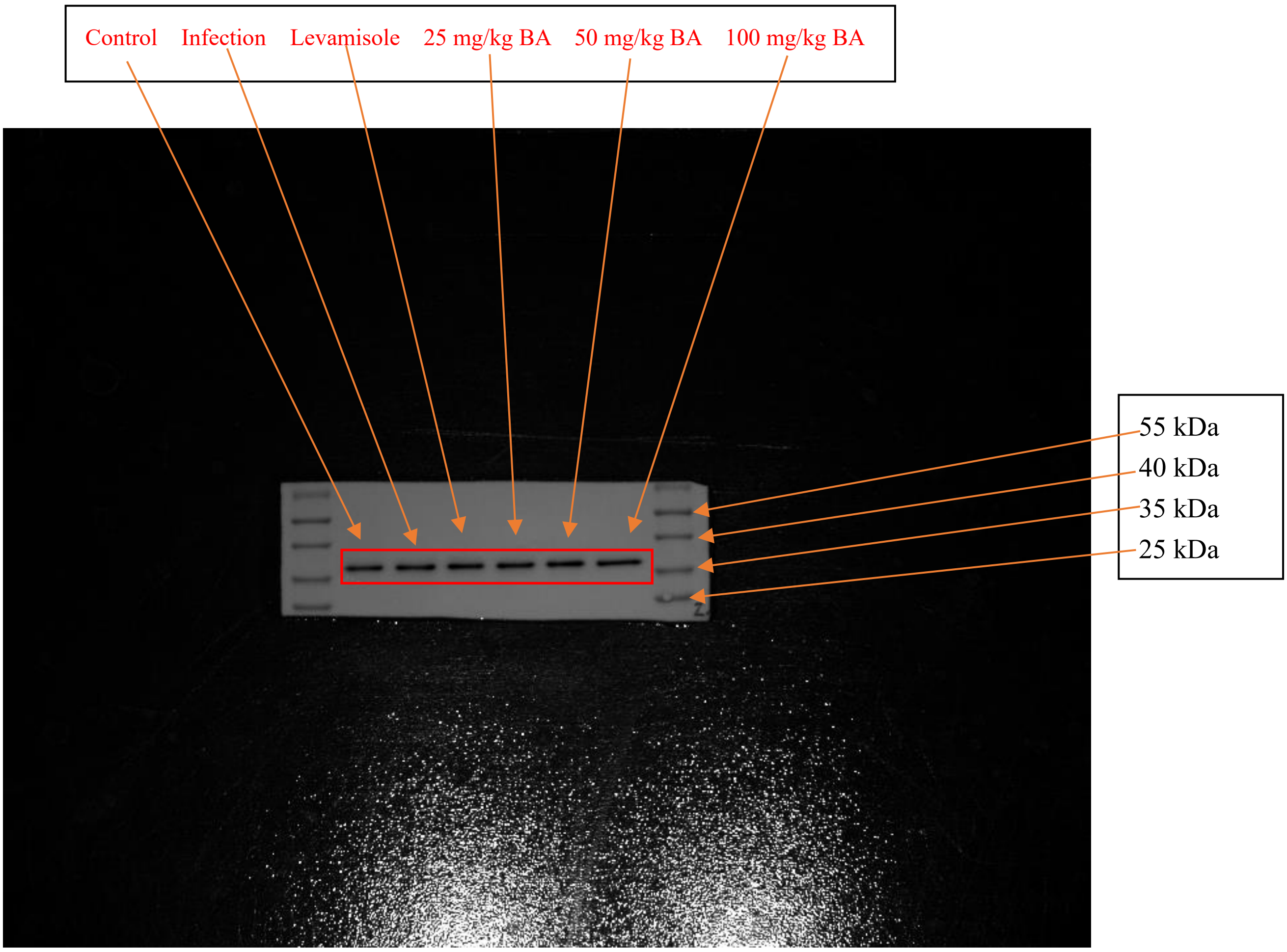

Fig 5

Fig 5. F:
GAPDH (repeat 3)

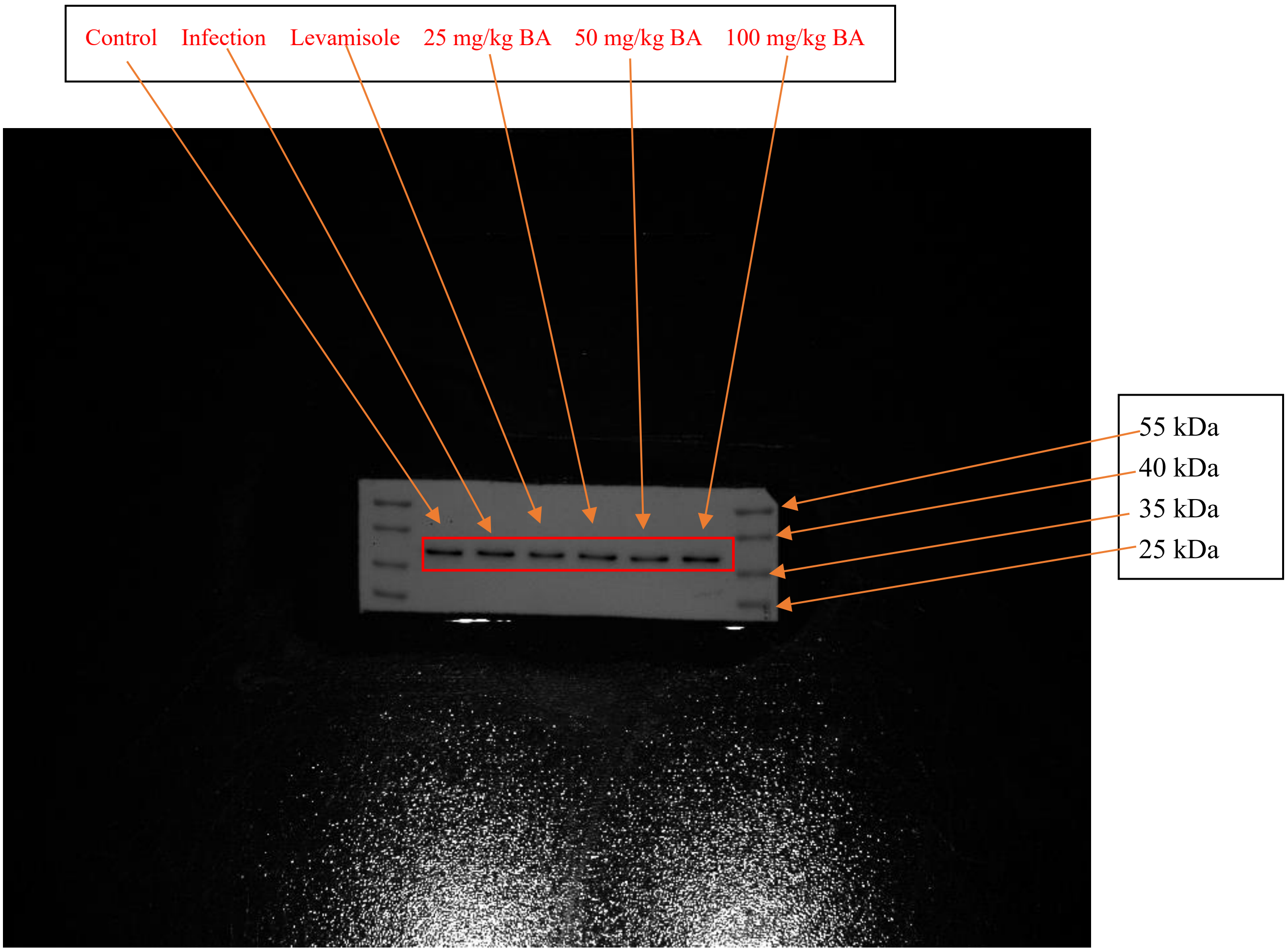

Fig 5

Fig 5. F:

RBP-J (repeat 1)

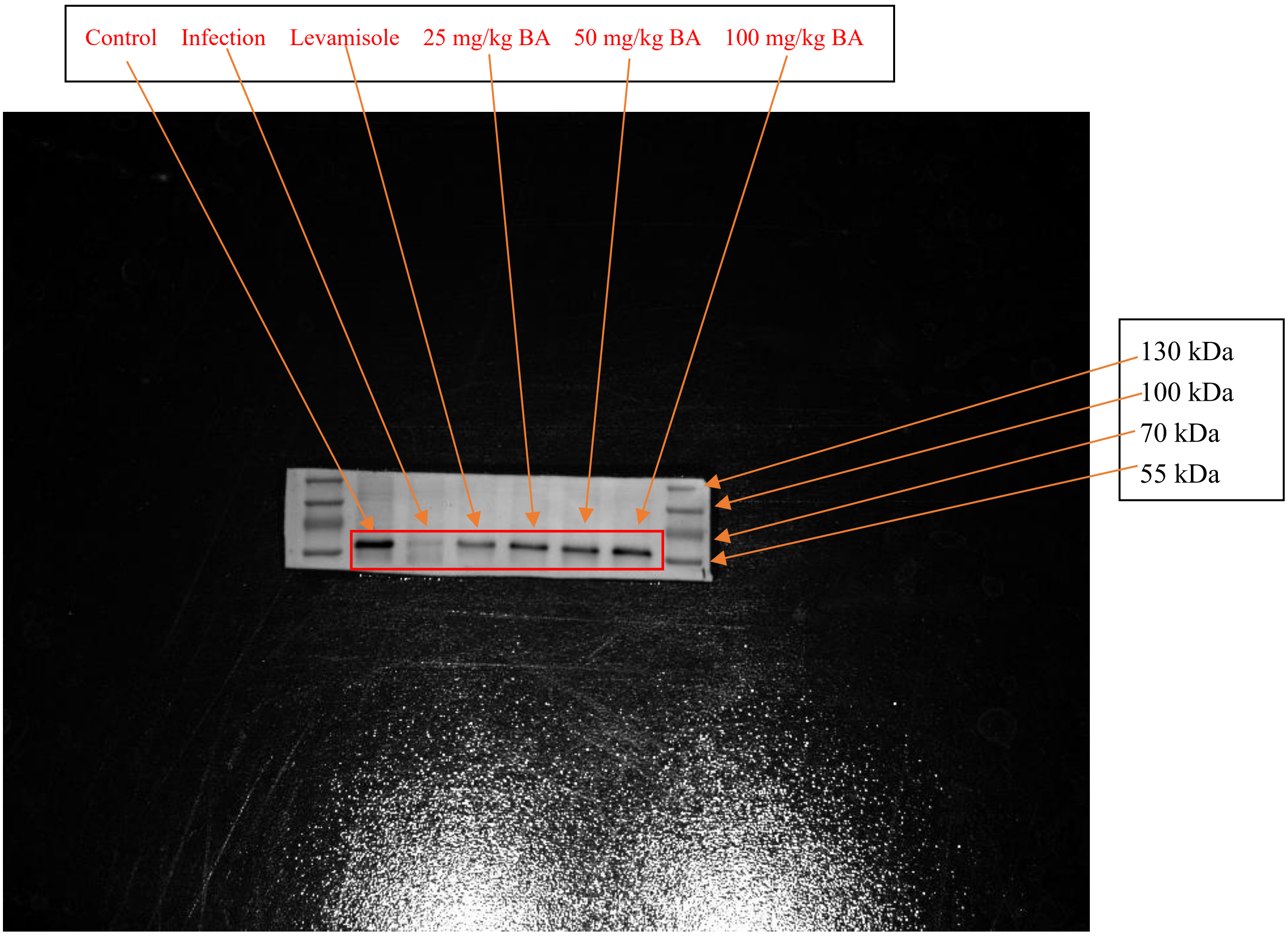

Fig 5

Fig 5. F:

RBP-J (repeat 2)

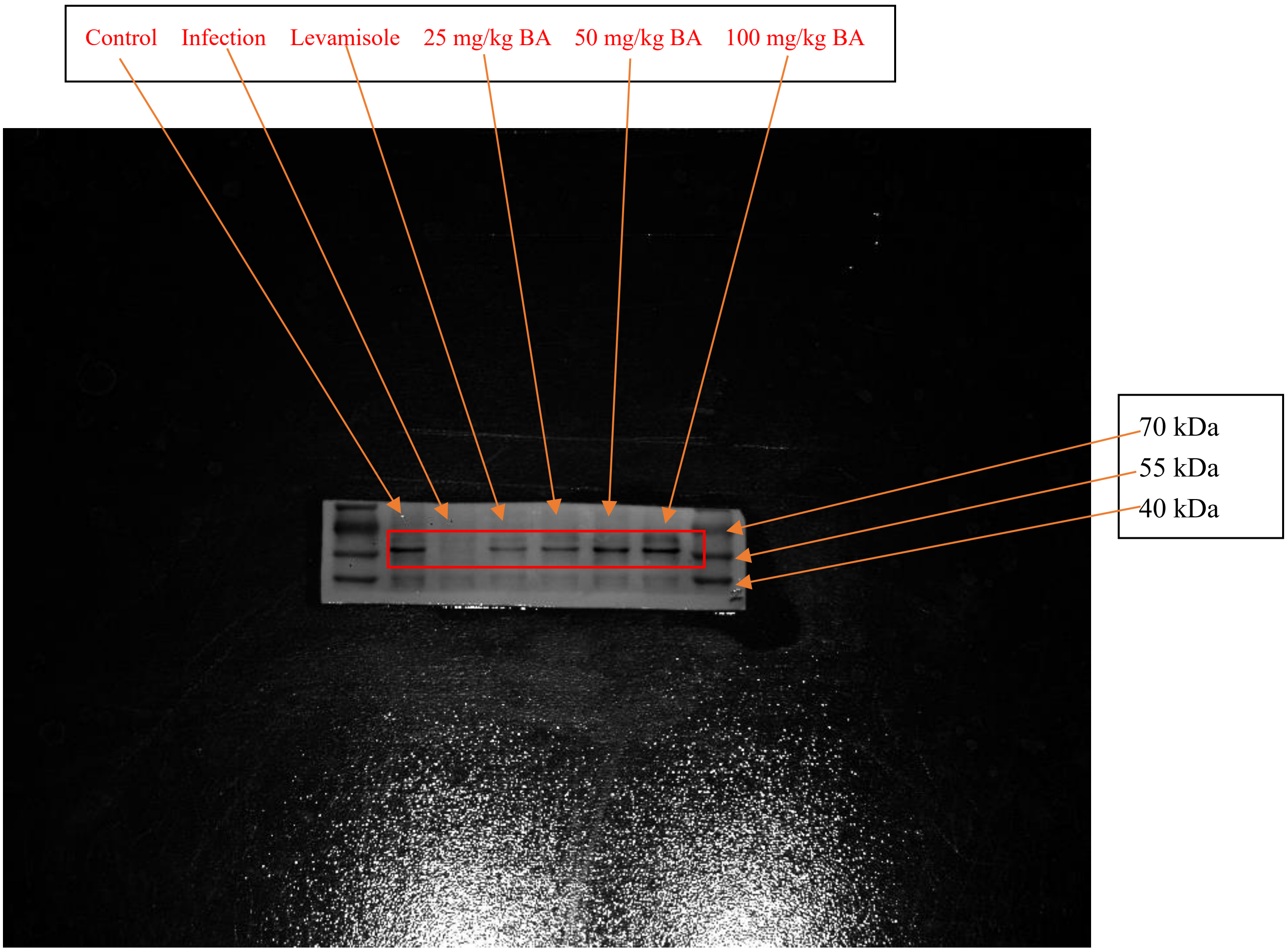

Fig 5

Fig 5. F:

RBP-J (repeat 3)

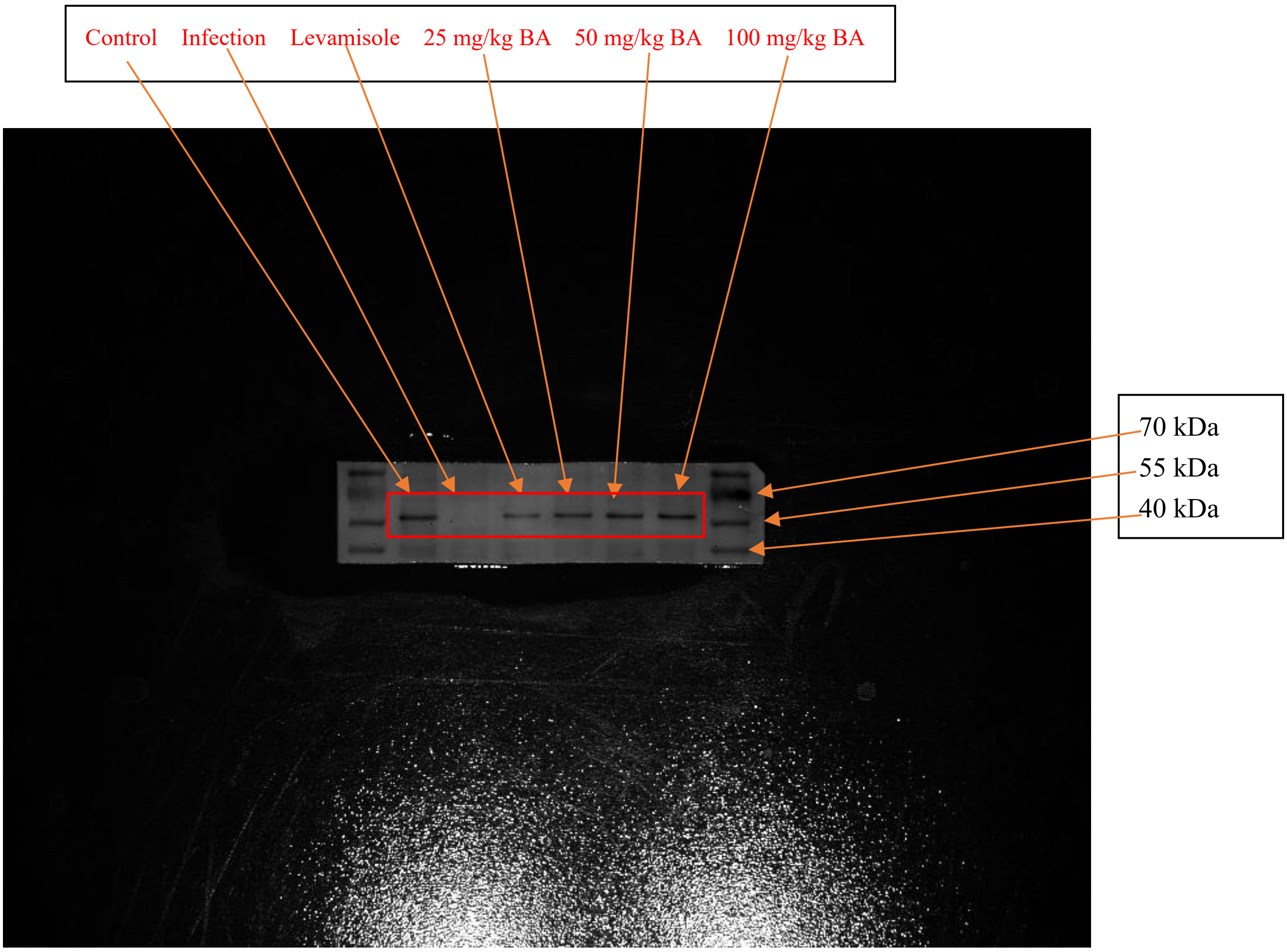

Fig 5

Fig 5. H:

GAPDH (repeat 1)

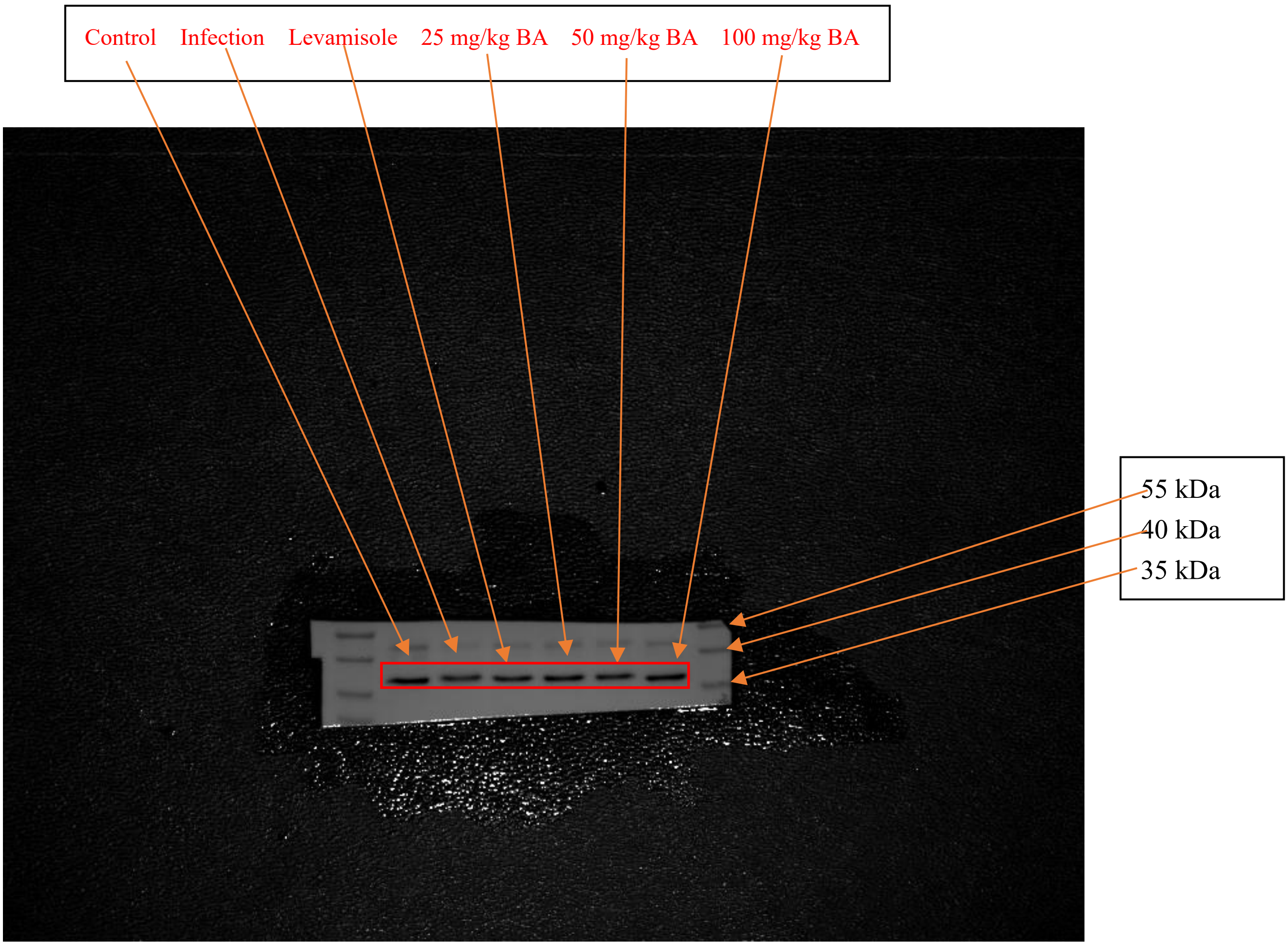

Fig 5

Fig 5. H:

GAPDH (repeat 2)

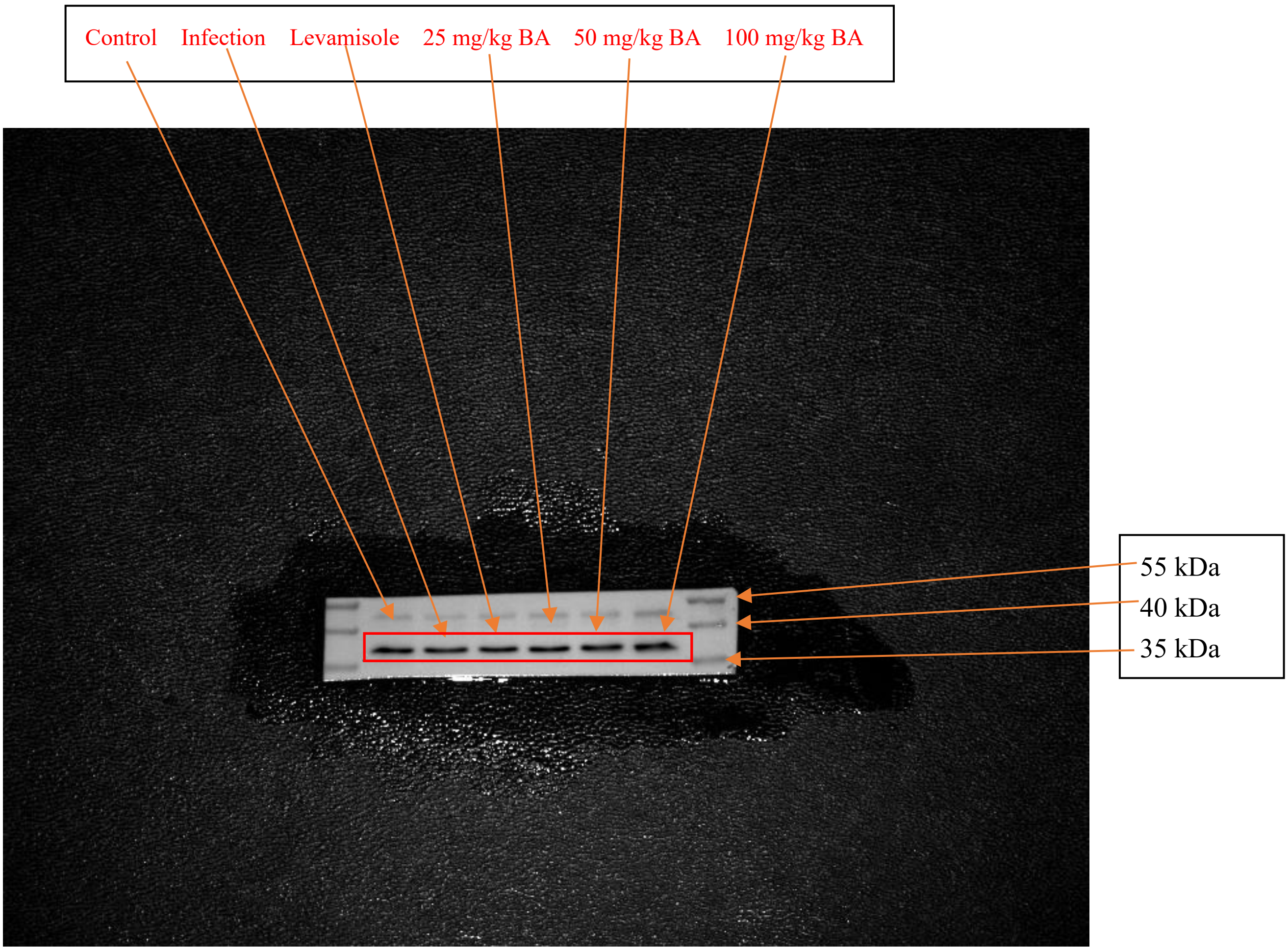

Fig 5

Fig 5. H:
GAPDH (repeat 3)

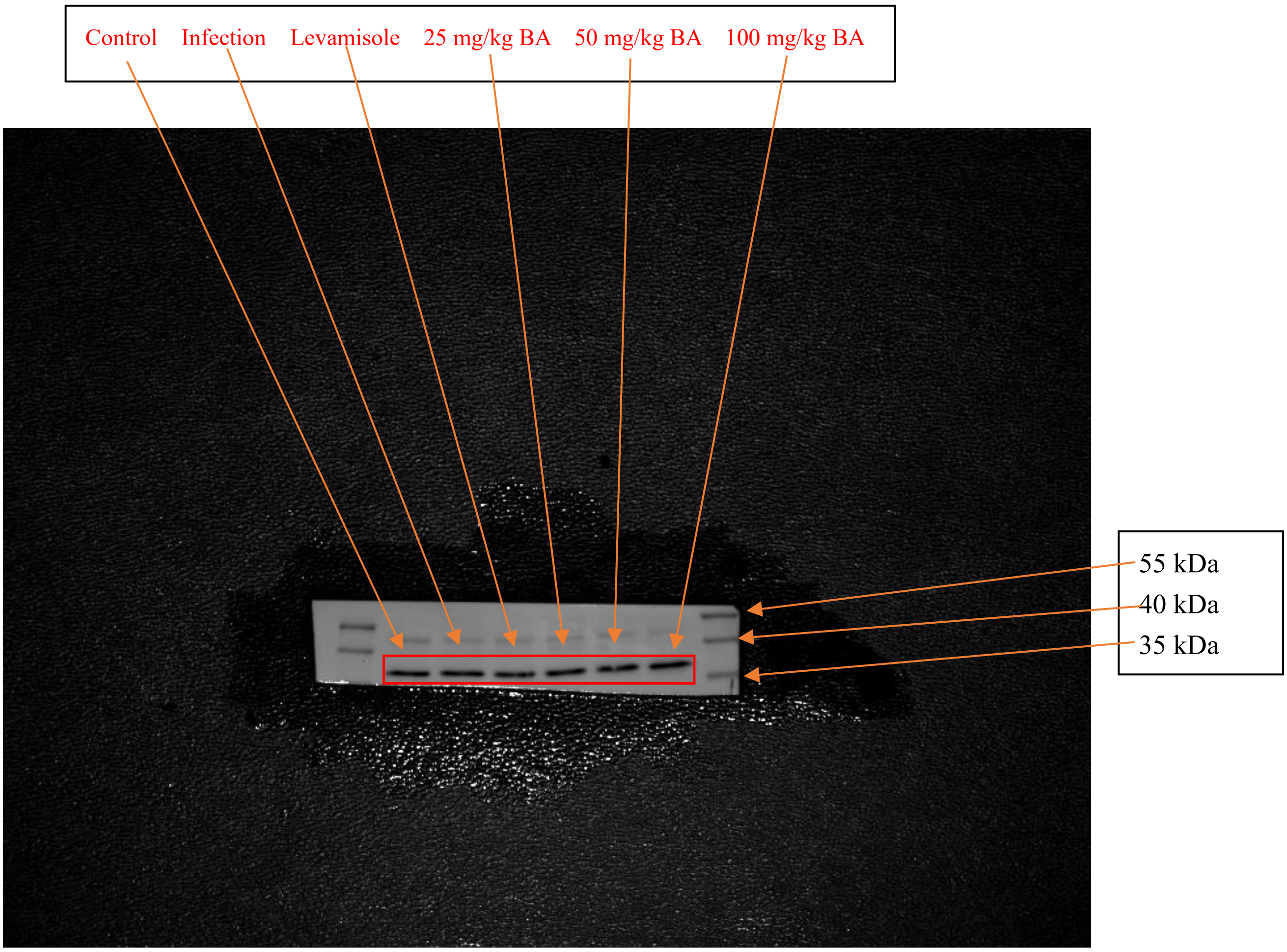

Fig 5

Fig 5. H:
GSK3β (repeat 1)

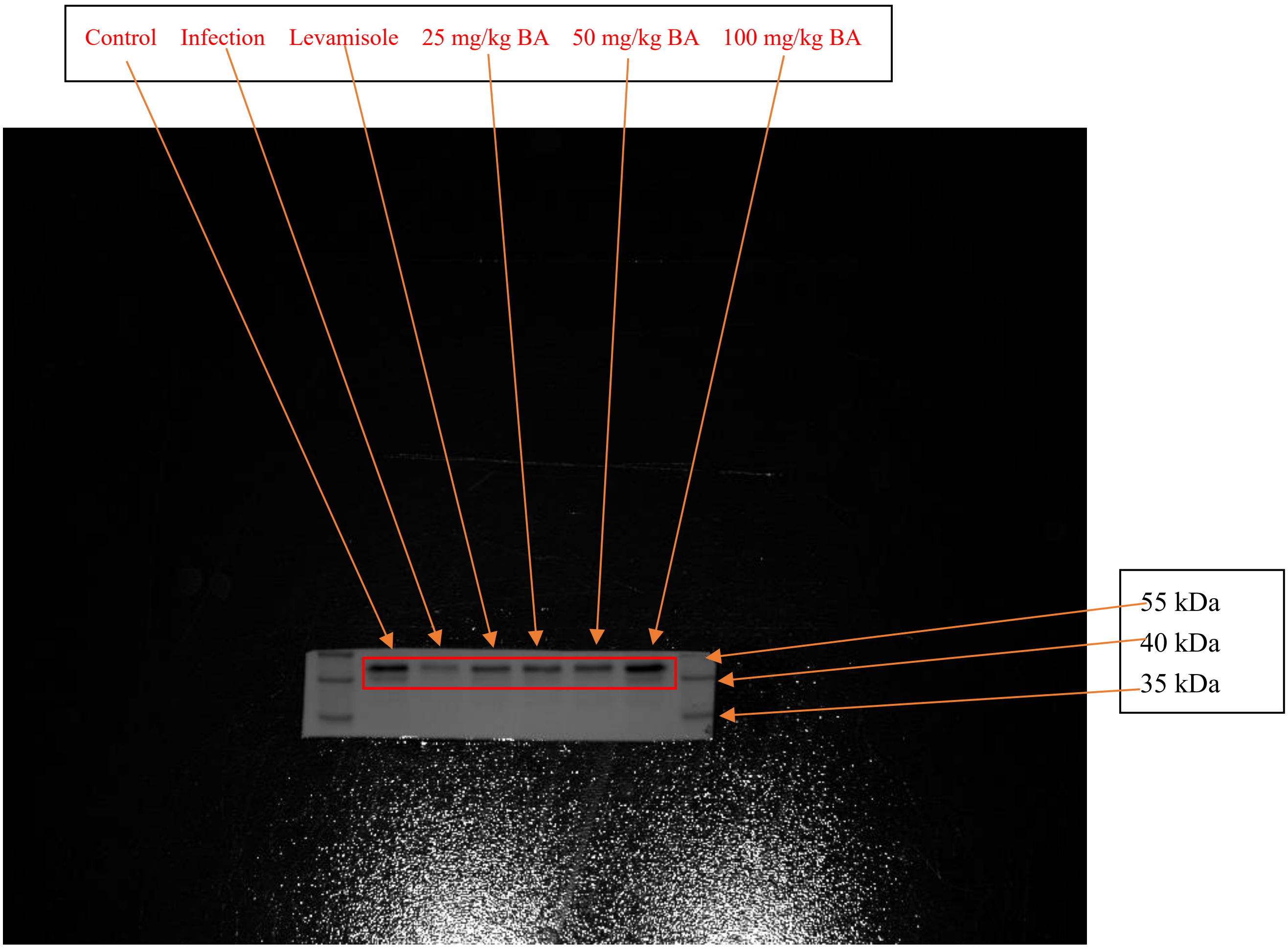

Fig 5

Fig 5. H:

GSK-3β (repeat 2)

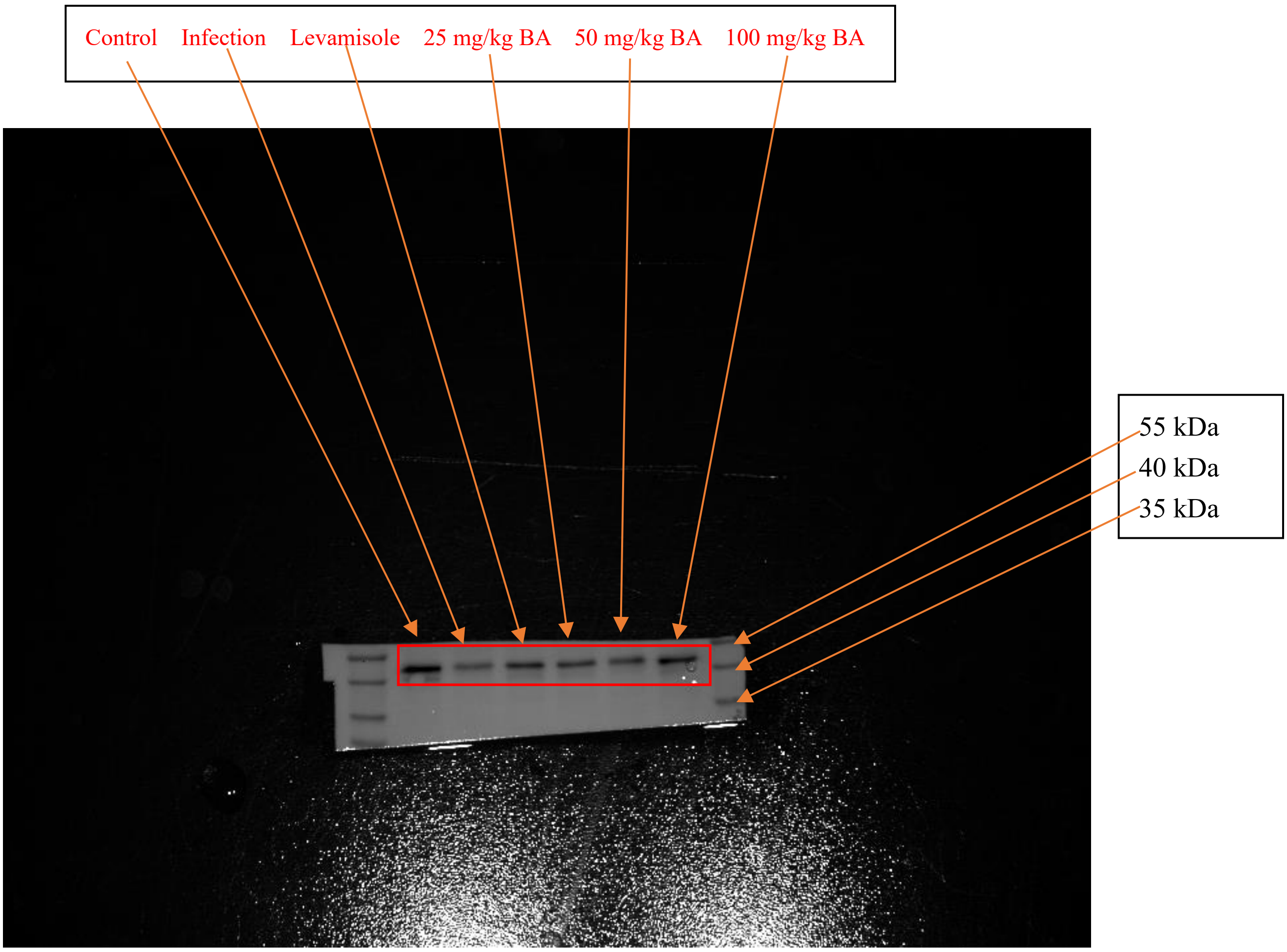

Fig 5

Fig 5. H:

GSK-3β (repeat 3)

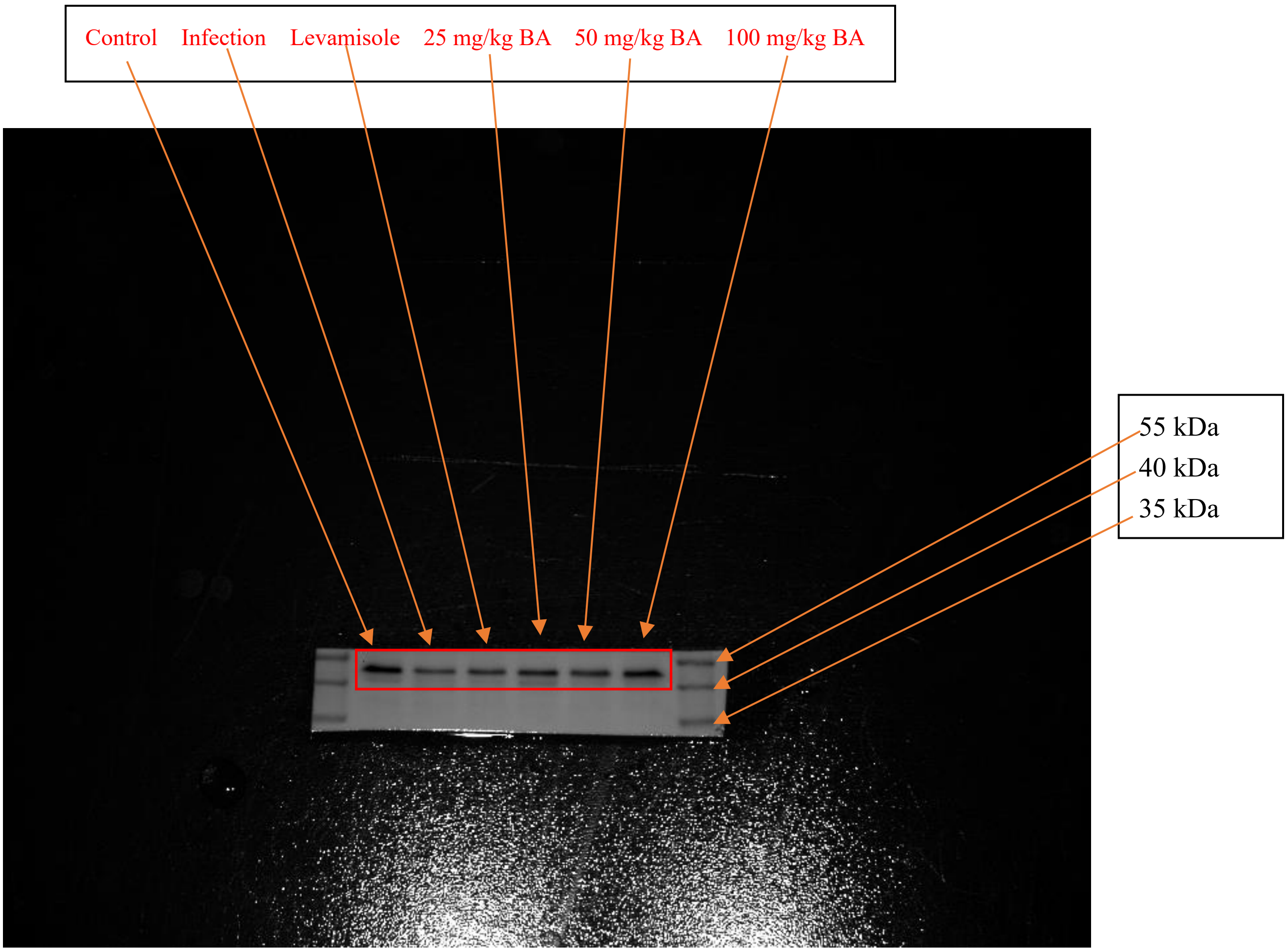

Fig 5

Fig 5. J:

GAPDH (repeat 1)

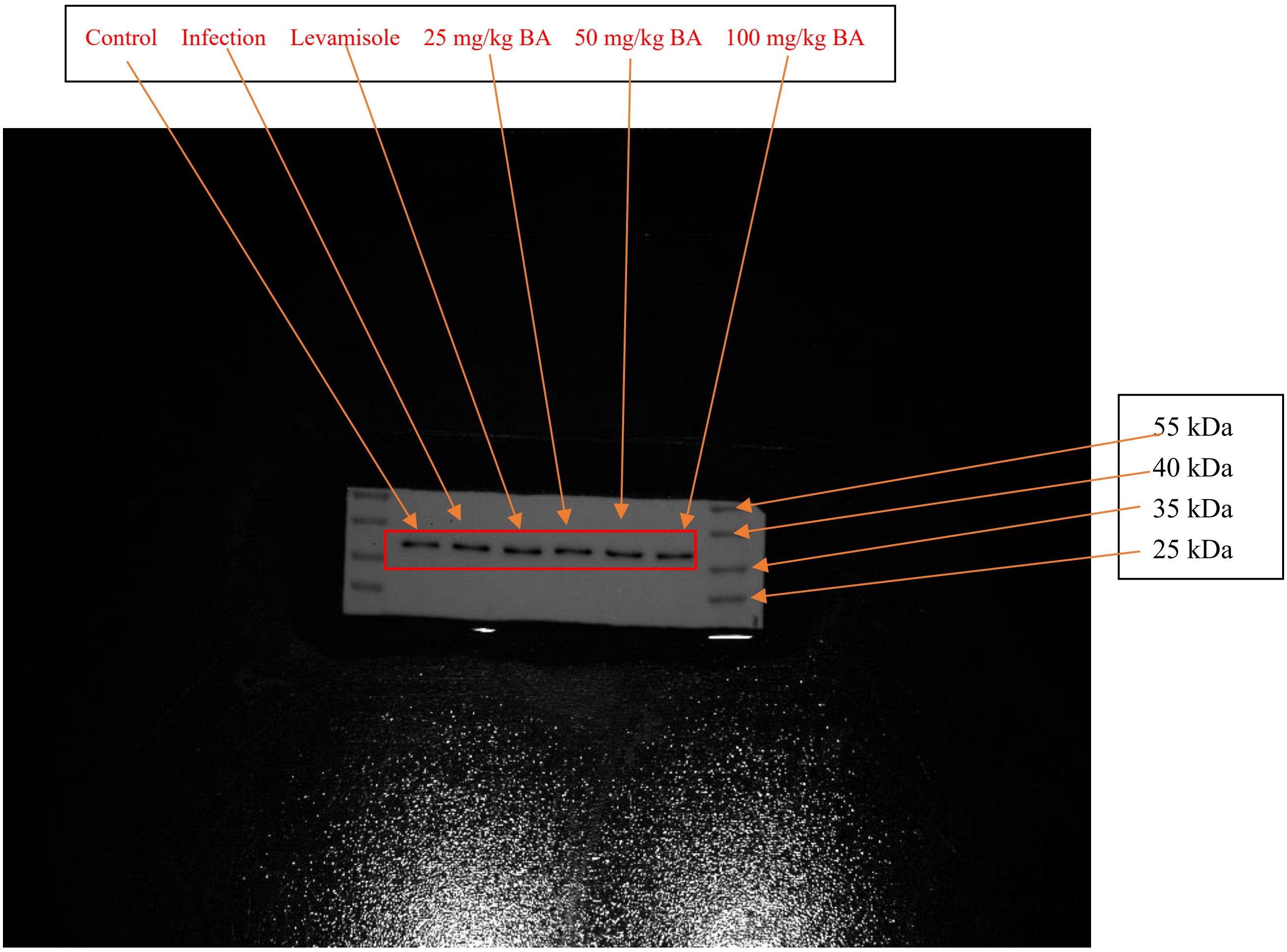

Fig 5

Fig 5. J:
GAPDH (repeat 2)

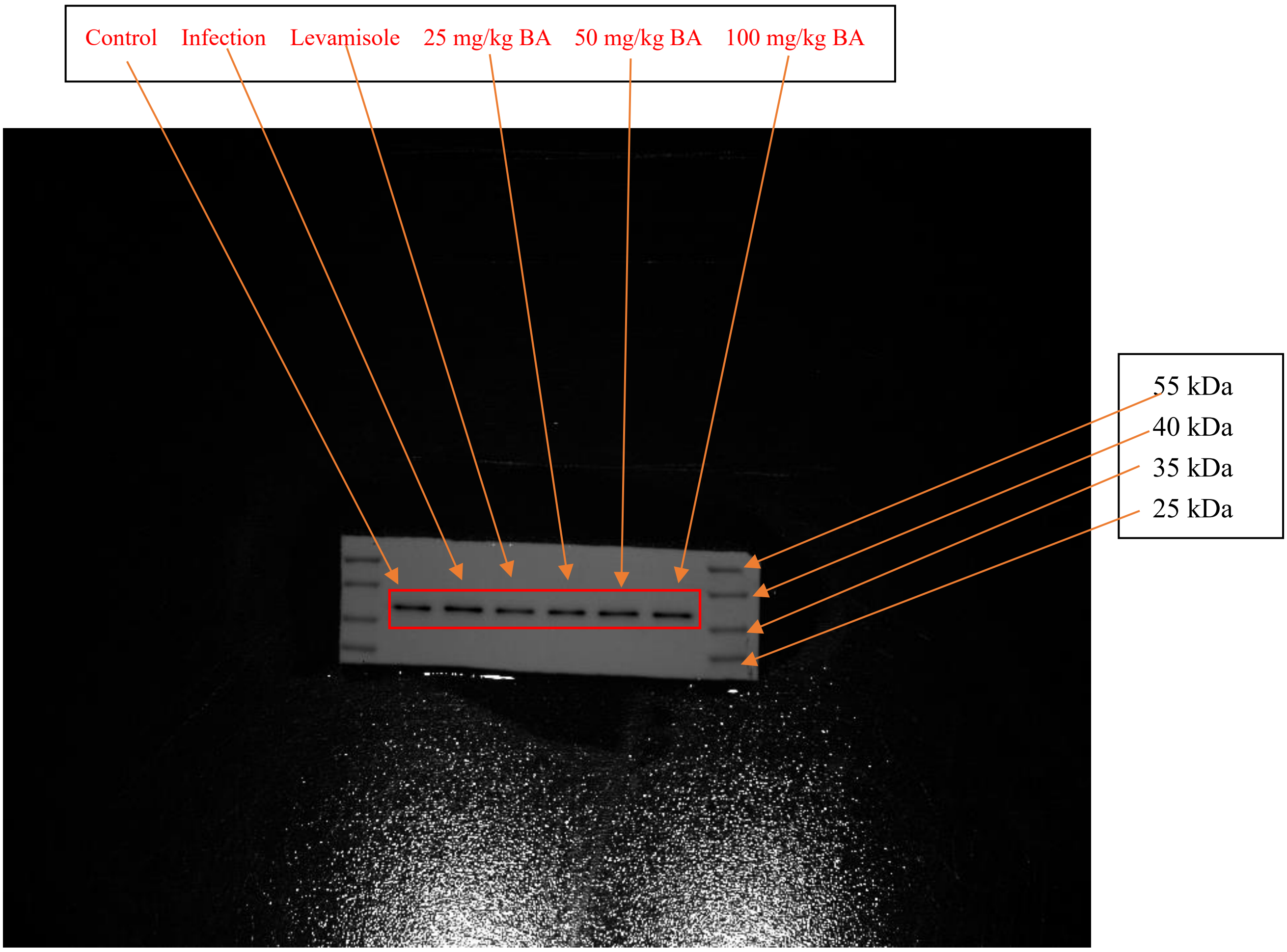

Fig 5

Fig 5. J:

GAPDH (repeat 3)

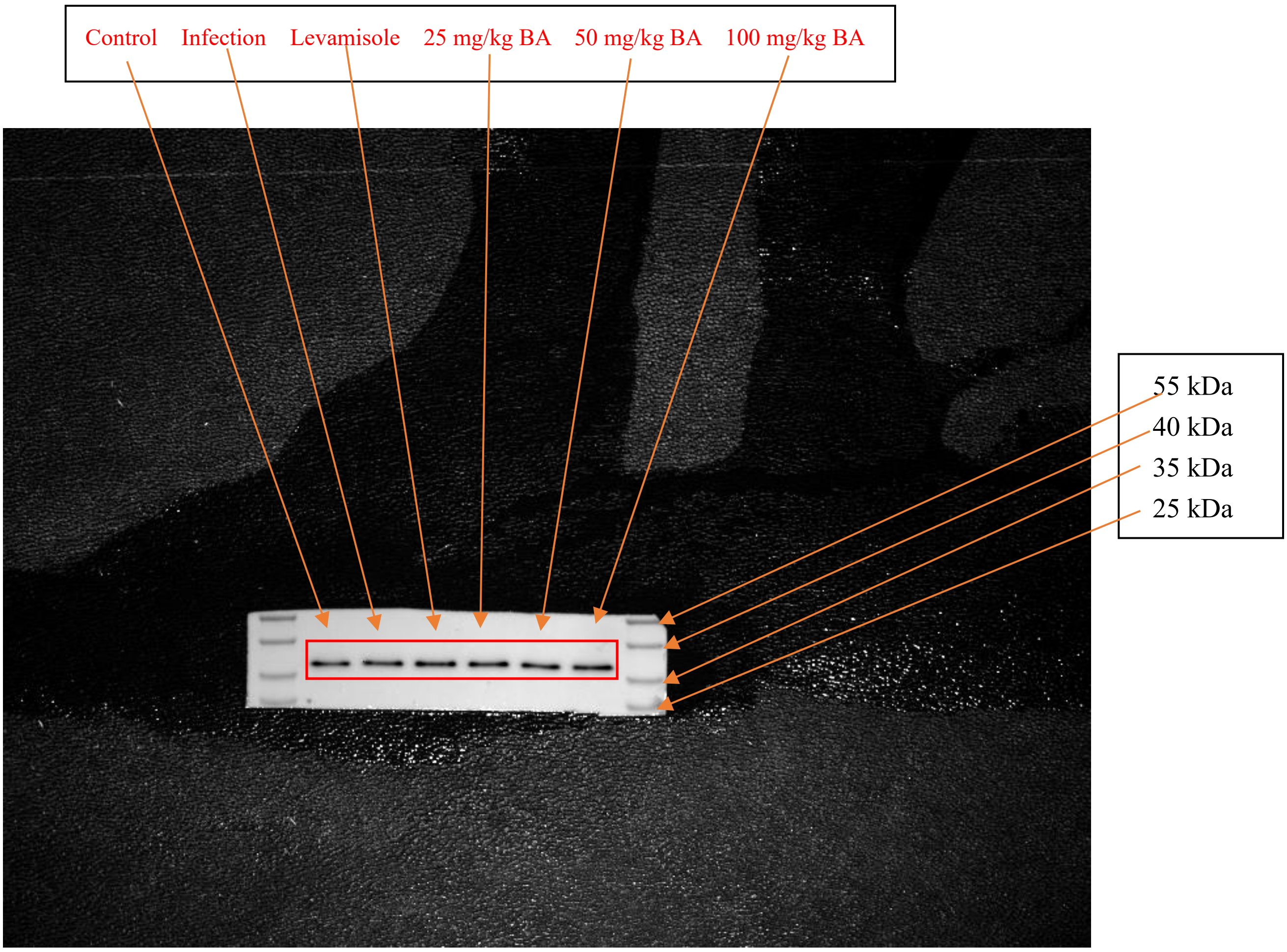

Fig 5

Fig 5. J:

JAG1 (repeat 1)

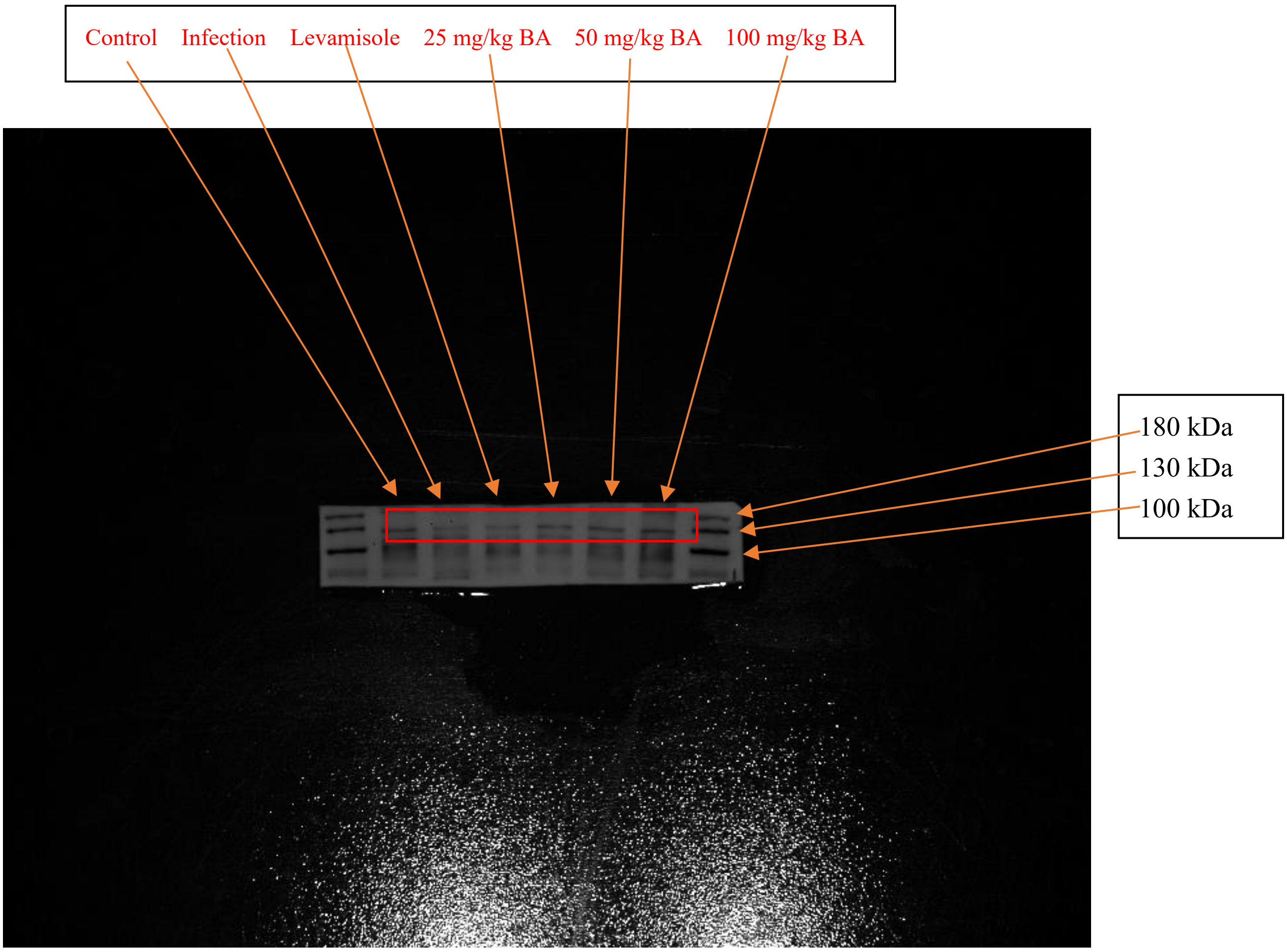

Fig 5

Fig 5. J:

JAG1 (repeat 2)

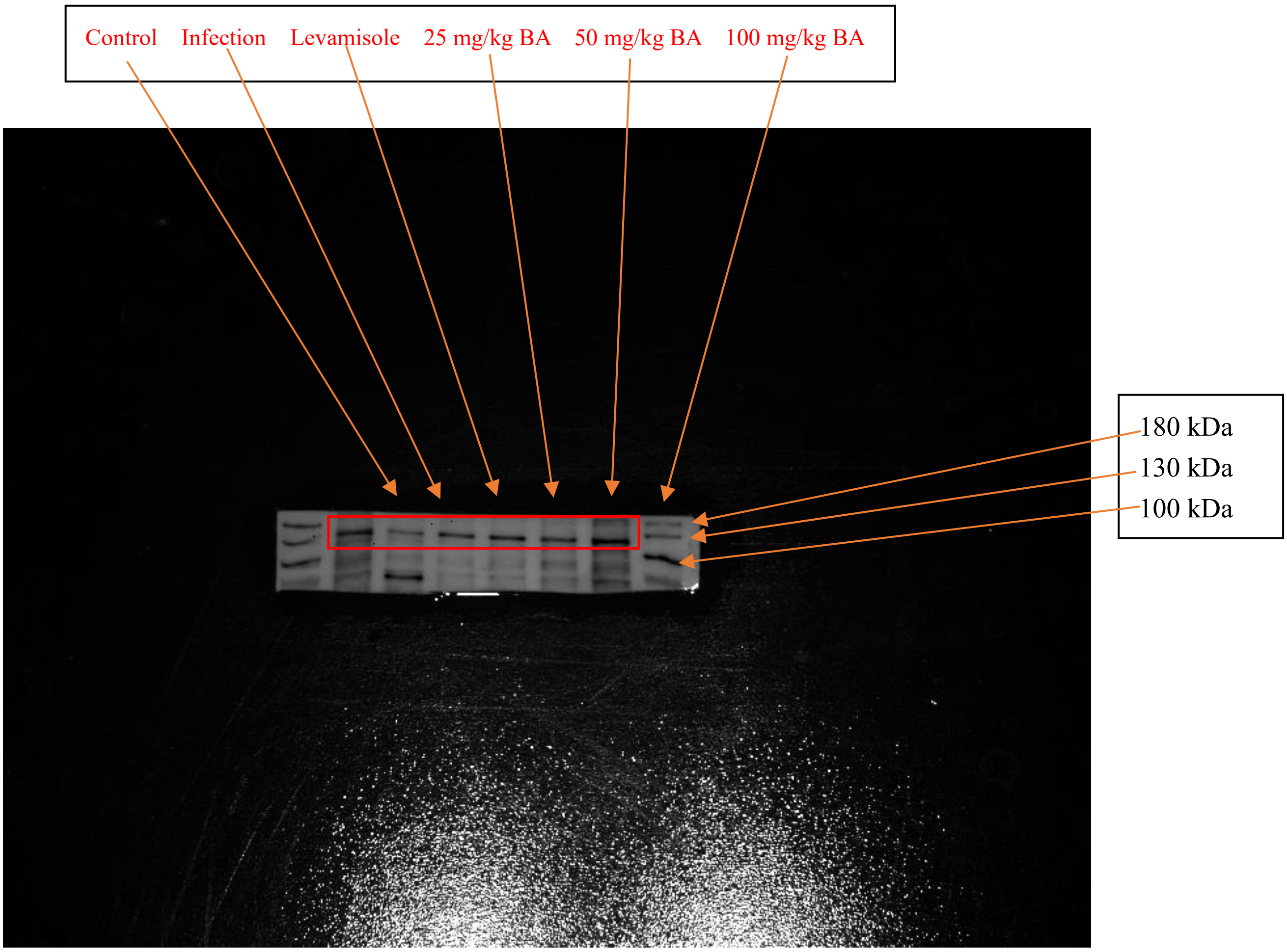

Fig 5

Fig 5. J:

JAG1 (repeat 3)

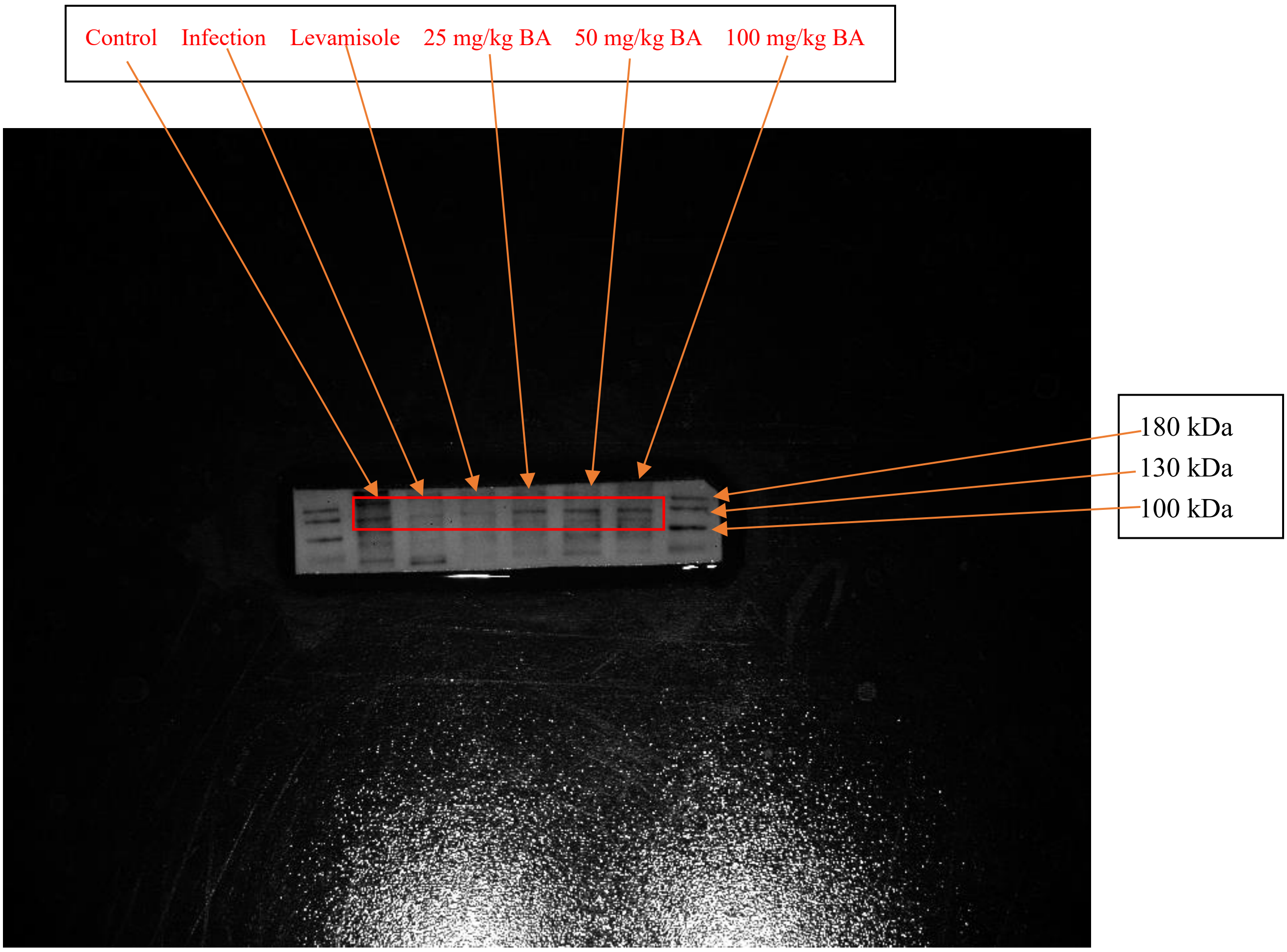

Fig 5

Fig 5. L:

GAPDH (repeat 1)

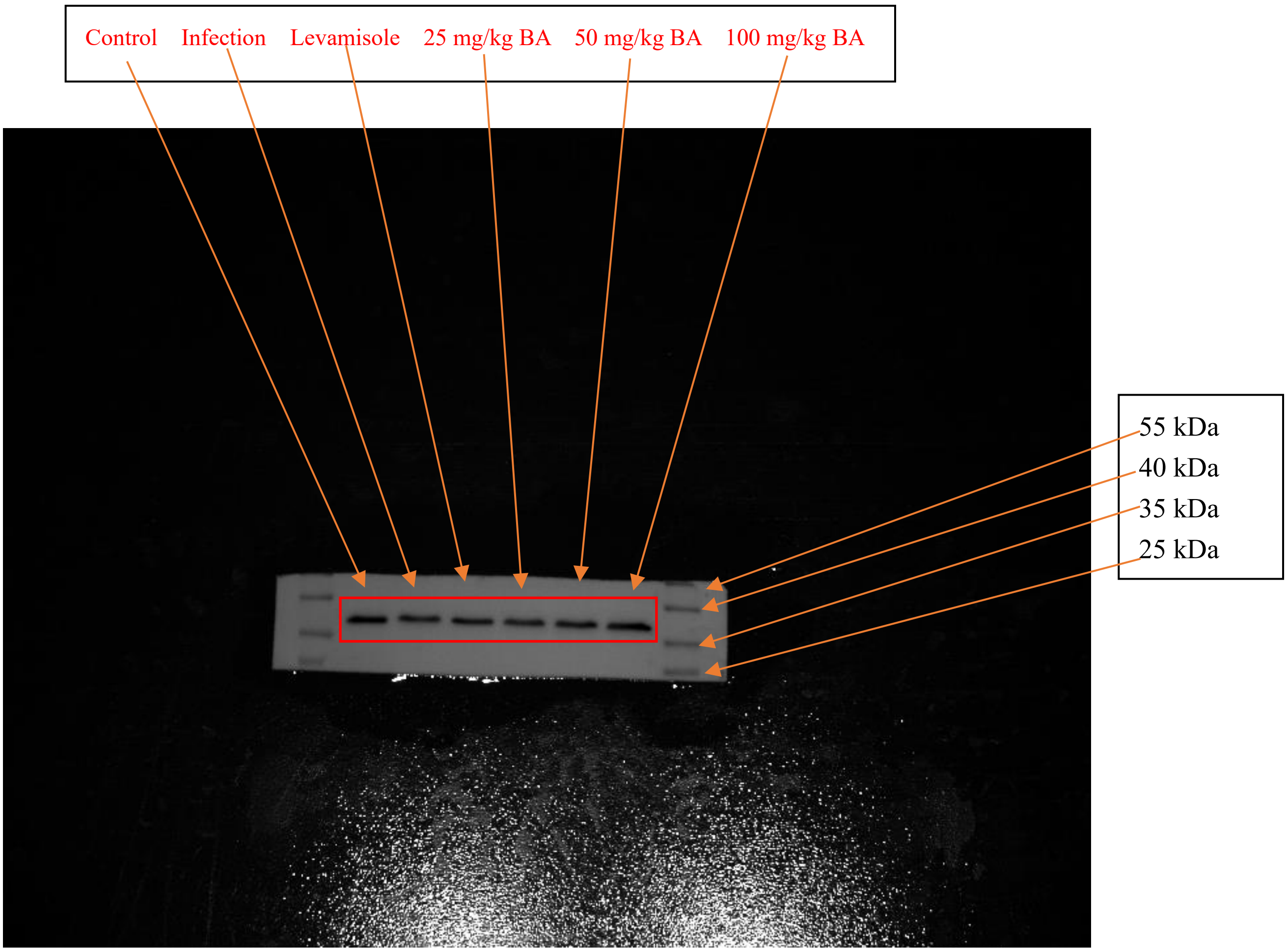

Fig 5

Fig 5. L:
GAPDH (repeat 2)

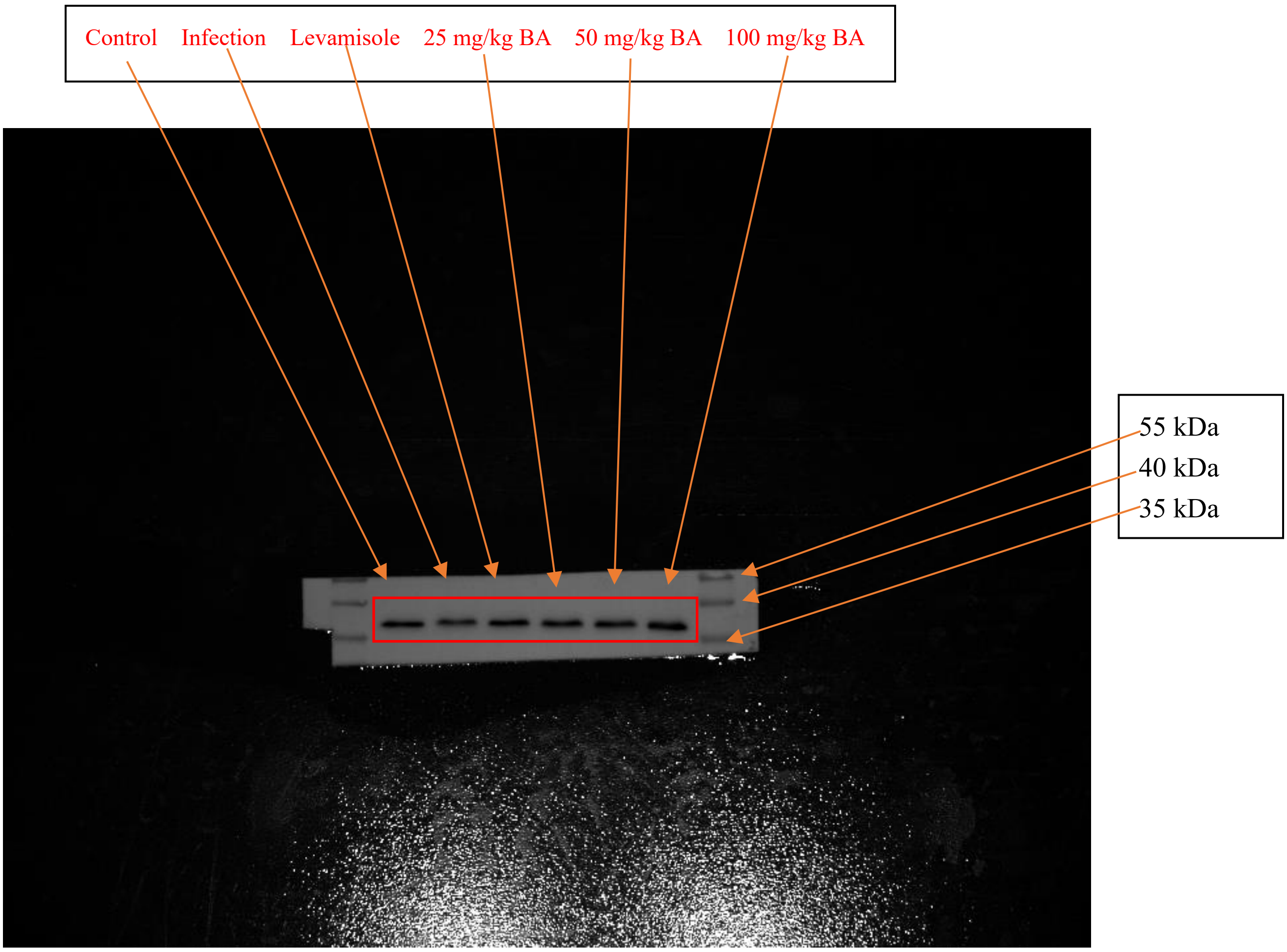

Fig 5

Fig 5. L:
GAPDH (repeat 3)

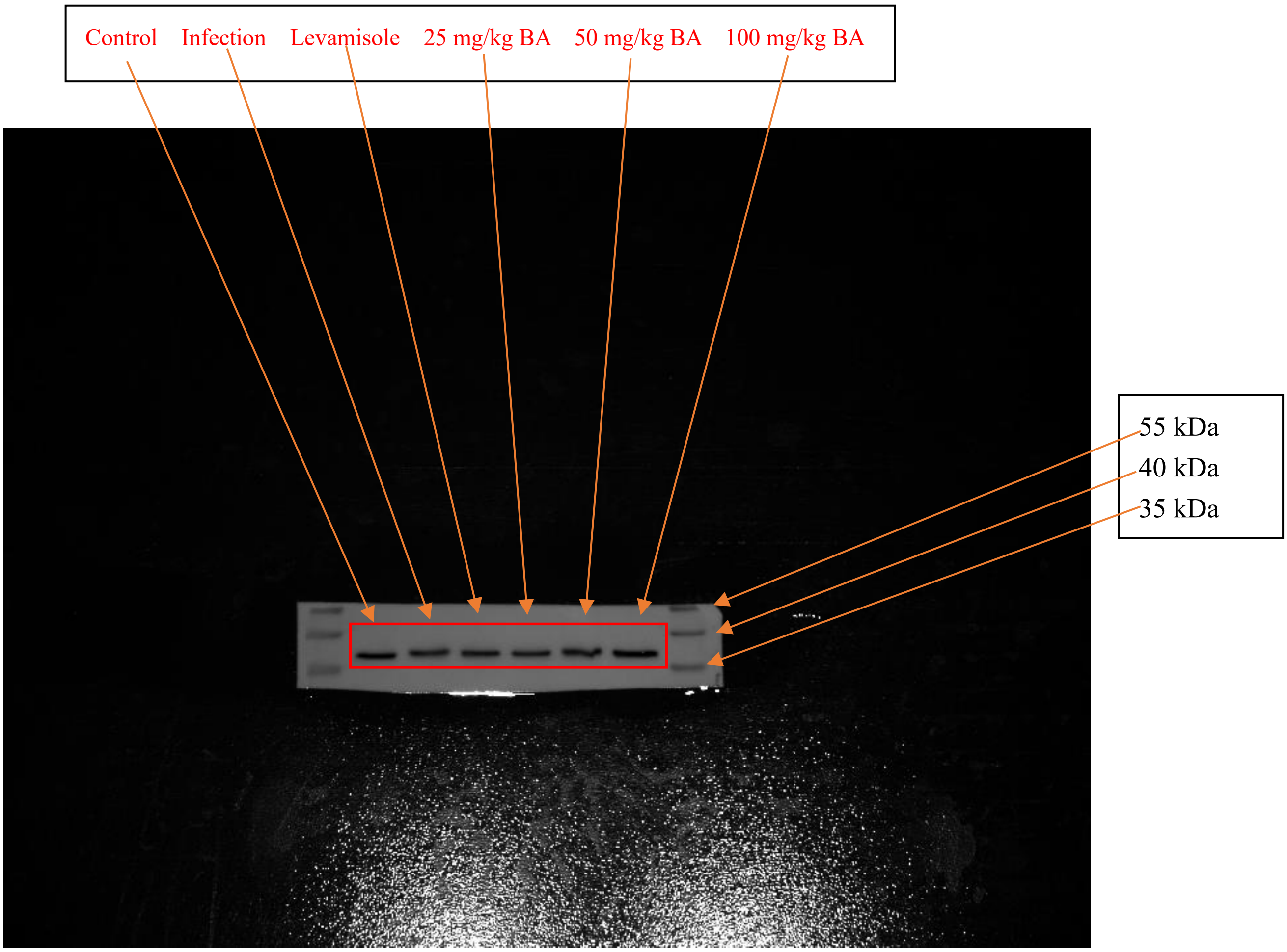

Fig 5

Fig 5. L:

β-catenin (repeat 1)

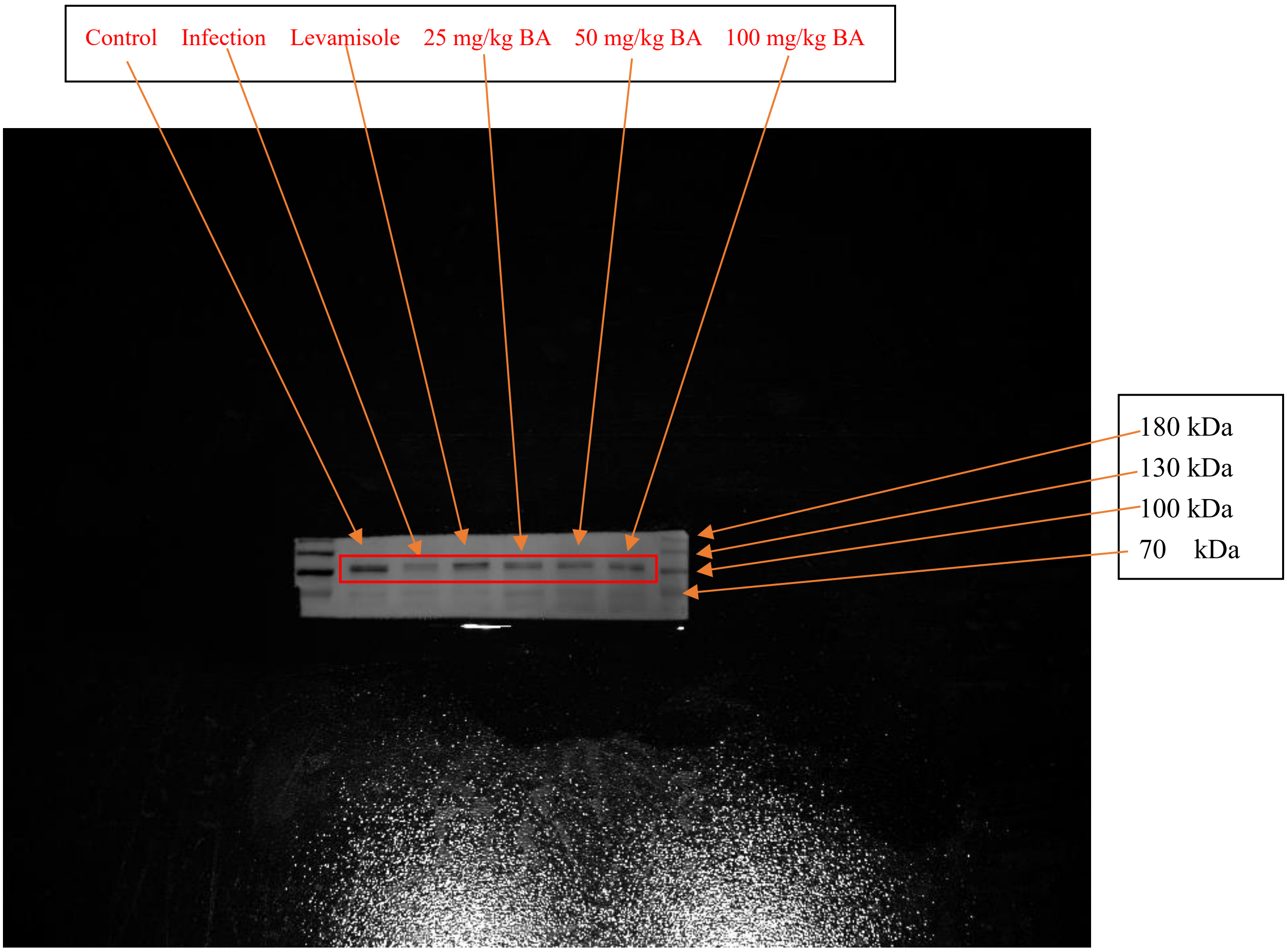

Fig 5

Fig 5. L:

β-catenin (repeat 2)

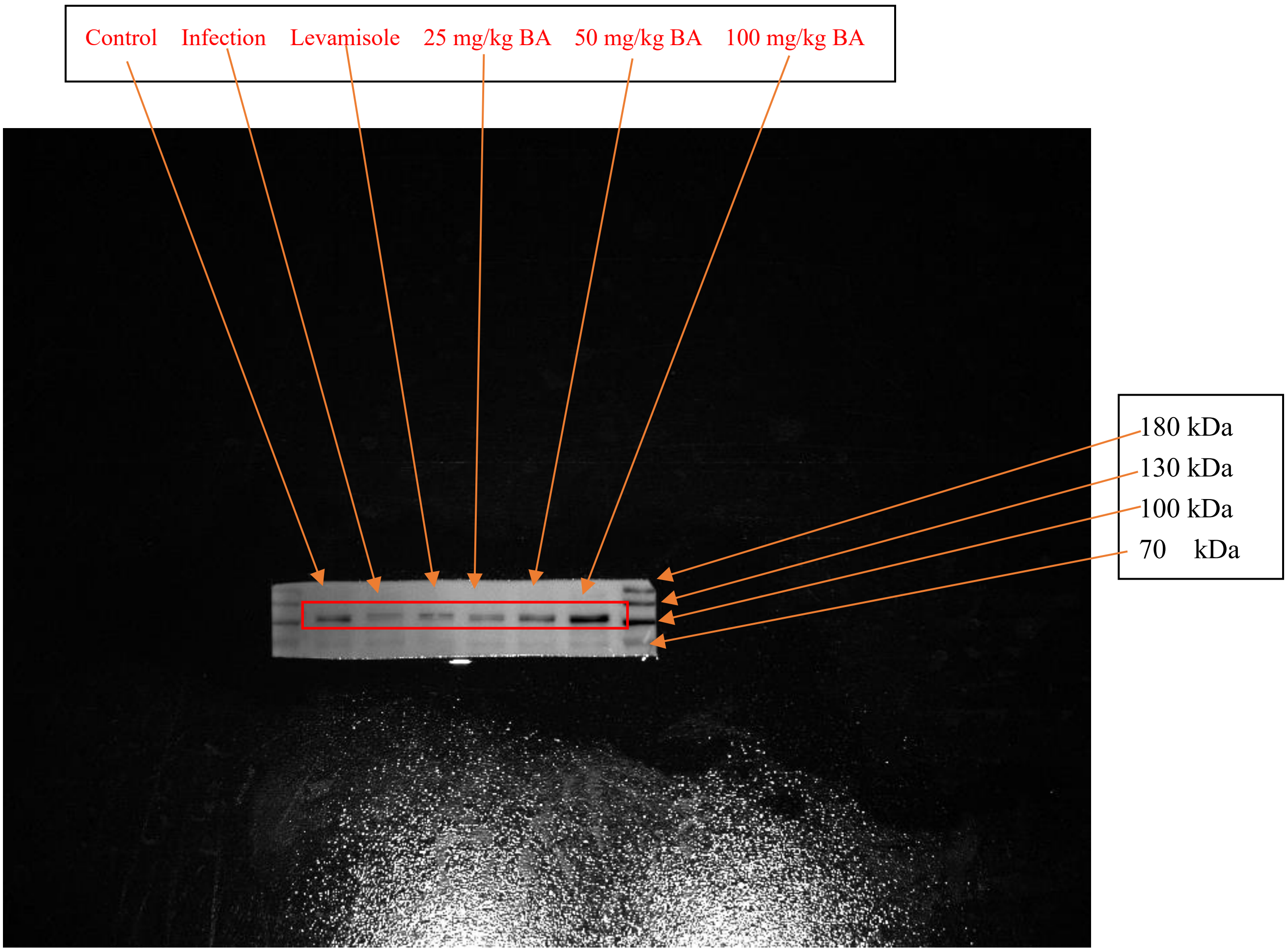

Fig 5

Fig 5. L:

β-catenin (repeat 3)

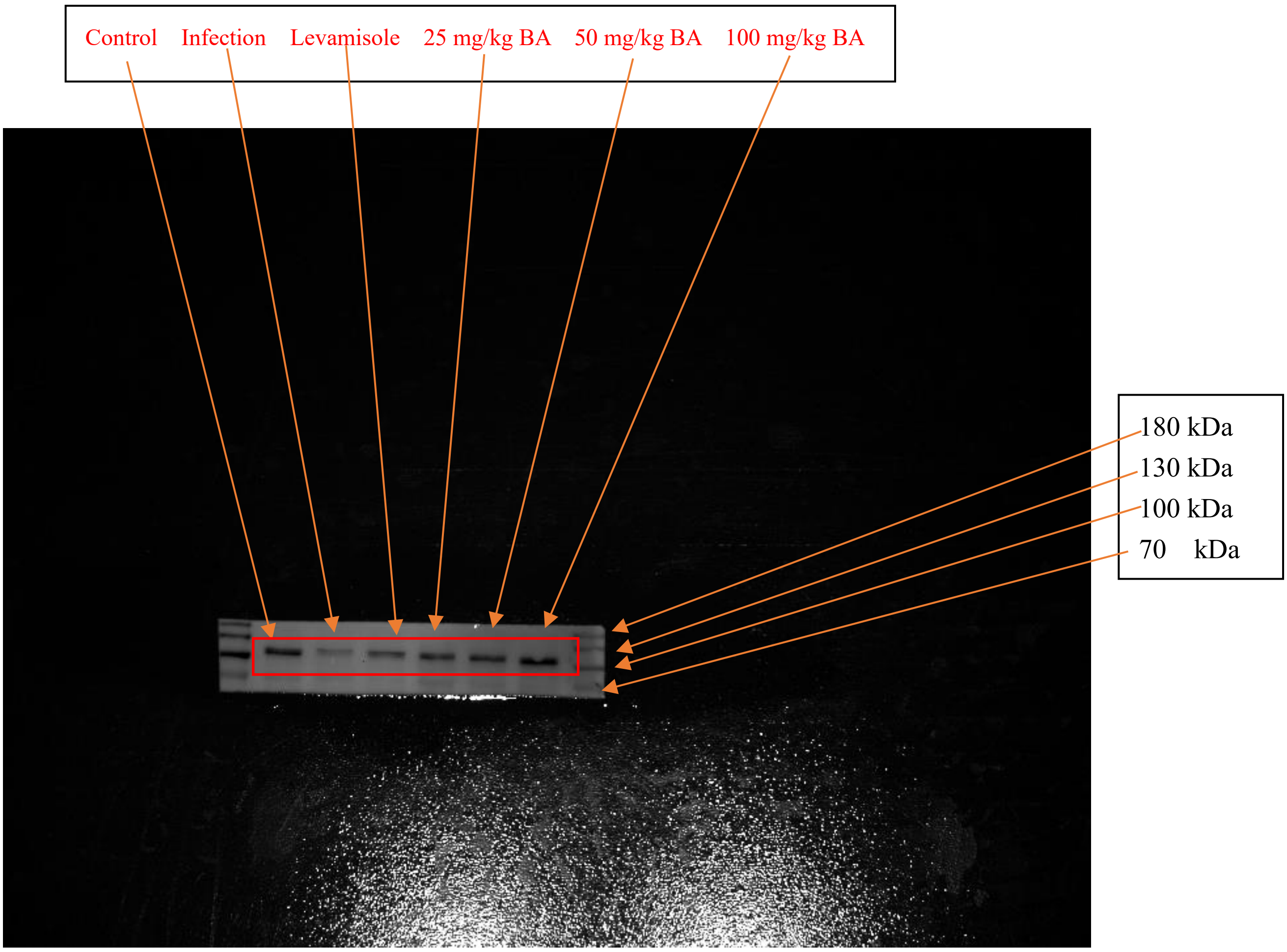

Fig 5

Fig 5. N:

GAPDH (repeat 1)

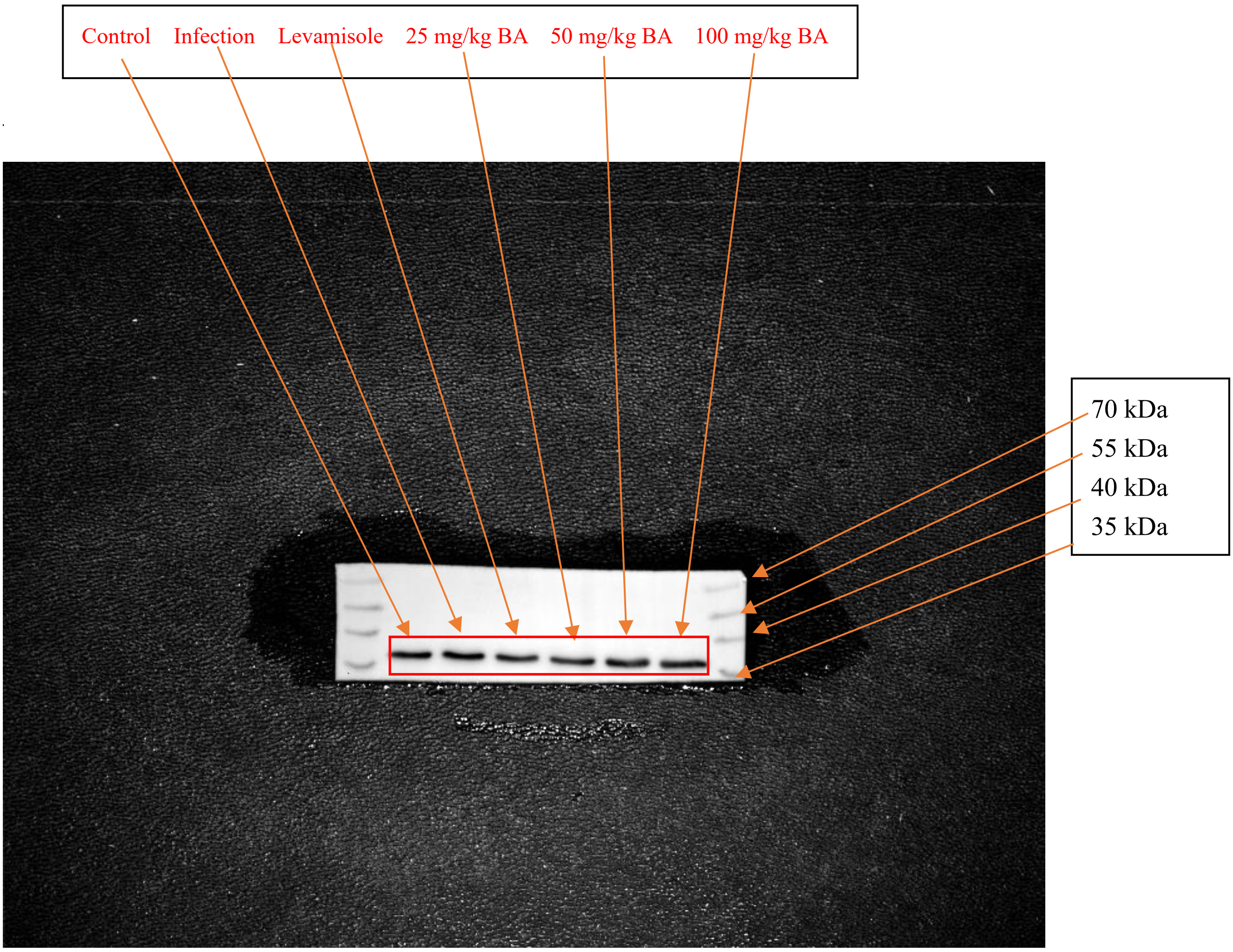

Fig 5

Fig 5. N:
GAPDH (repeat 2)

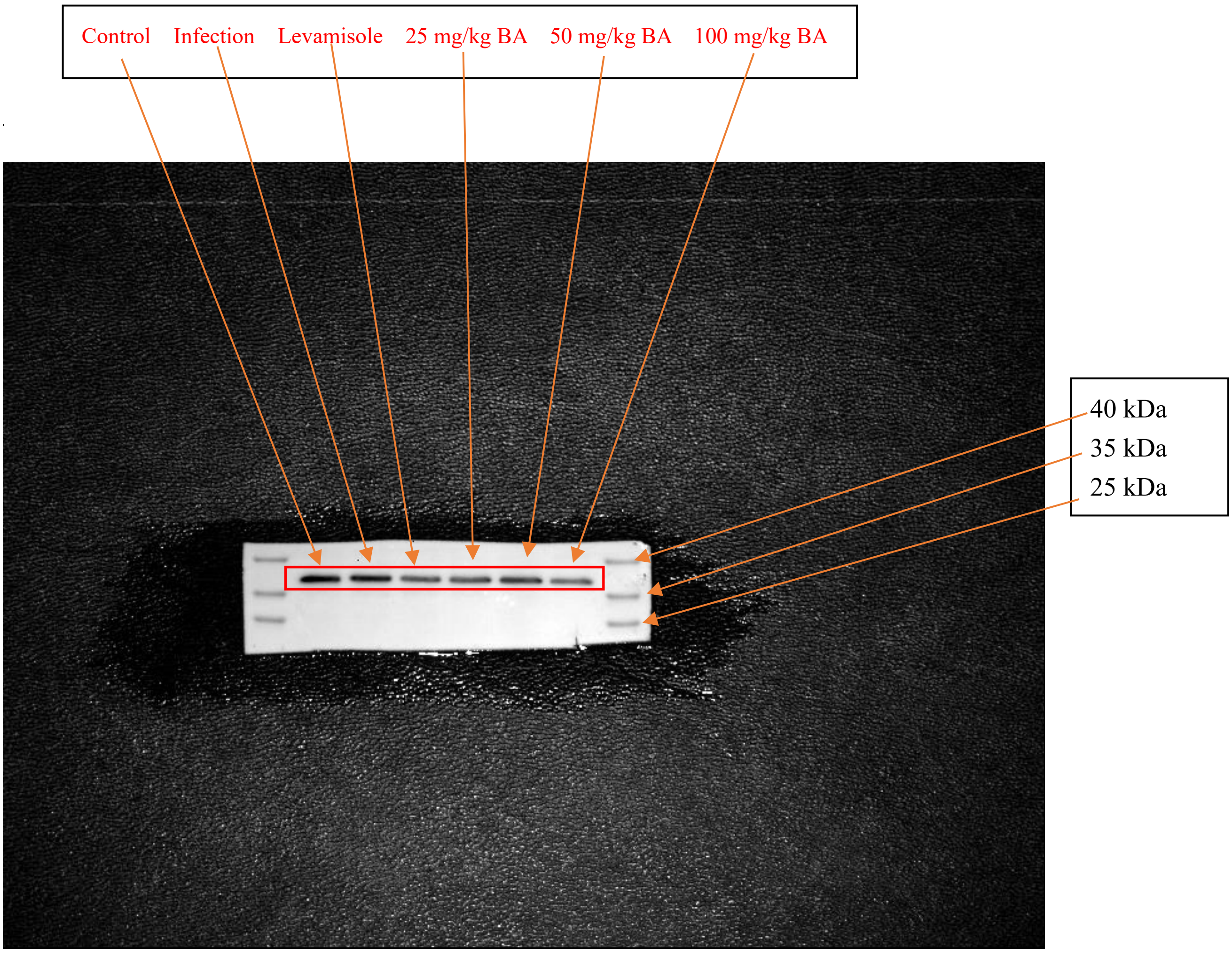

Fig 5

Fig 5. N:
GAPDH (repeat 3)

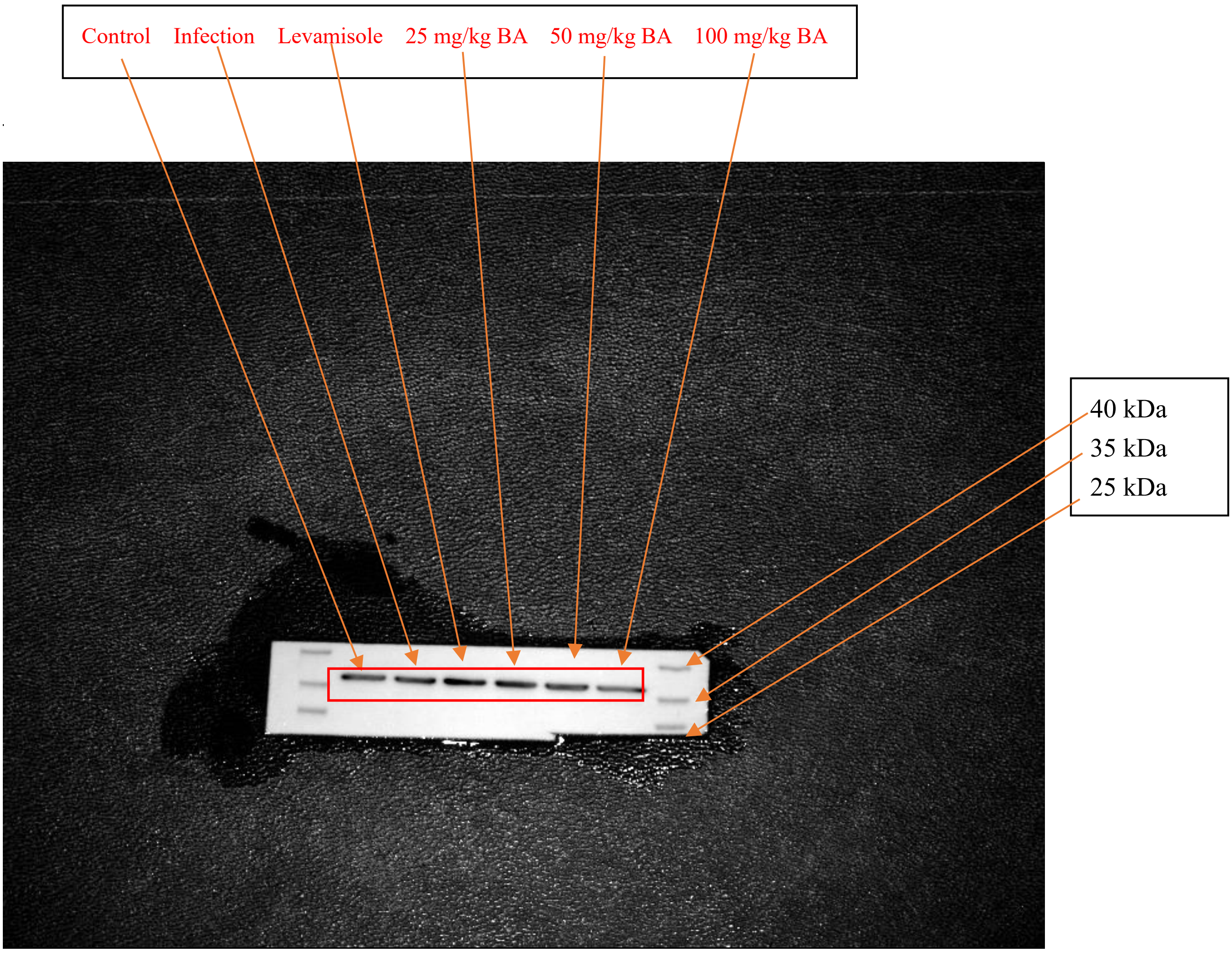

Fig 5

Fig 5. N:

HES1 (repeat 1)

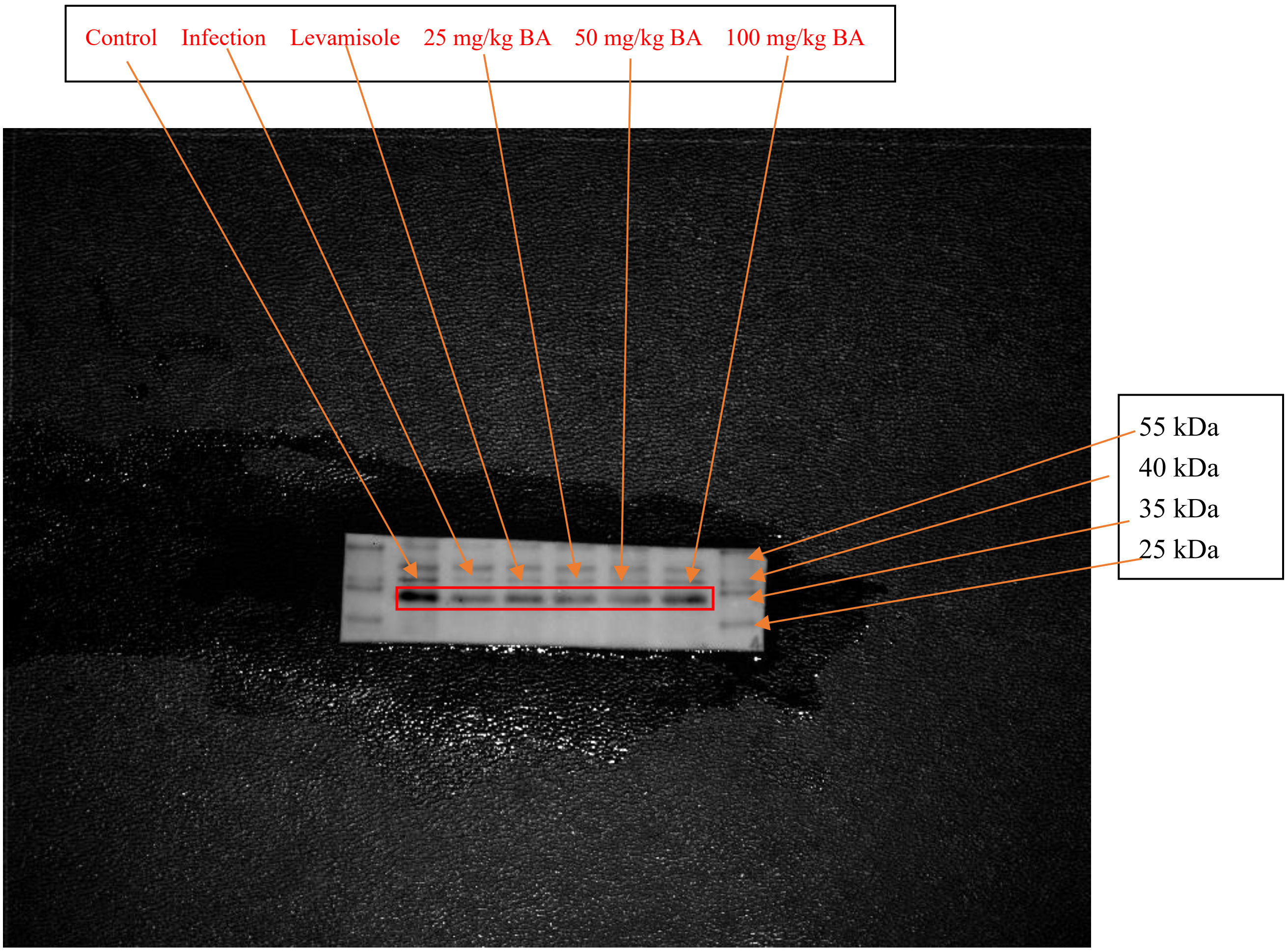

Fig 5

Fig 5. N:

HES1 (repeat 2)

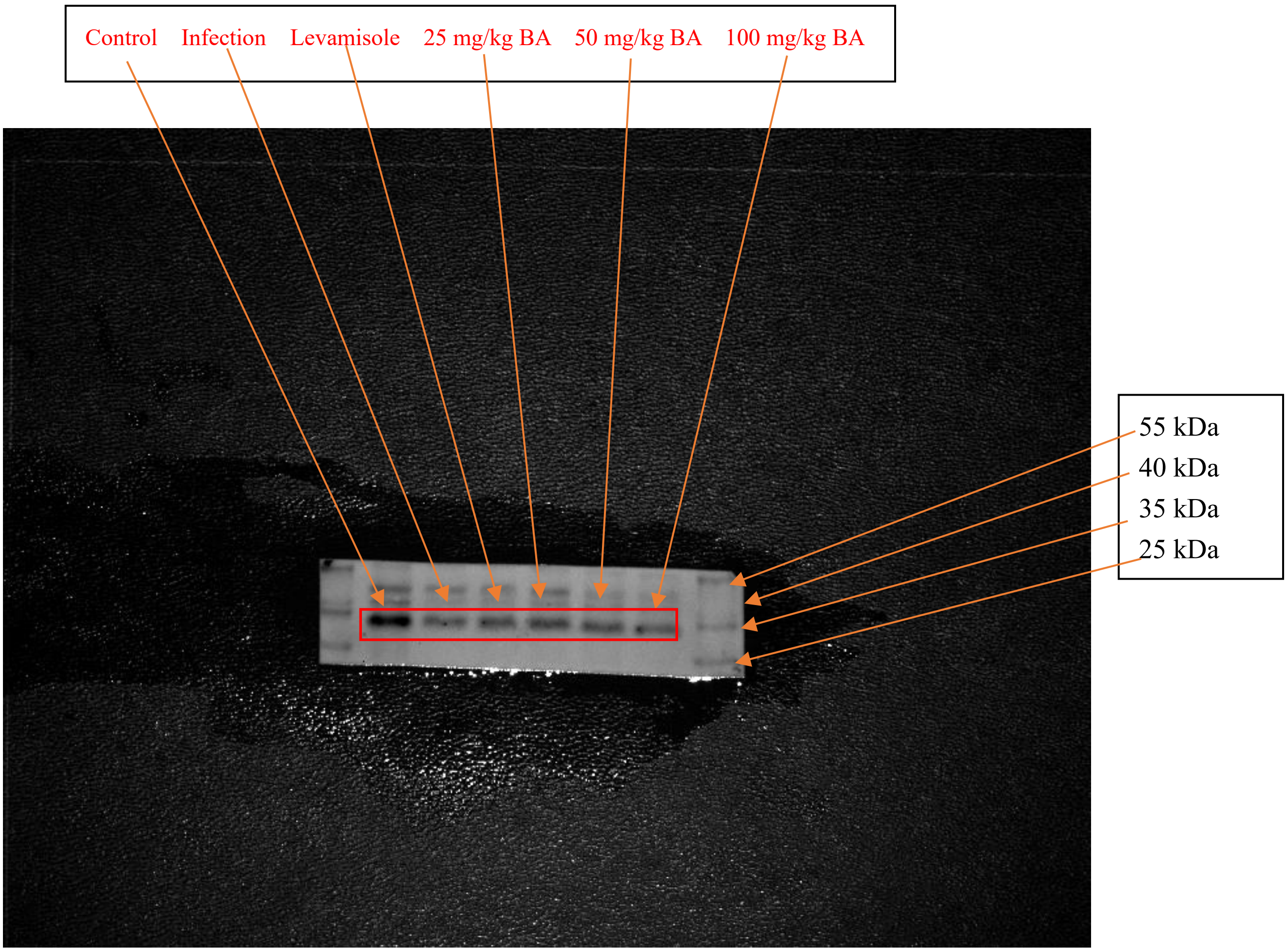

Fig 5

Fig 5. N:

HES1 (repeat 3)

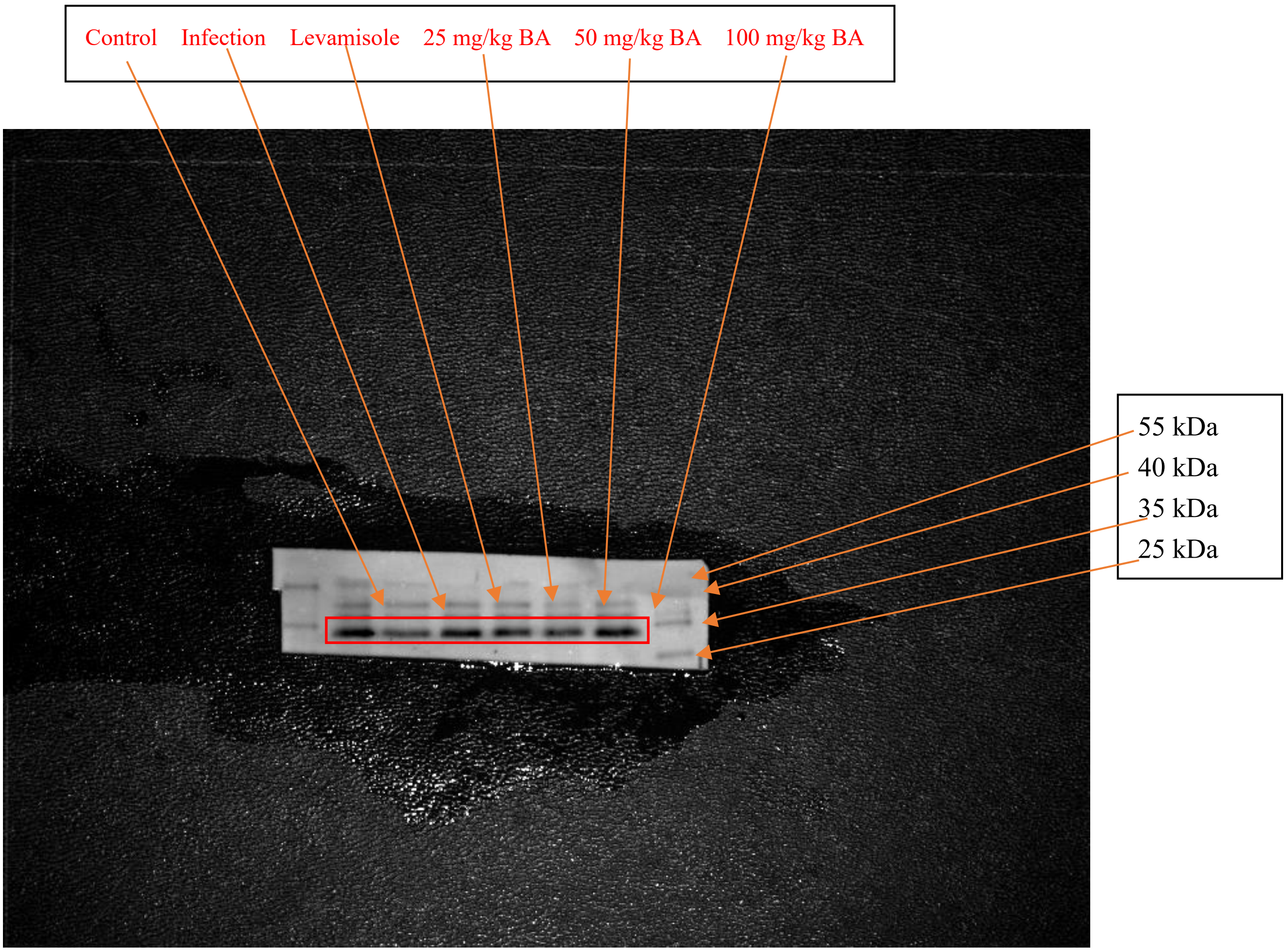

Fig 5

Fig 5. P:

GAPDH (repeat 1)

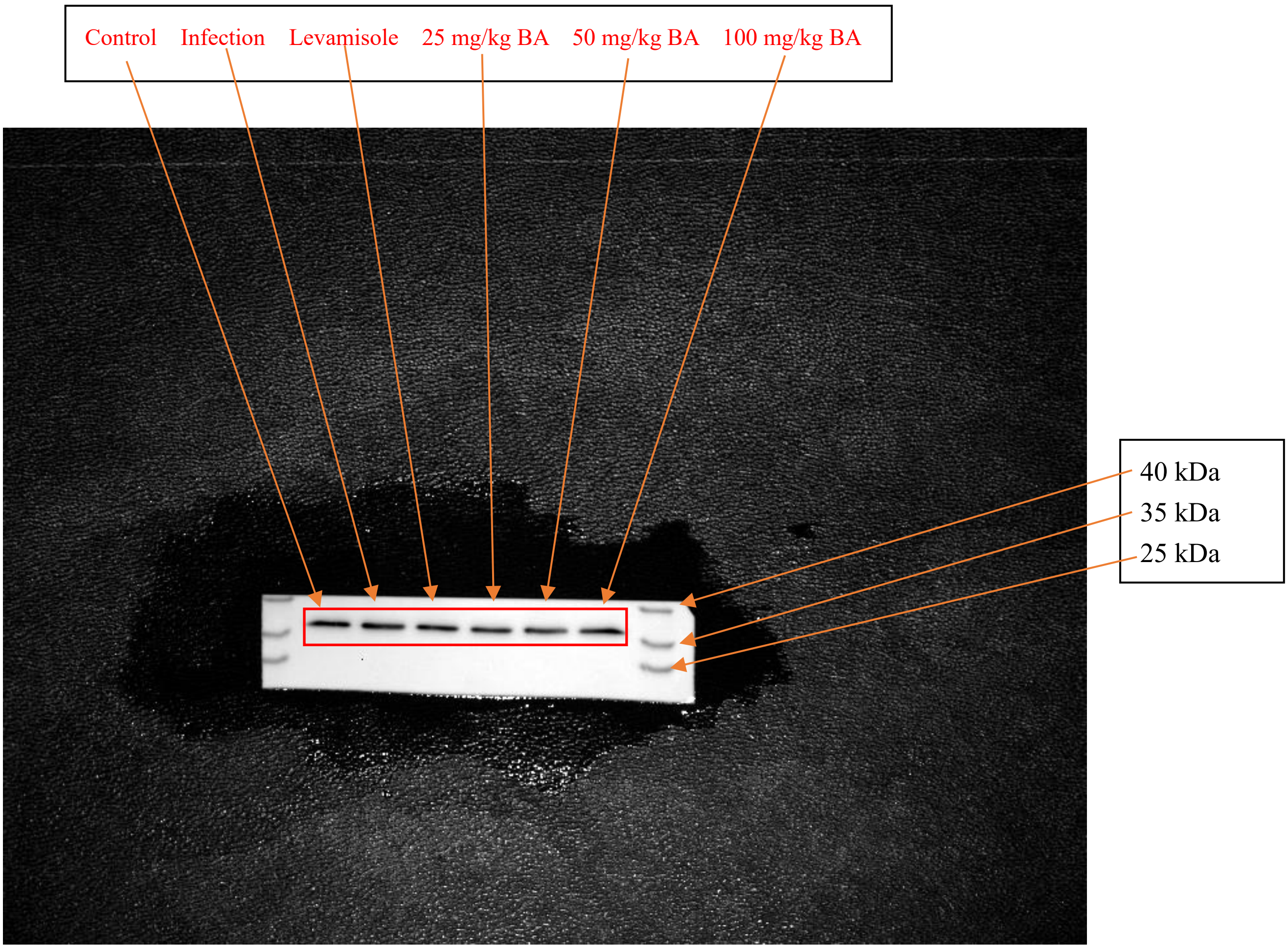

Fig 5

Fig 5. P:

GAPDH (repeat 2)

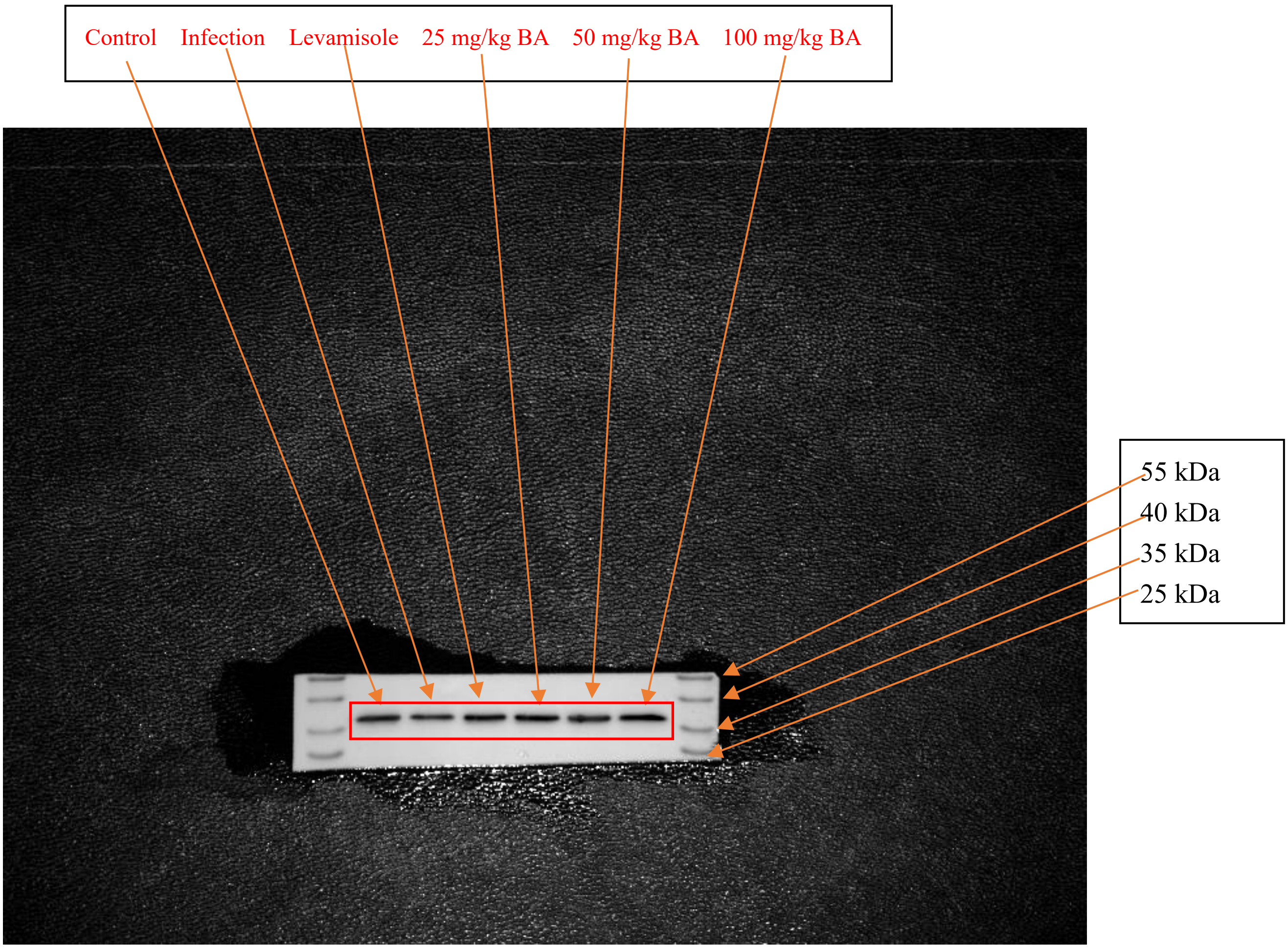

Fig 5

Fig 5. P:

GAPDH (repeat 3)

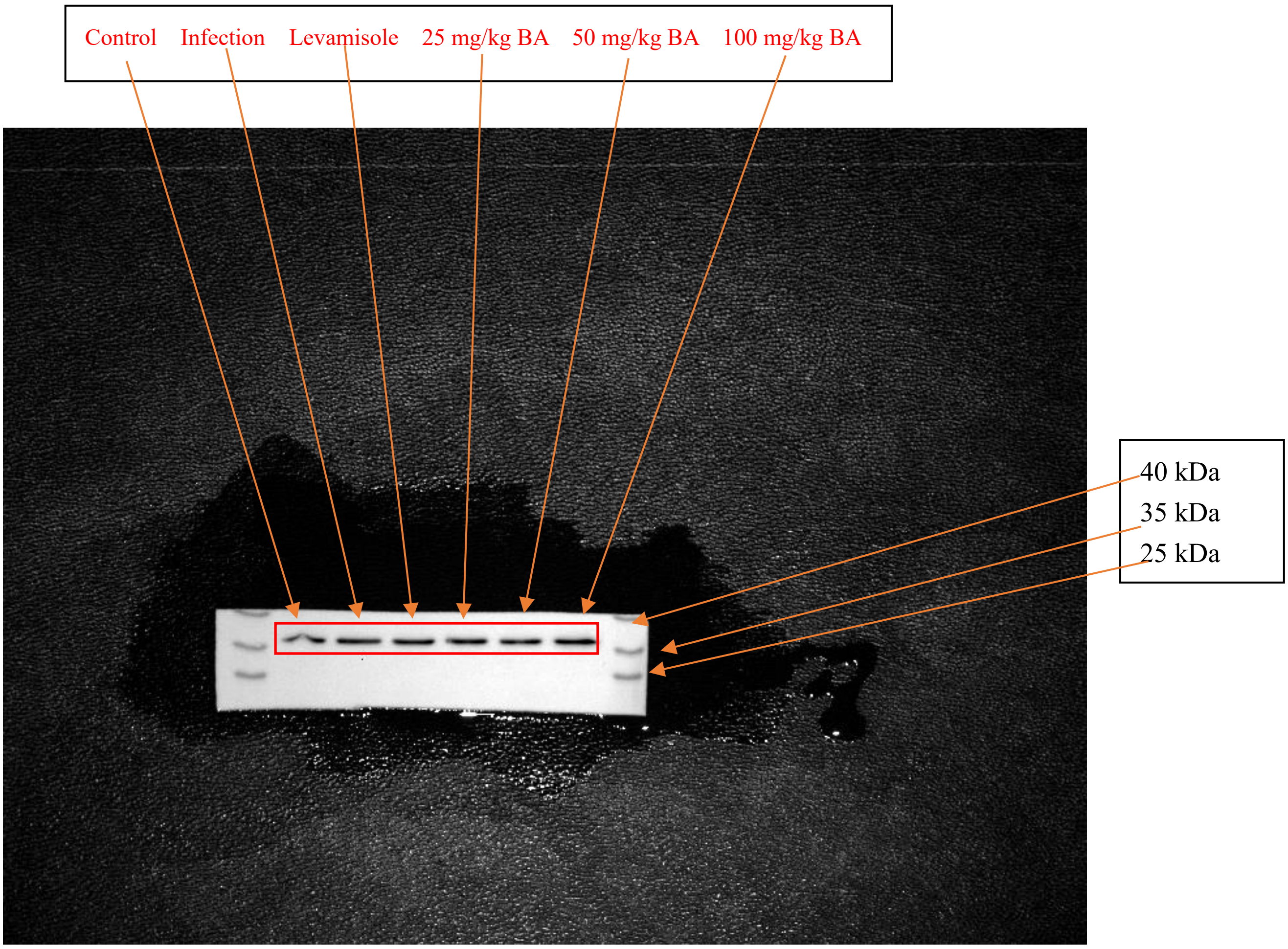

Fig 5

Fig 5. P:

c-Myc (repeat 1)

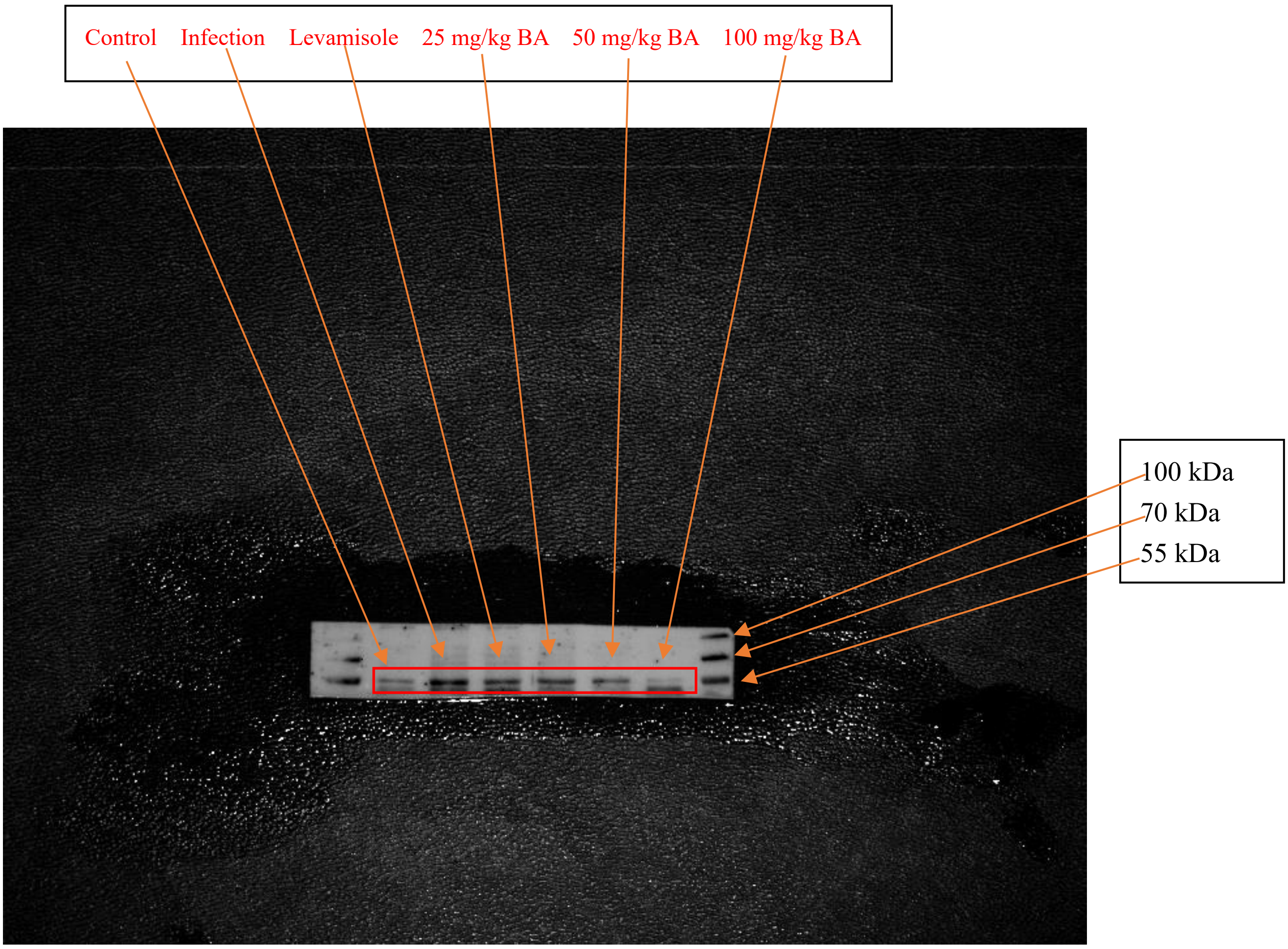

Fig 5

Fig 5. P:

c-Myc (repeat 2)

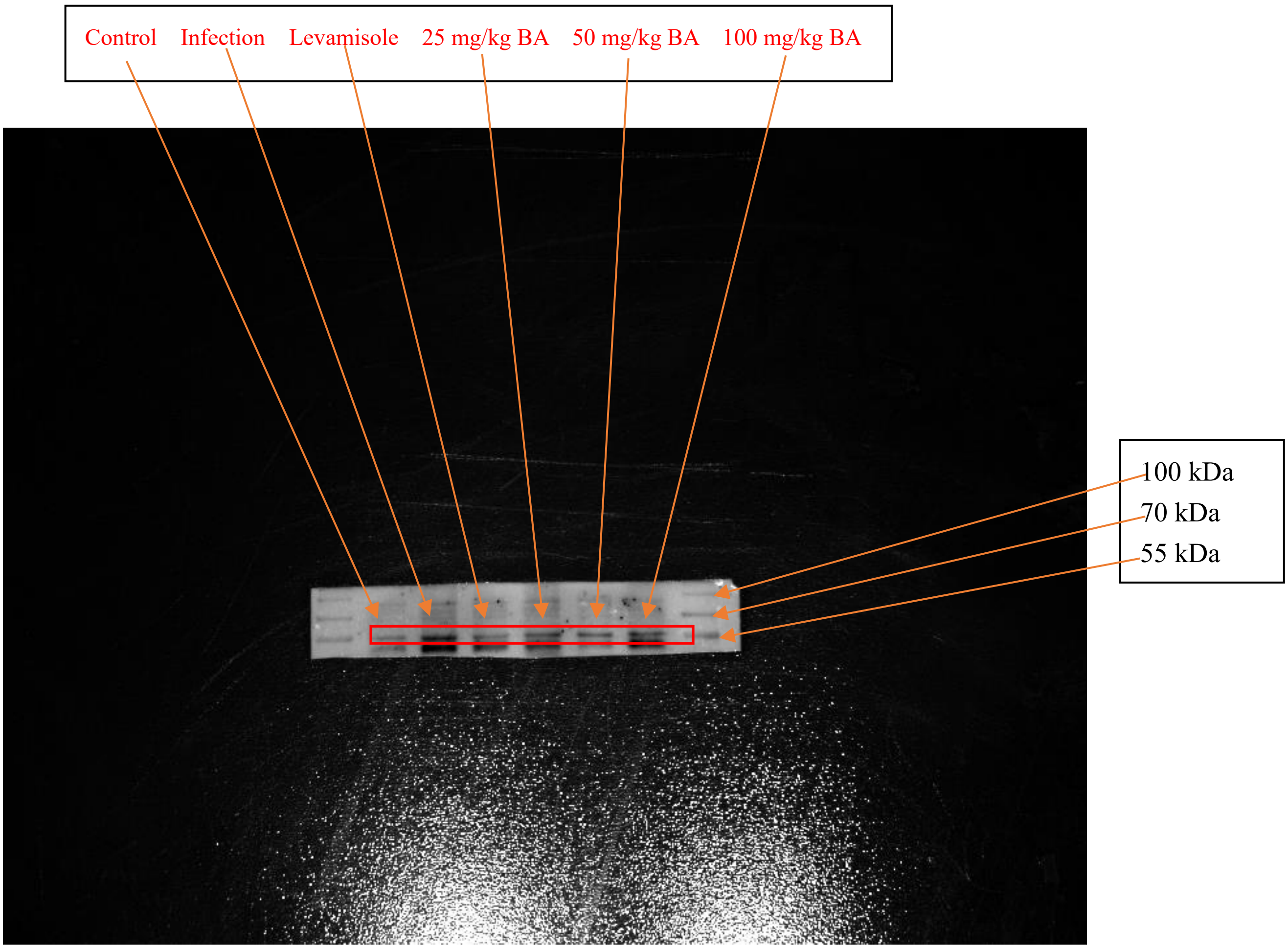

Fig 5

Fig 5. P:

c-Myc (repeat 3)

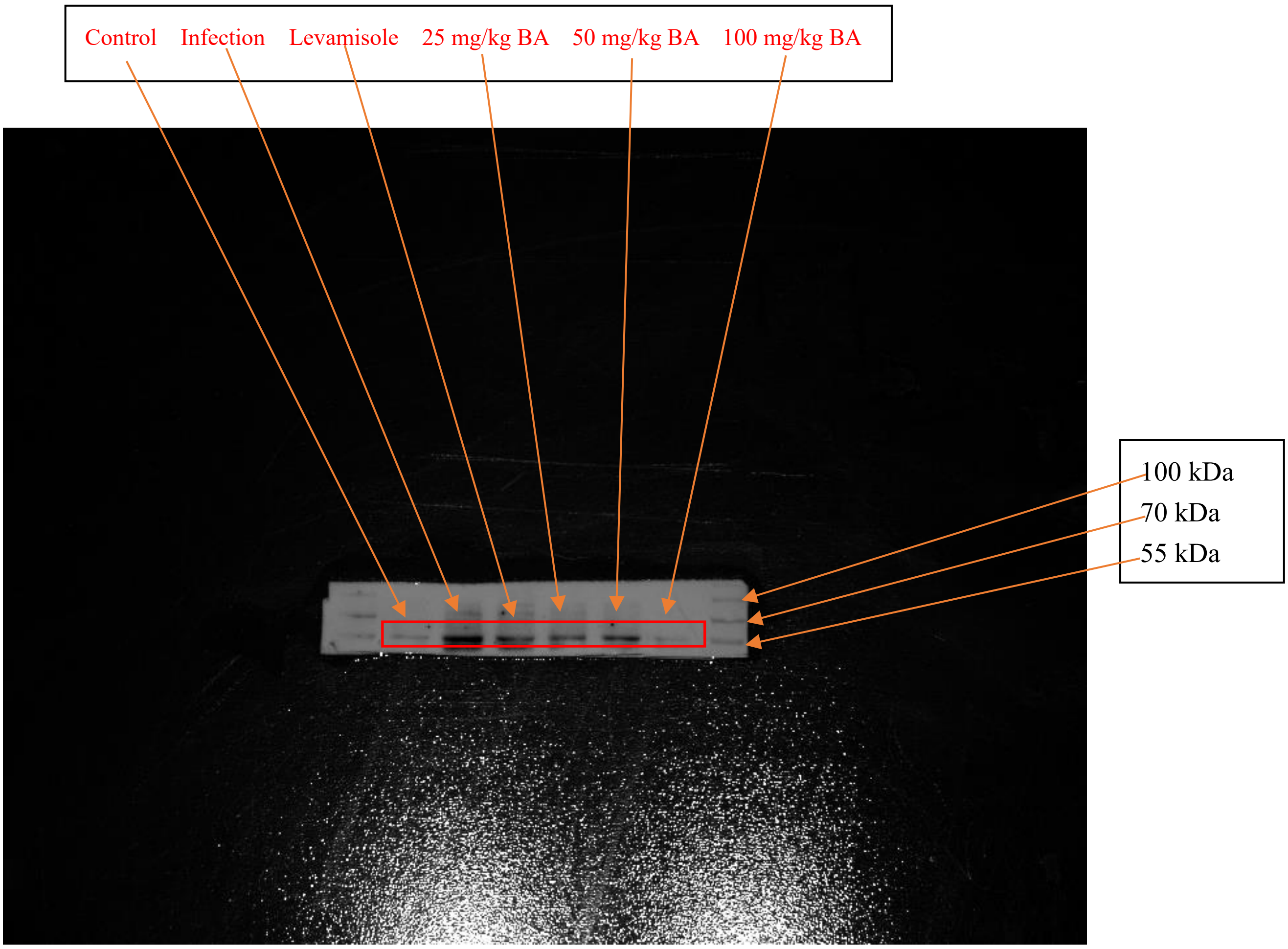

Fig 6

Fig 6. A:

GAPDH (repeat 1)

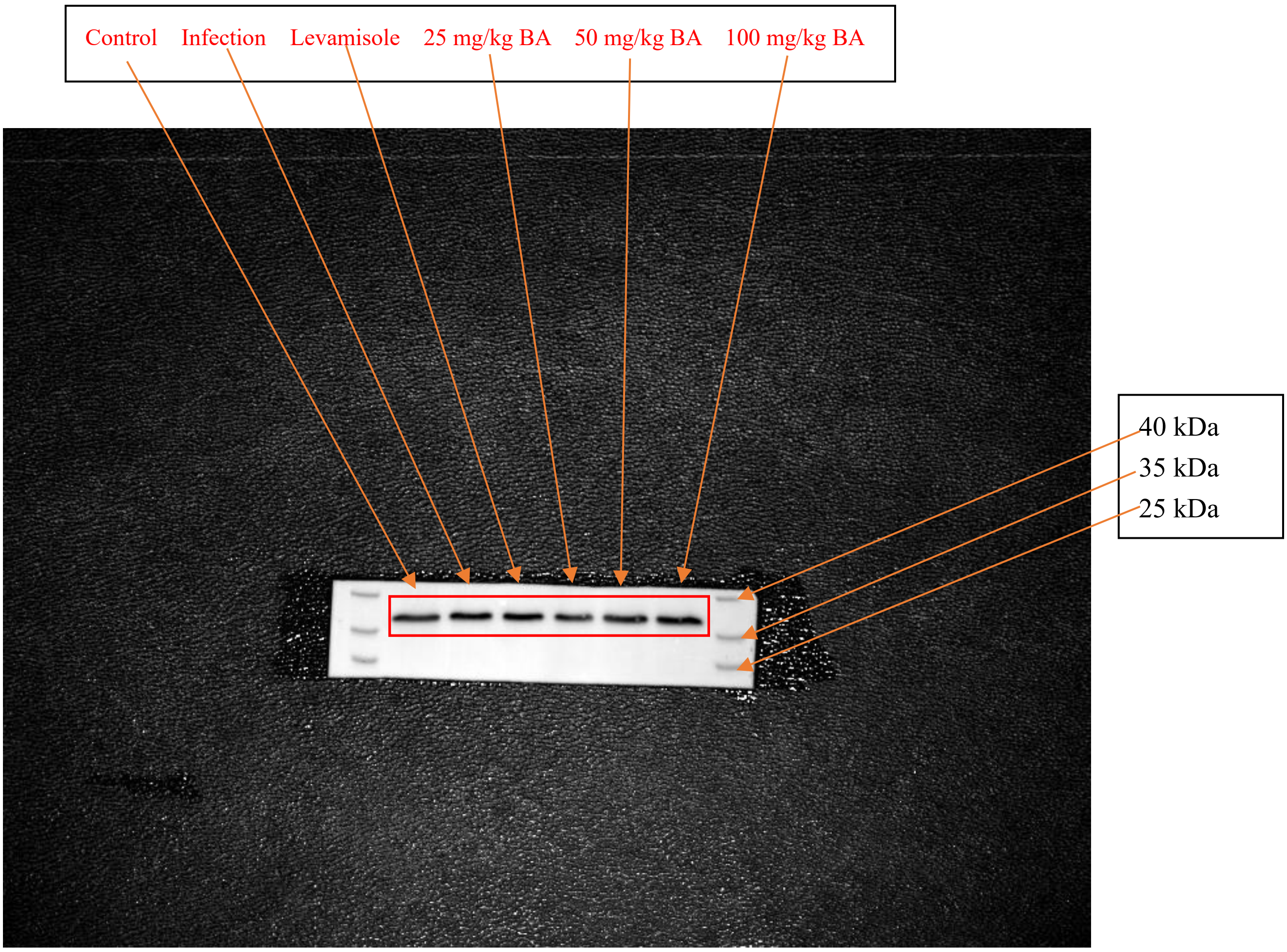

Fig 6

Fig 6. A:

GAPDH (repeat 2)

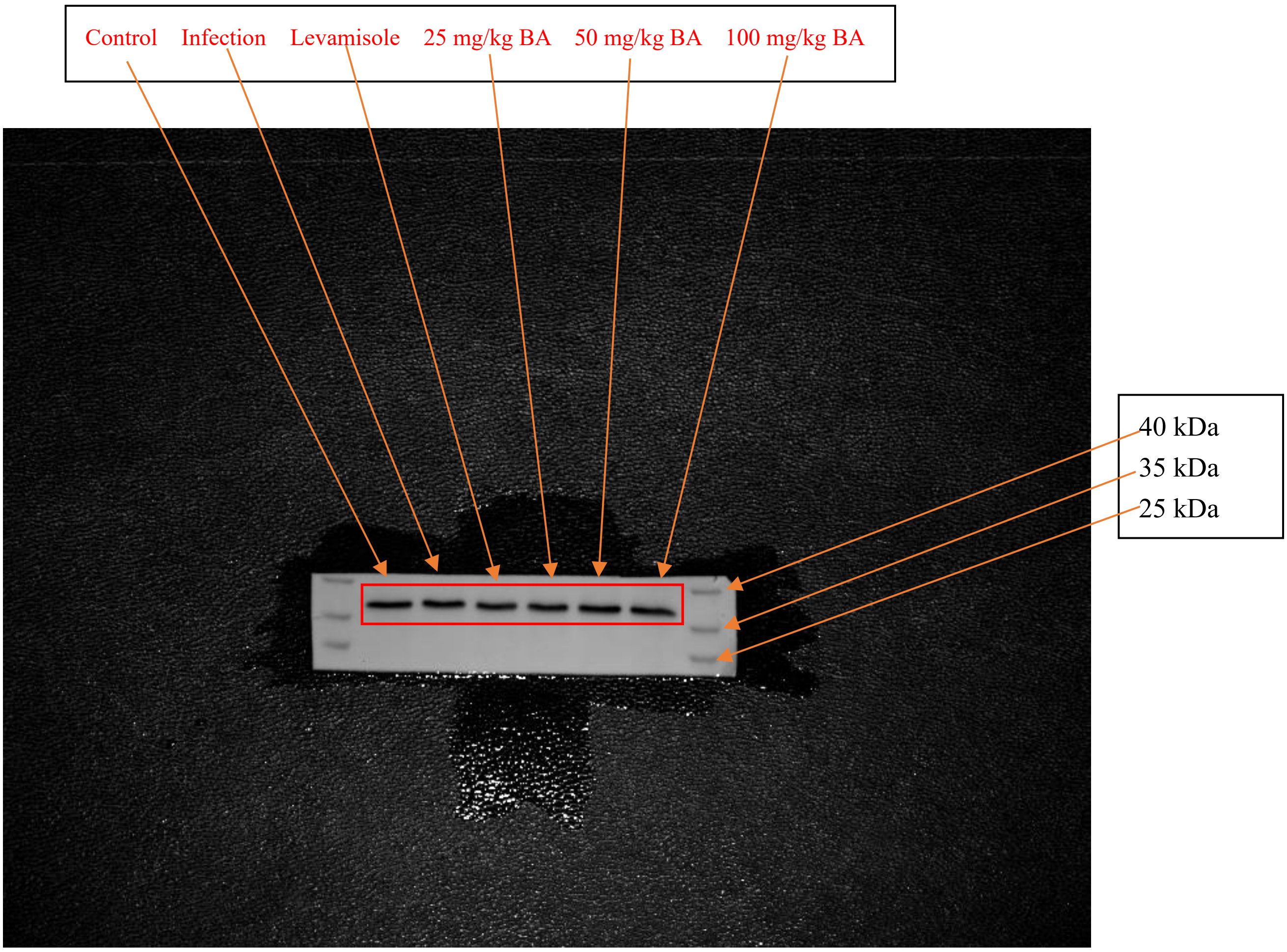

Fig 6

Fig 6. A:

GAPDH (repeat 3)

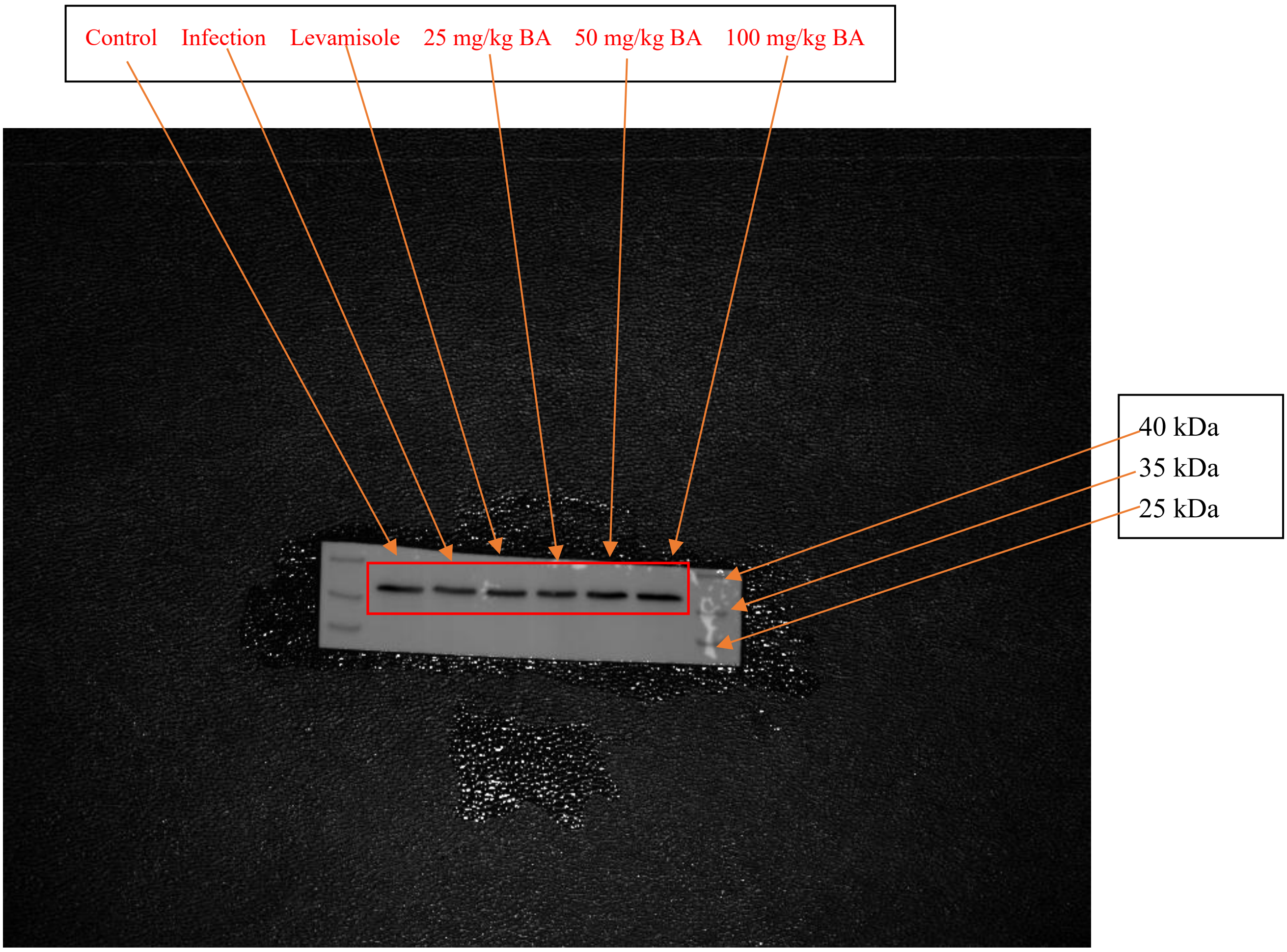

Fig 6

Fig 6. A:

Beclin1 (repeat 1)

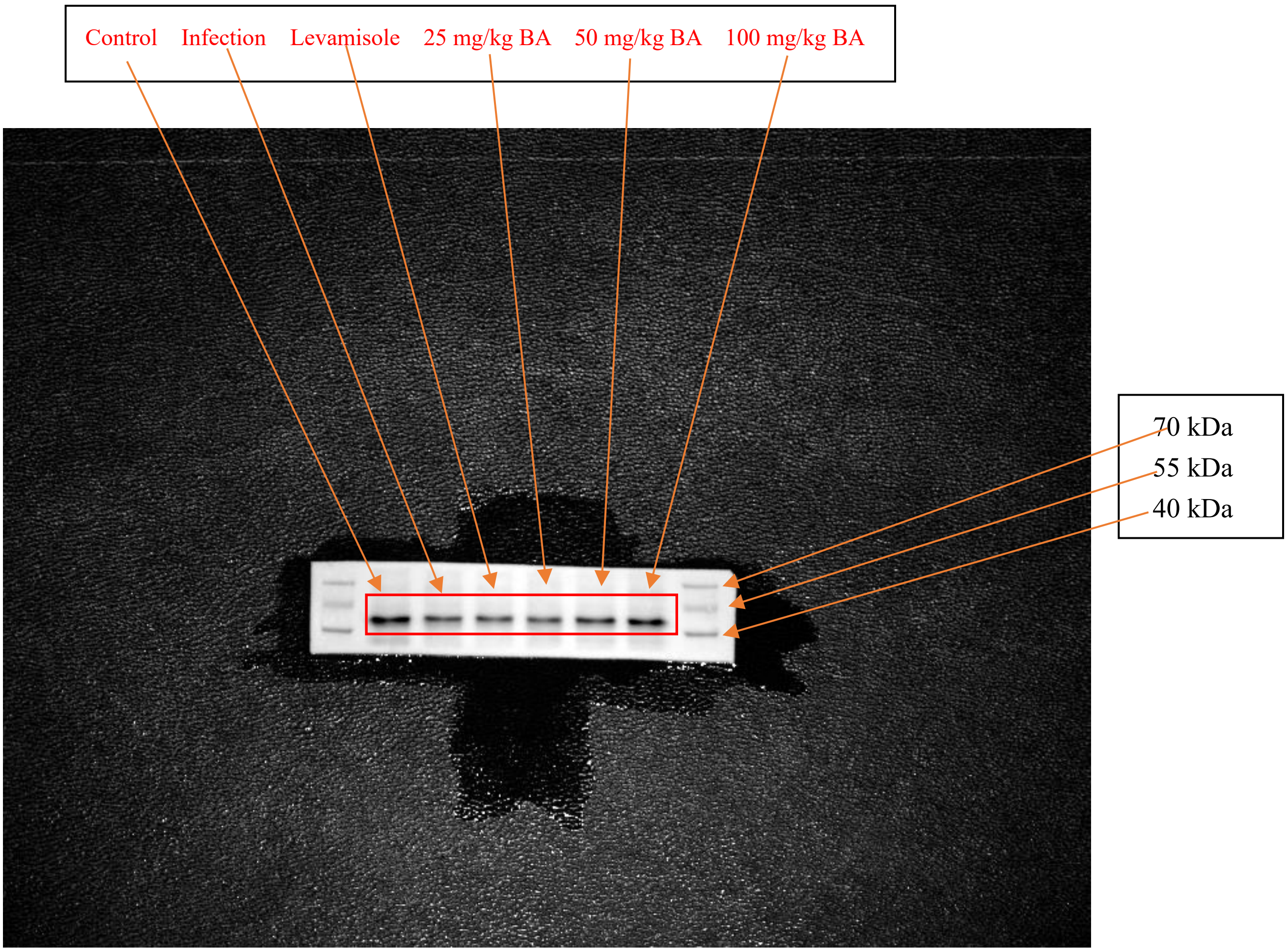

Fig 6

Fig 6. A:

Beclin1 (repeat 2)

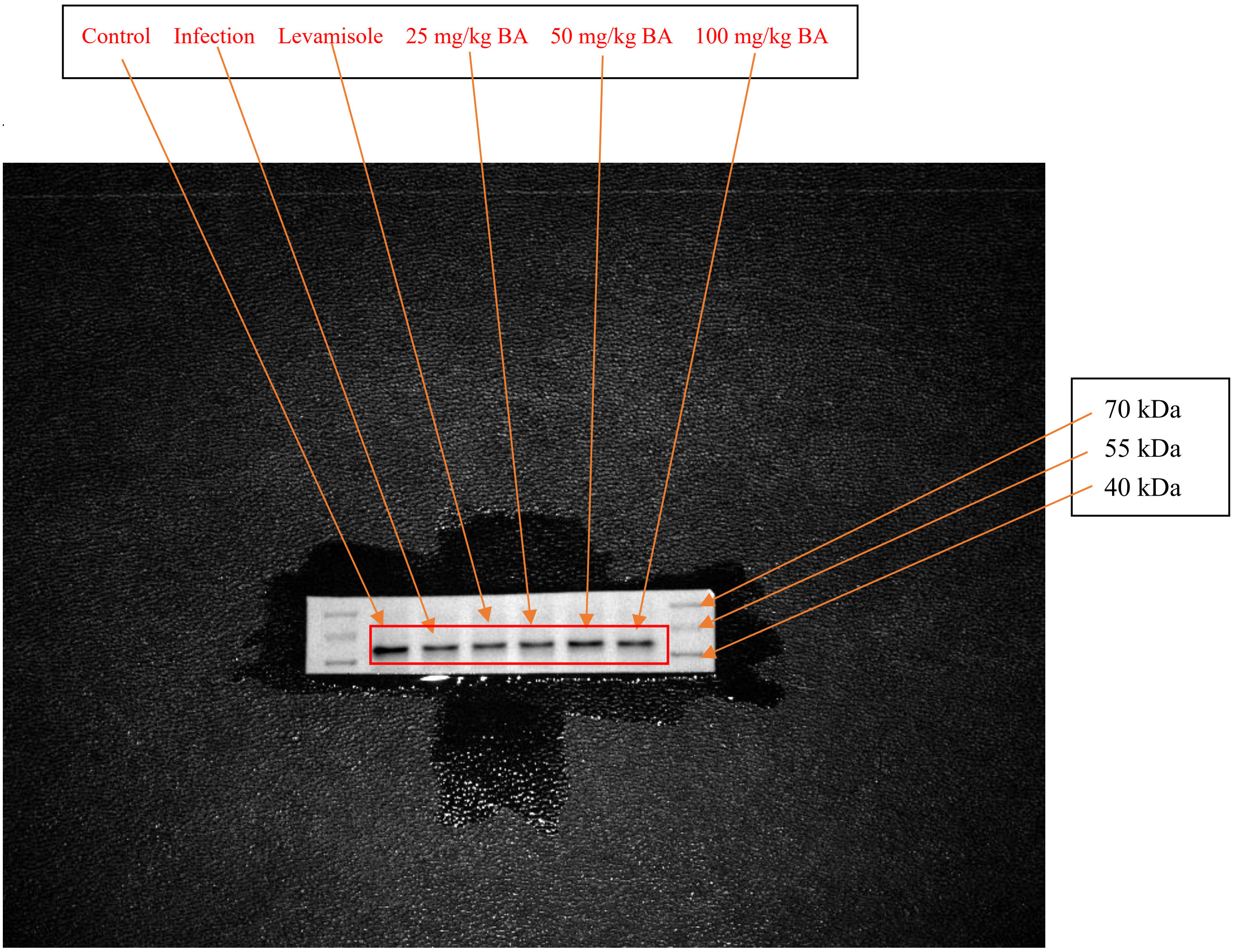

Fig 6

Fig 6. A:

Beclin1 (repeat 3)

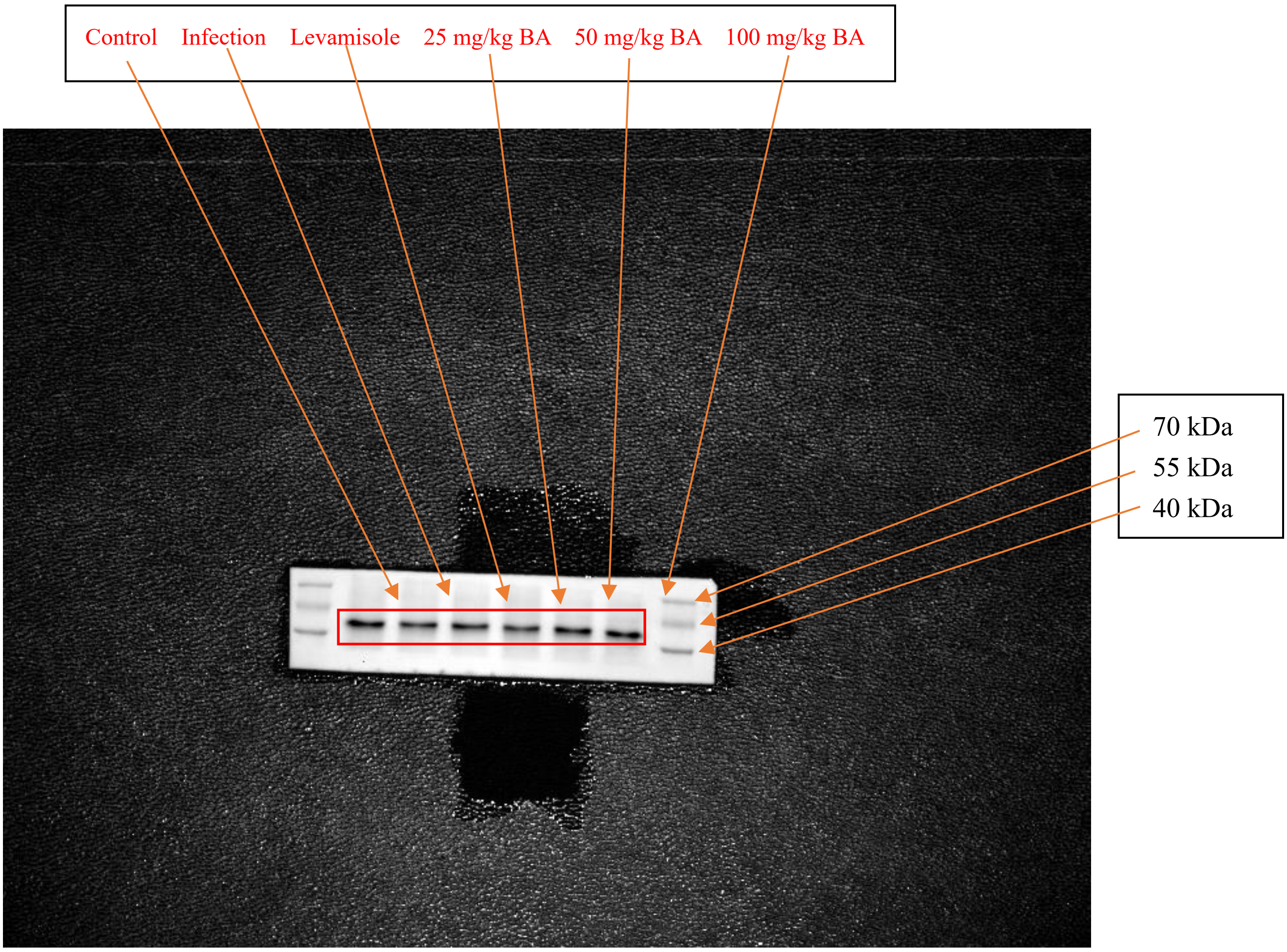

Fig 6

Fig 6. C:

GAPDH (repeat 1)

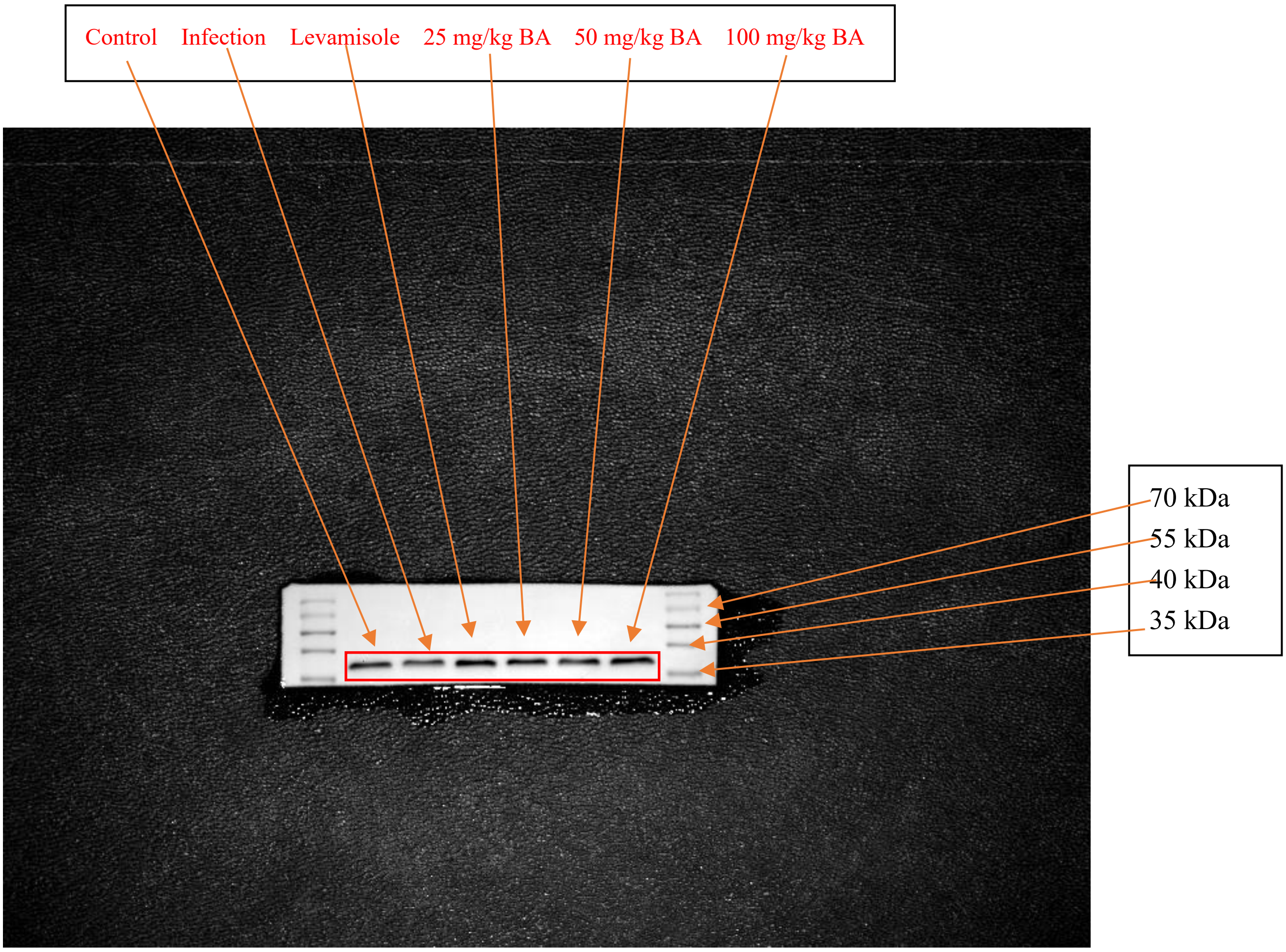

Fig 6

Fig 6. C:
GAPDH (repeat 2)

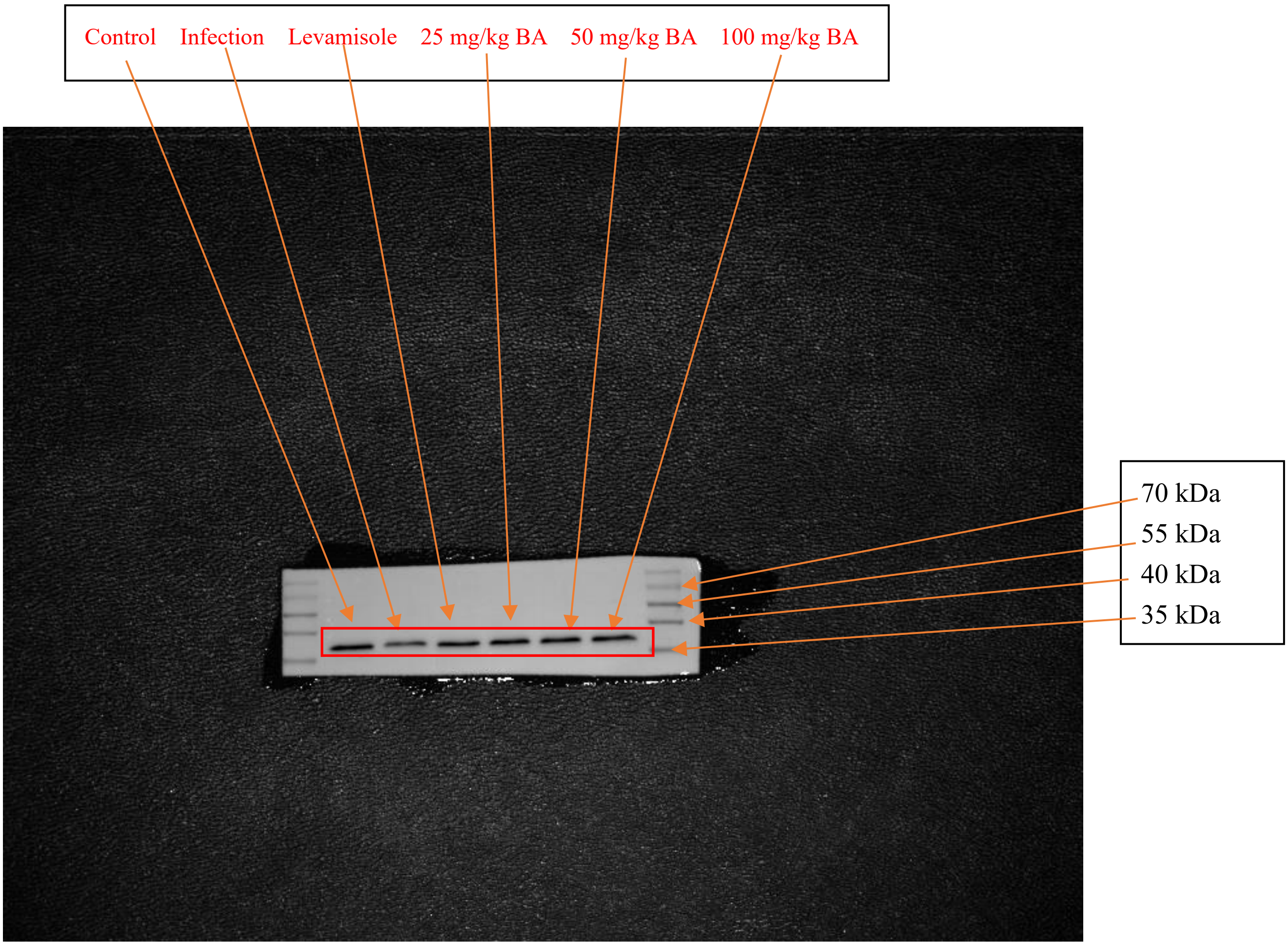

Fig 6

Fig 6. C:
GAPDH (repeat 3)

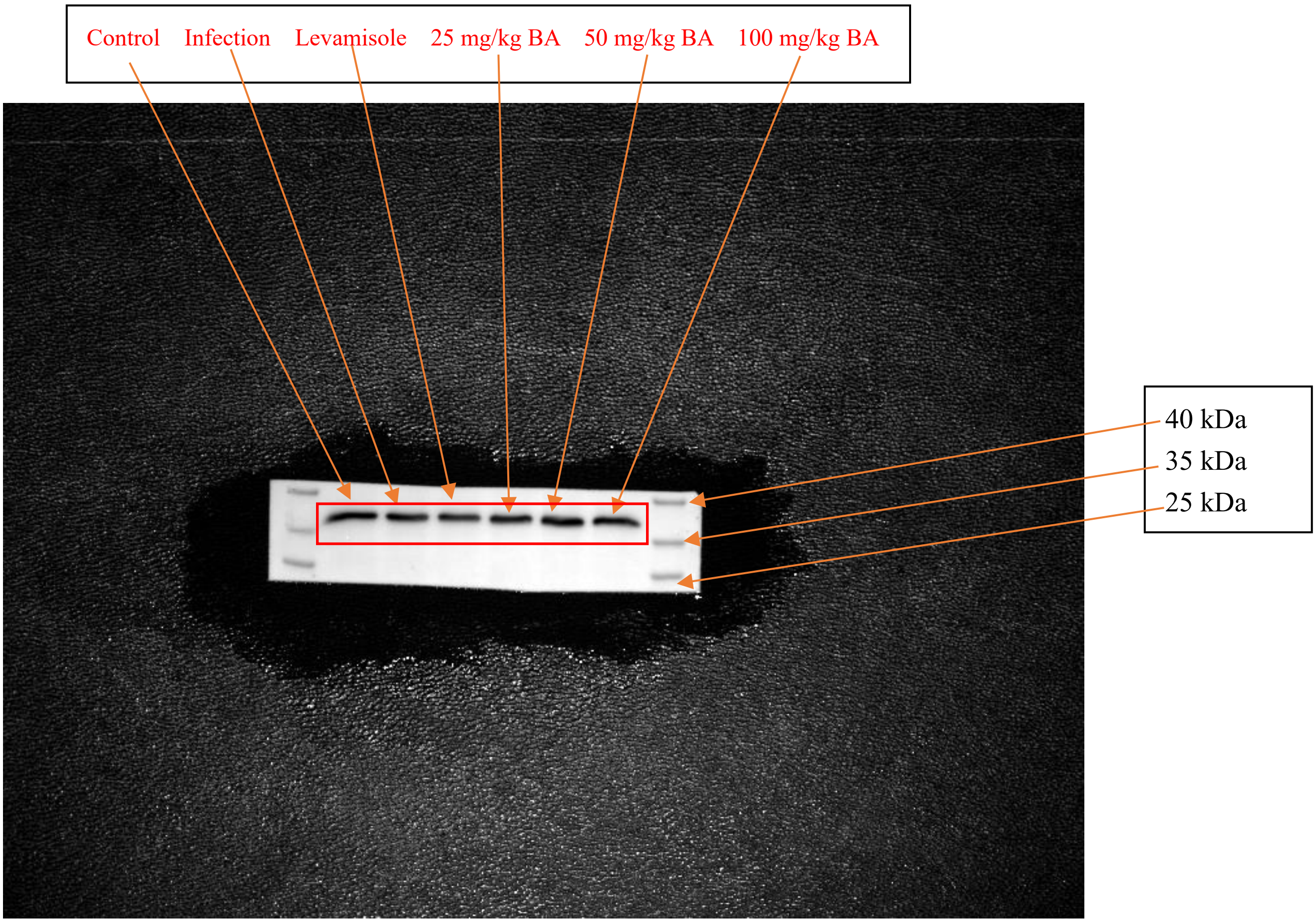

Control
Infection
Levamisole
25 mg/kg BA
50 mg/kg BA
100 mg/kg BA
40 kDa
35 kDa
25 kDa

Fig 6

Fig 6. B:

LC3B (repeat 1)

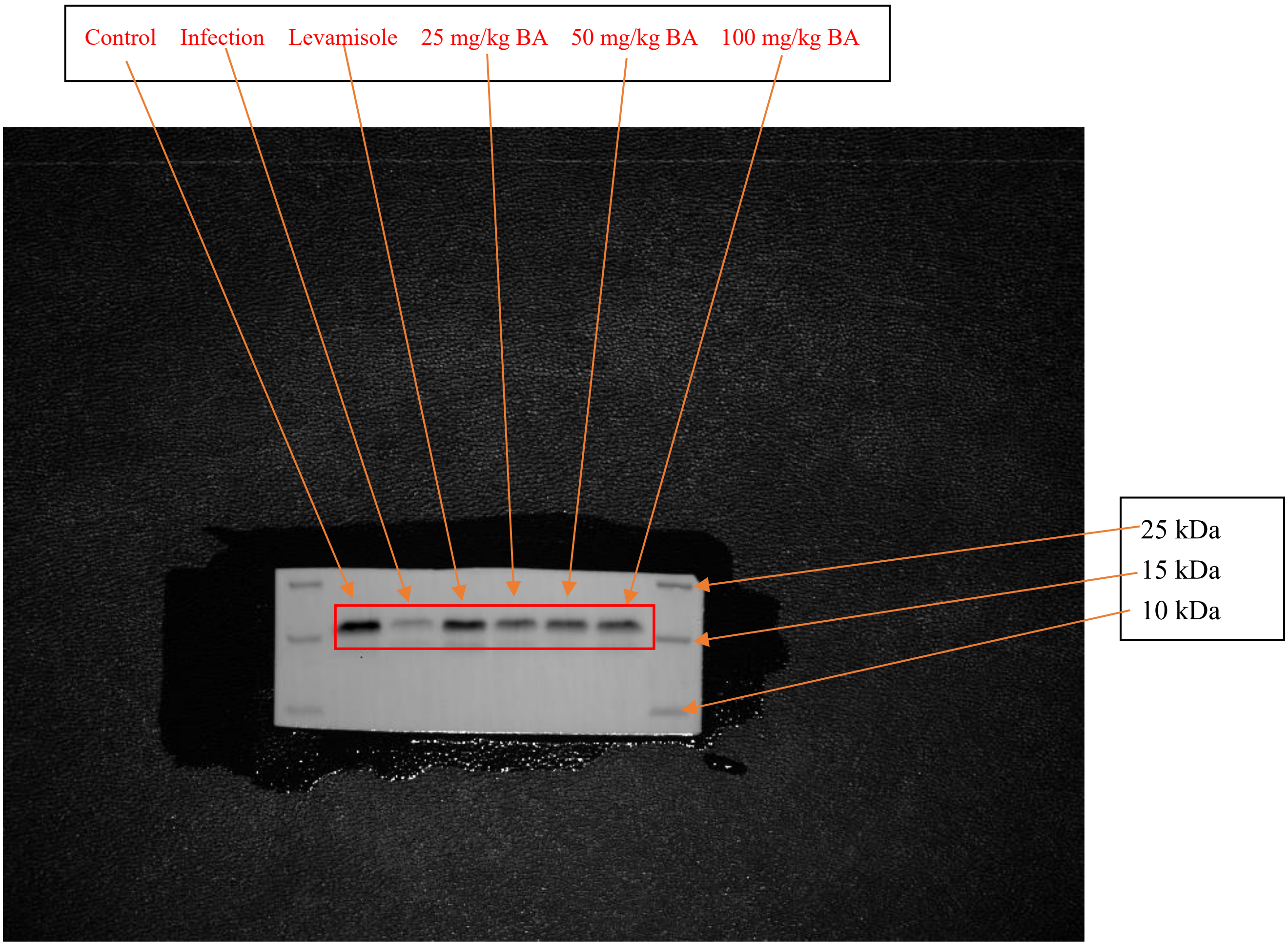

Fig 6

Fig 6. B:

LC3B (repeat 2)

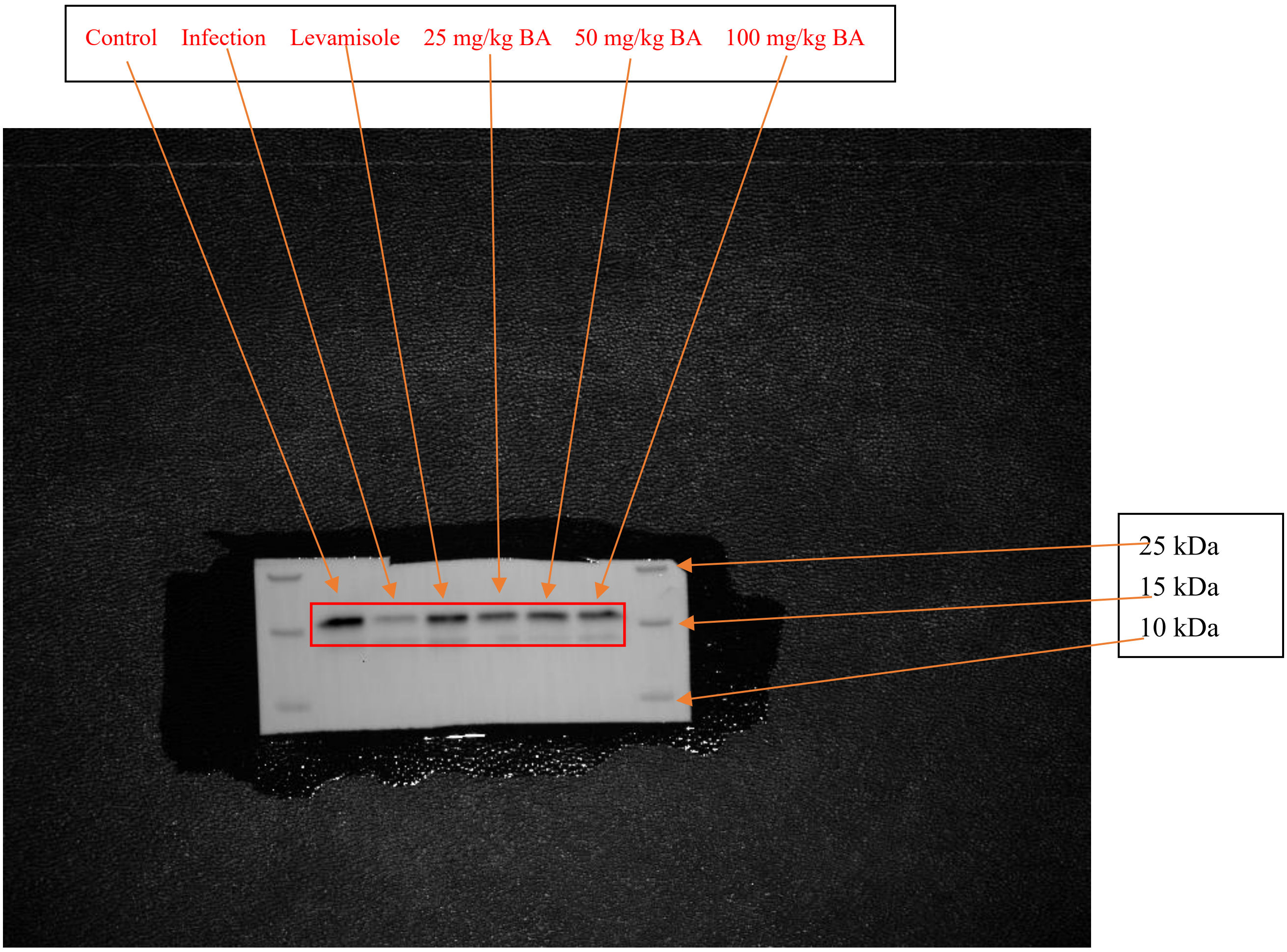

Fig 6

Fig 6. B:

LC3B (repeat 3)

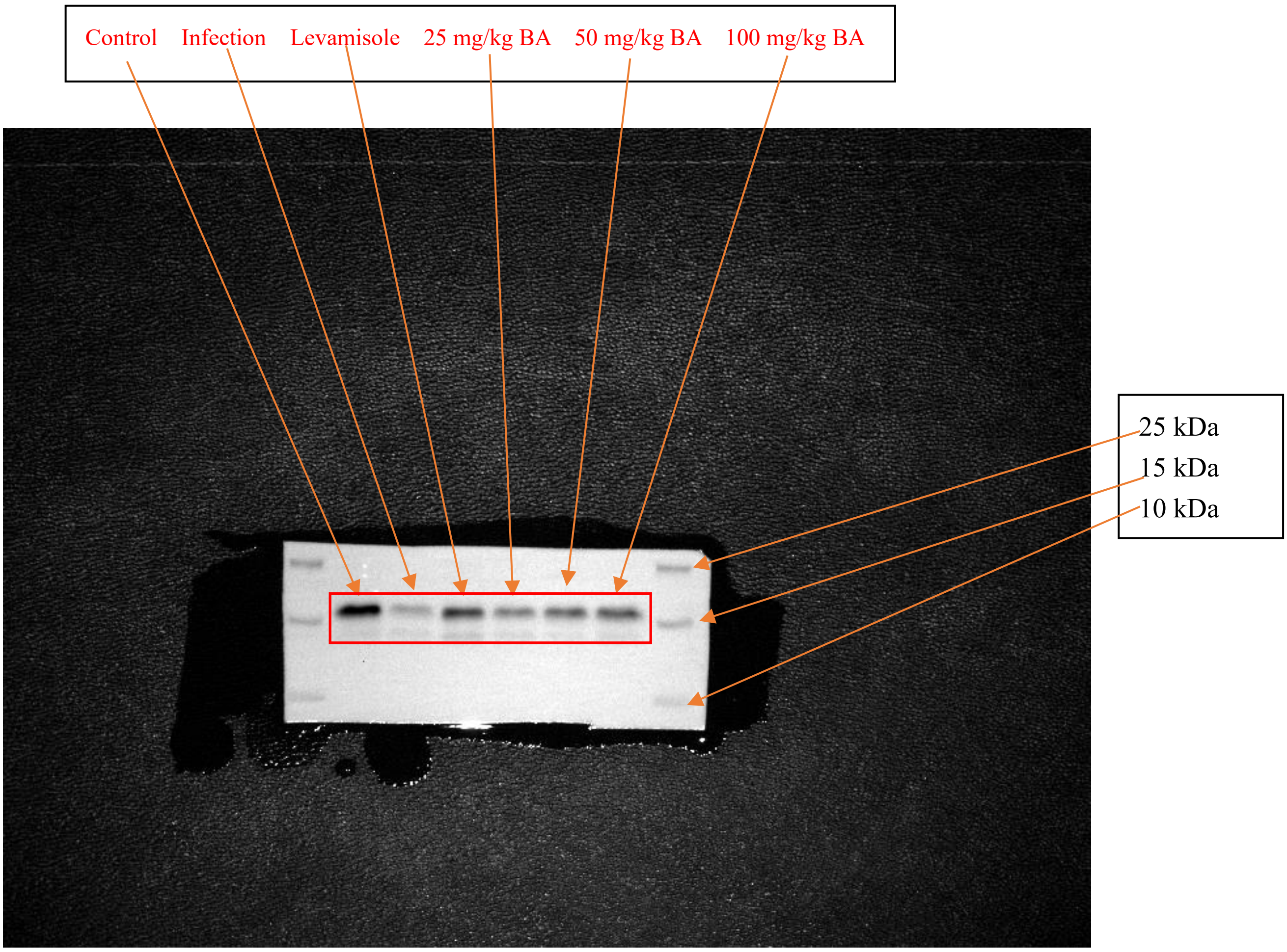

Fig 6

Fig 6. E:

GAPDH (repeat 1)

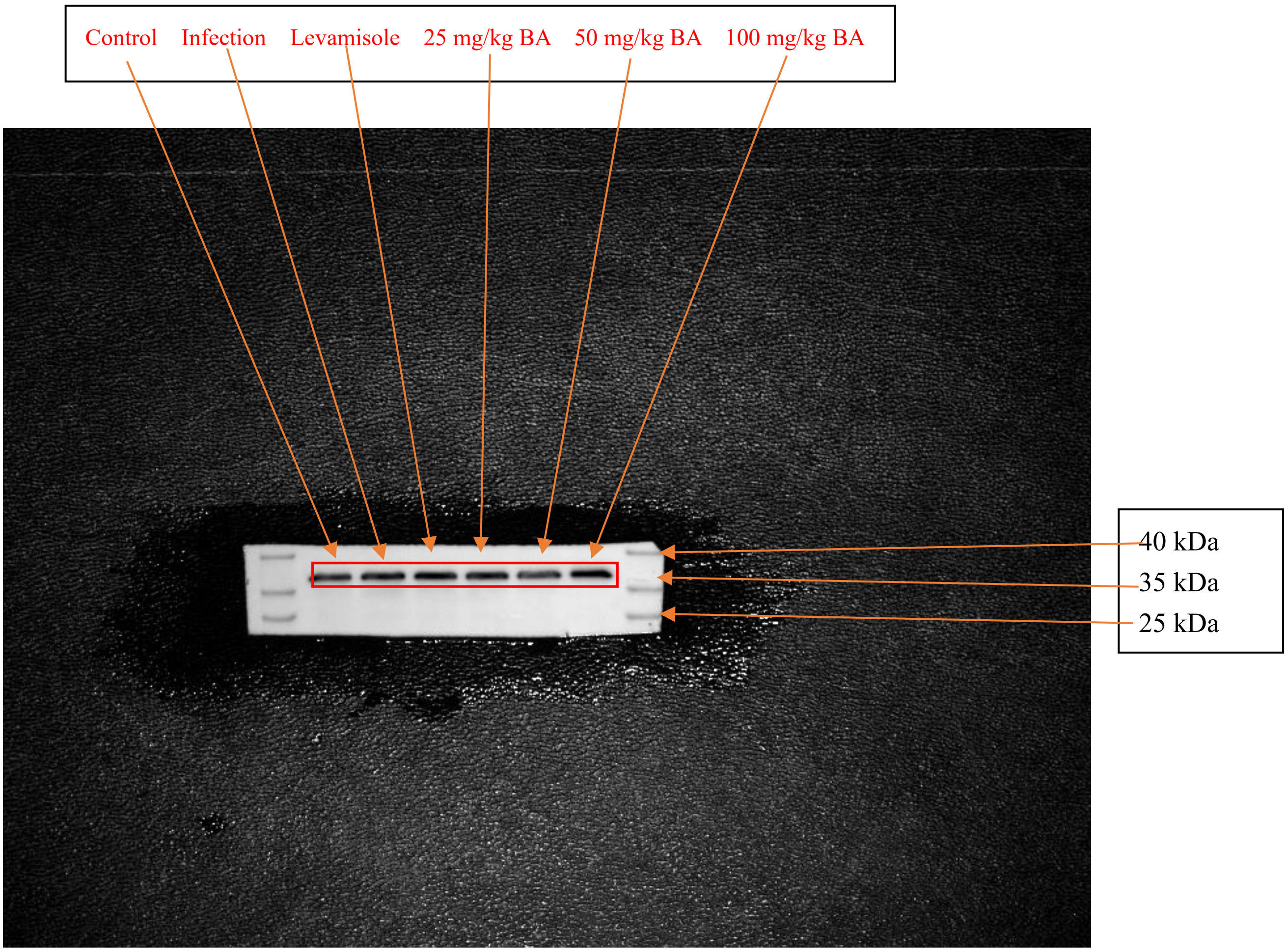

Fig 6

Fig 6. E:
GAPDH (repeat 2)

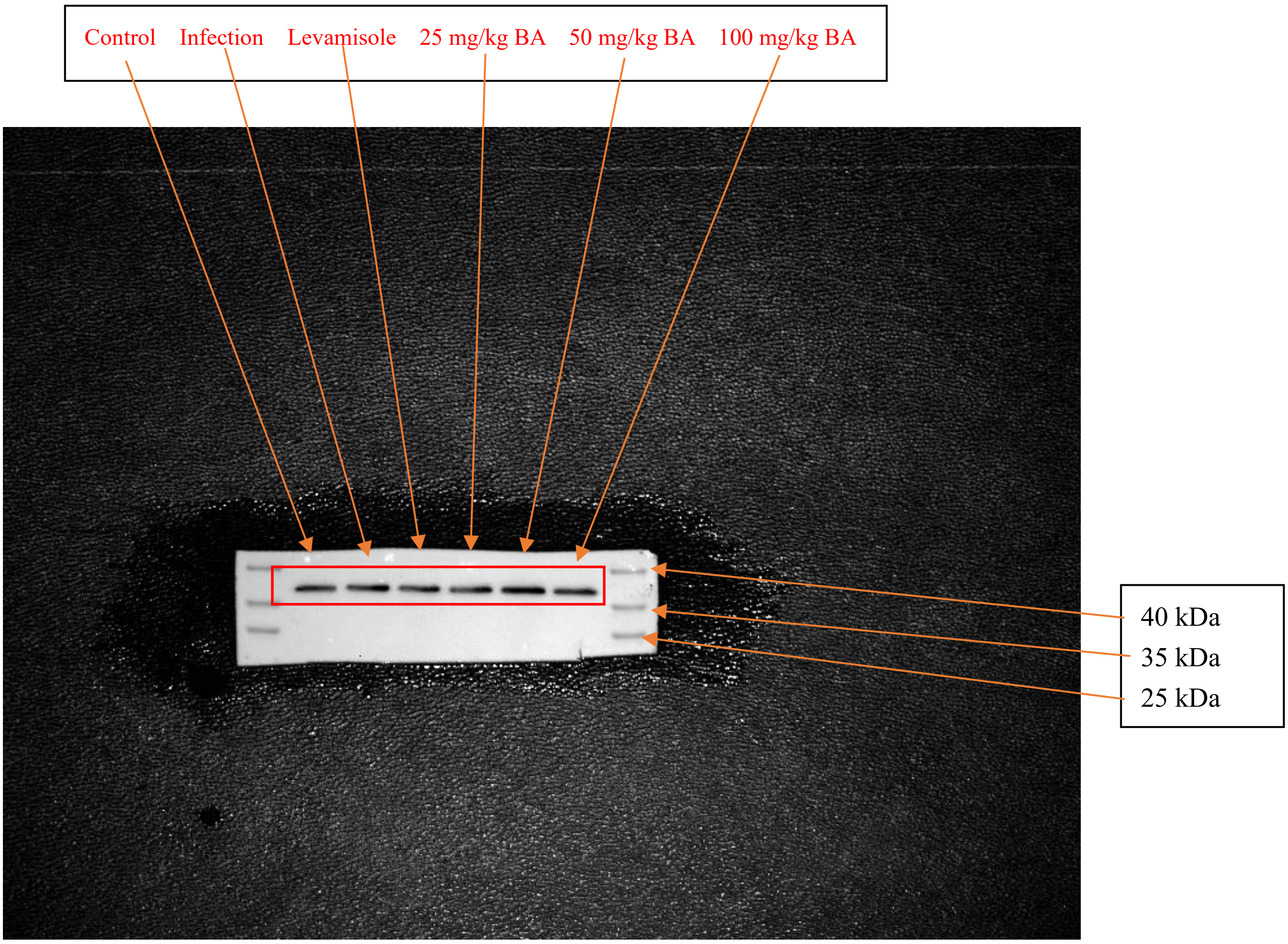

Fig 6

Fig 6. E:

GAPDH (repeat 3)

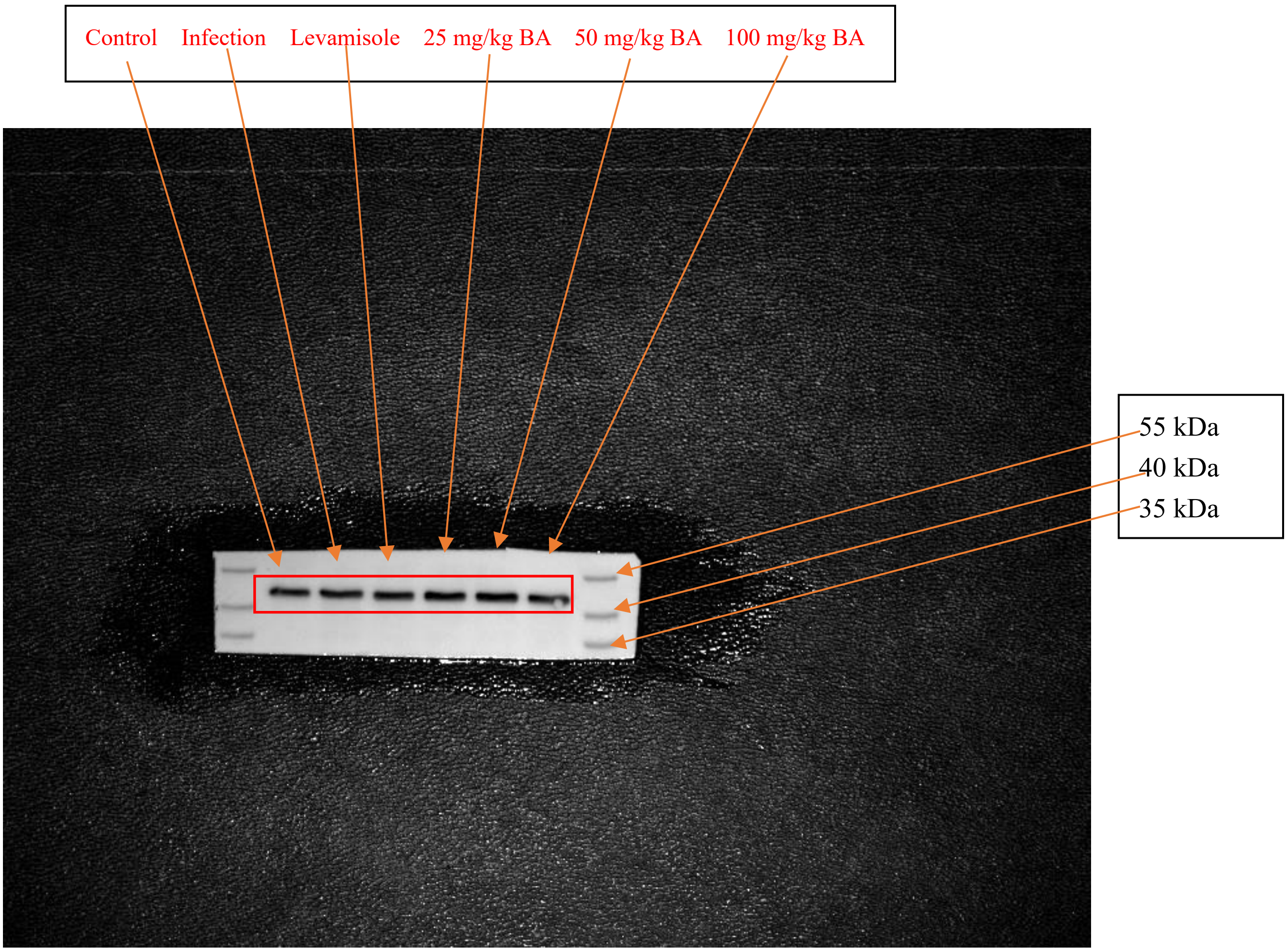

Fig 6

Fig 6. E:

P62 (repeat 1)

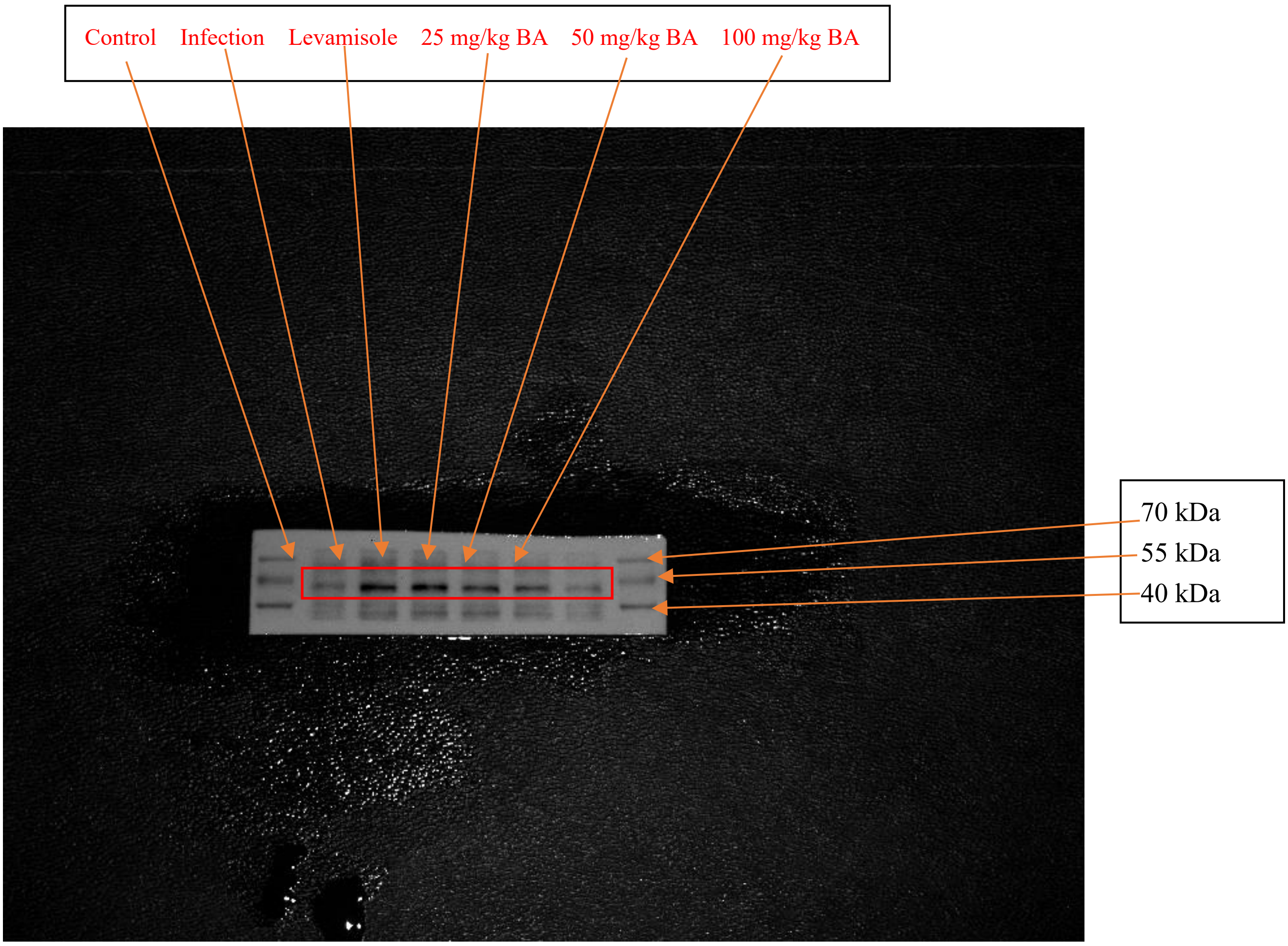

Fig 6

Fig 6. E:

P62 (repeat 2)

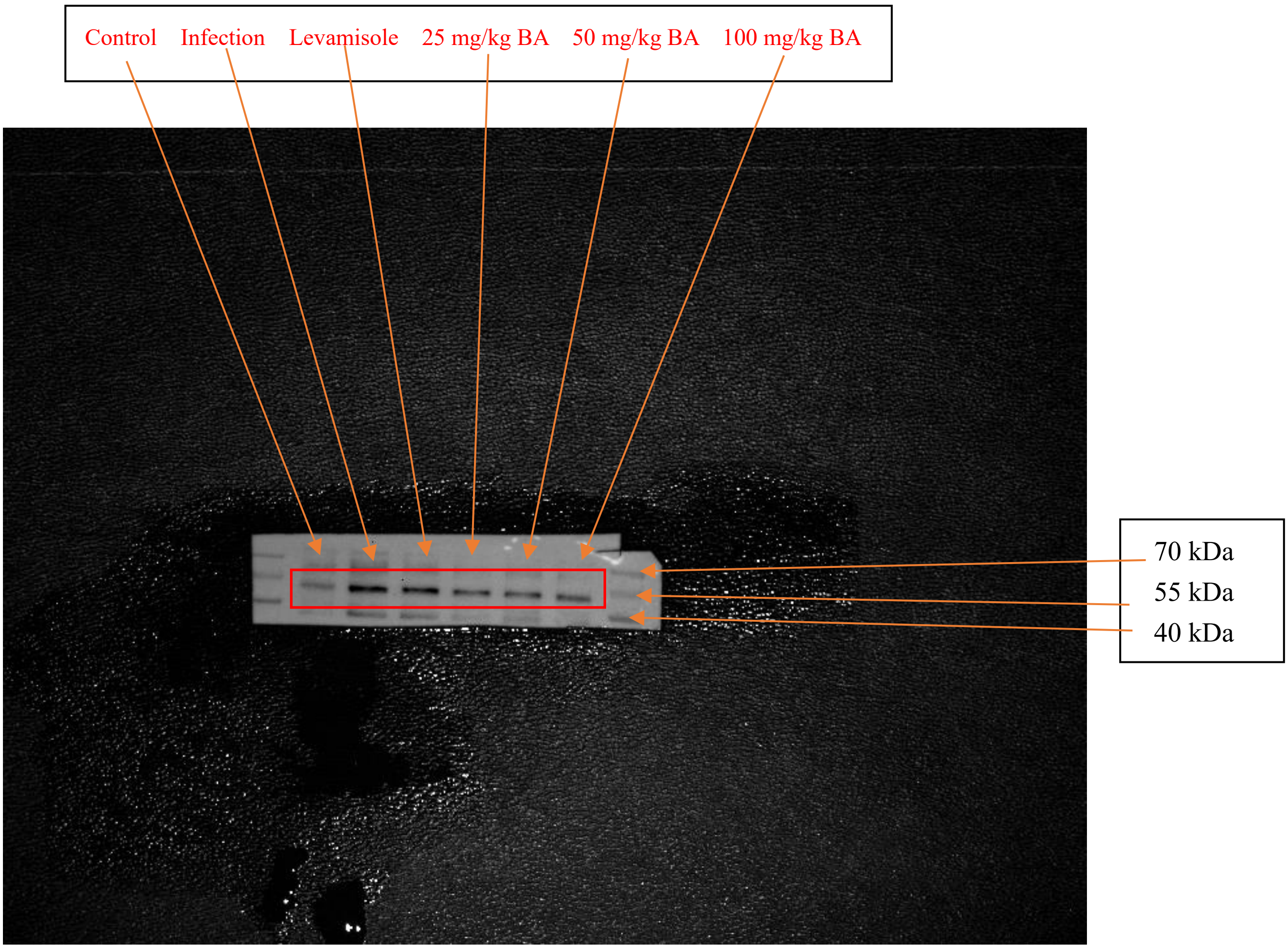

Fig 6

Fig 6. E:

P62 (repeat 3)

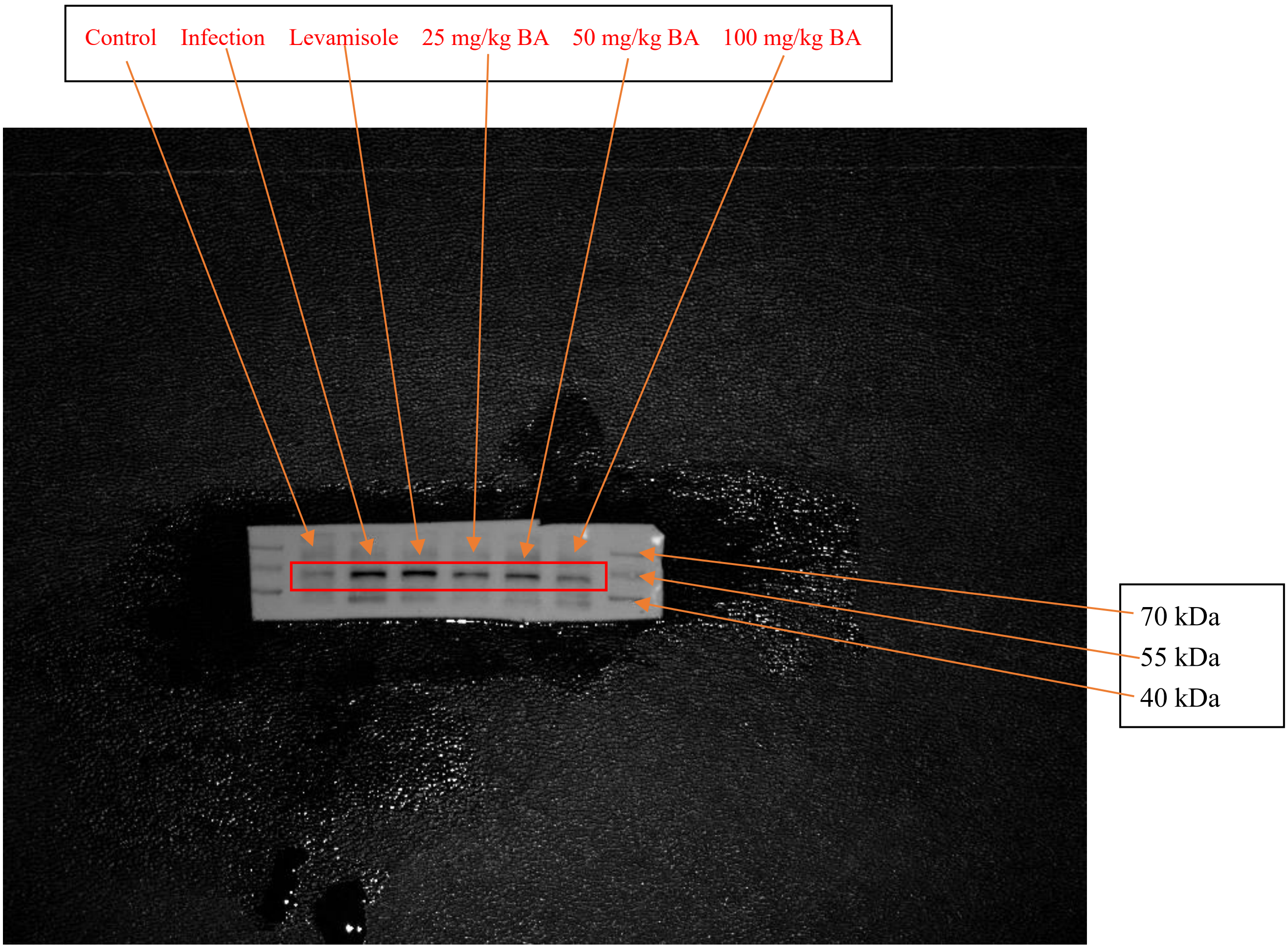

Fig 7

Fig 7. B:

GAPDH (repeat 1)

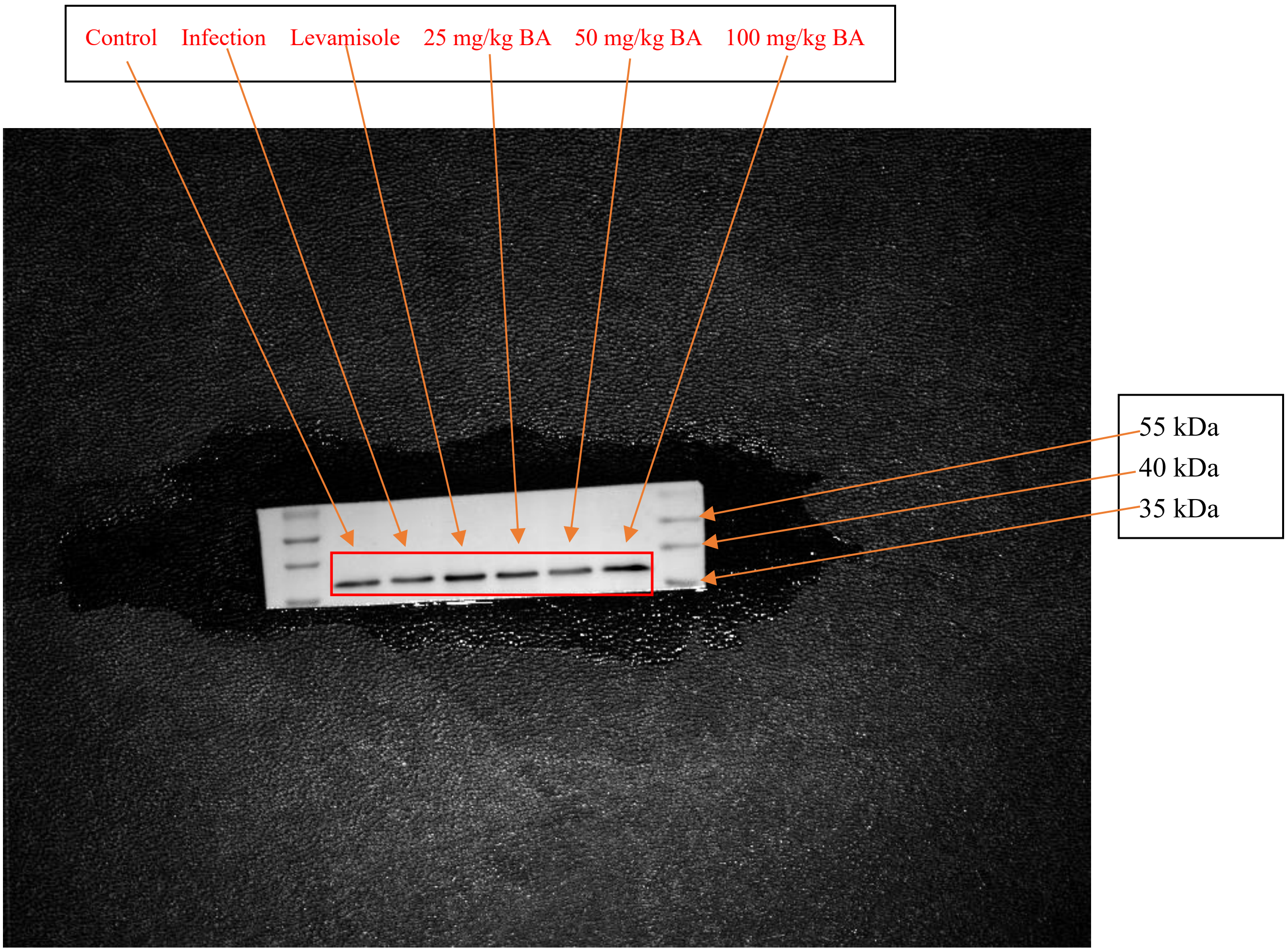

Fig 7

Fig 7. B:

GAPDH (repeat 2)

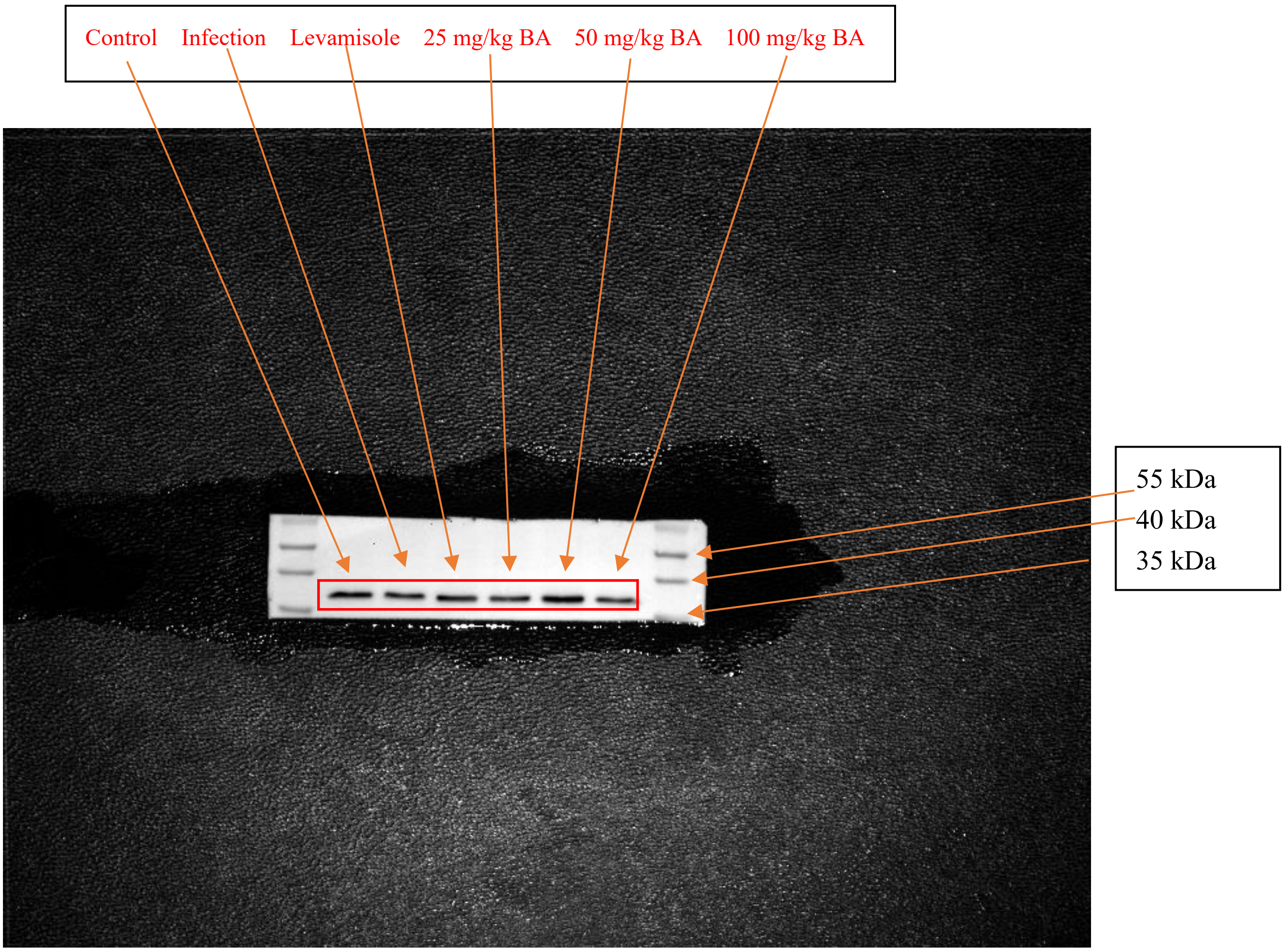

Fig 7

Fig 7. B:

GAPDH (repeat 3)

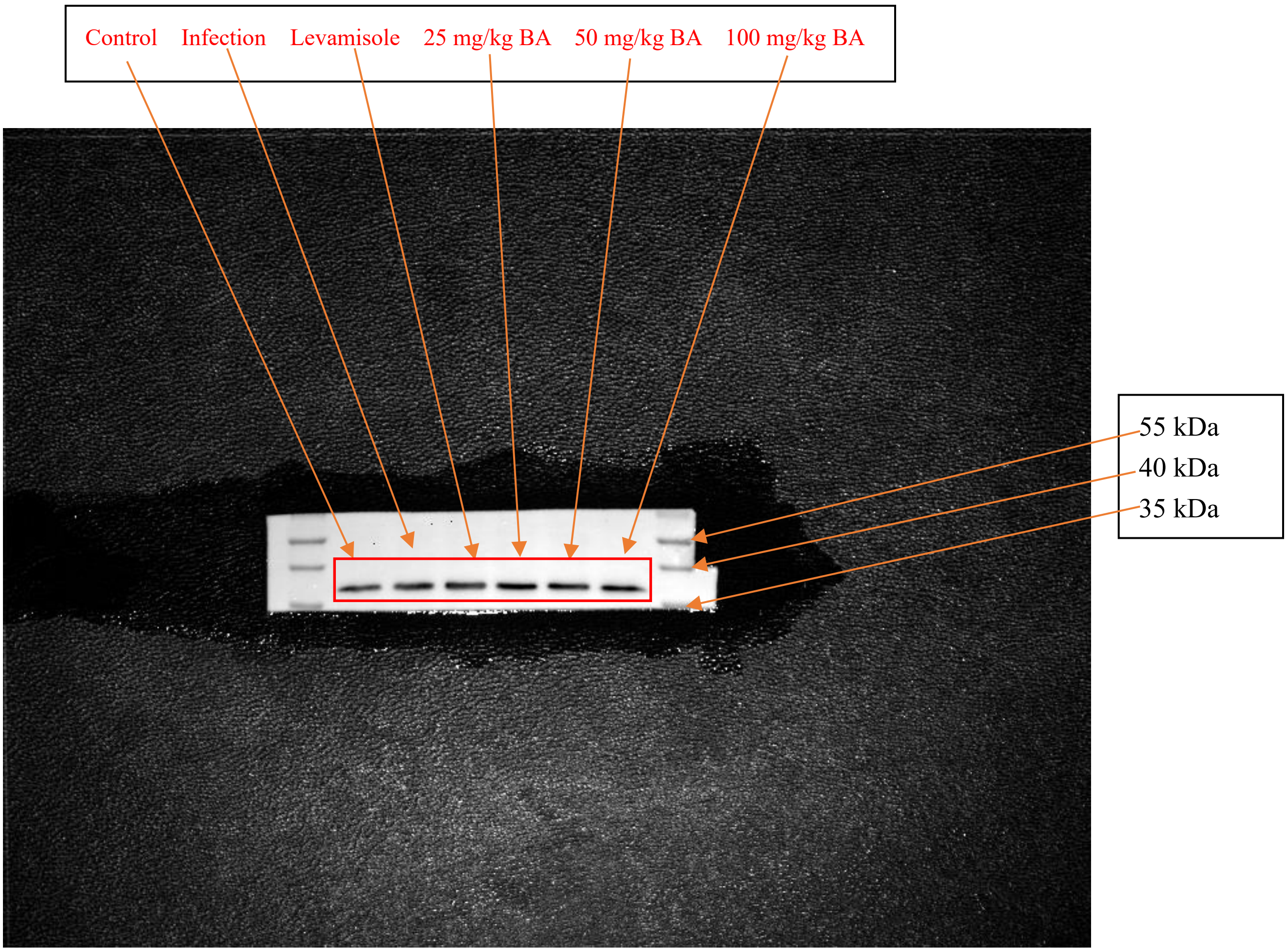

Fig 7

Fig 7. B:

NLRP3 (repeat 1)

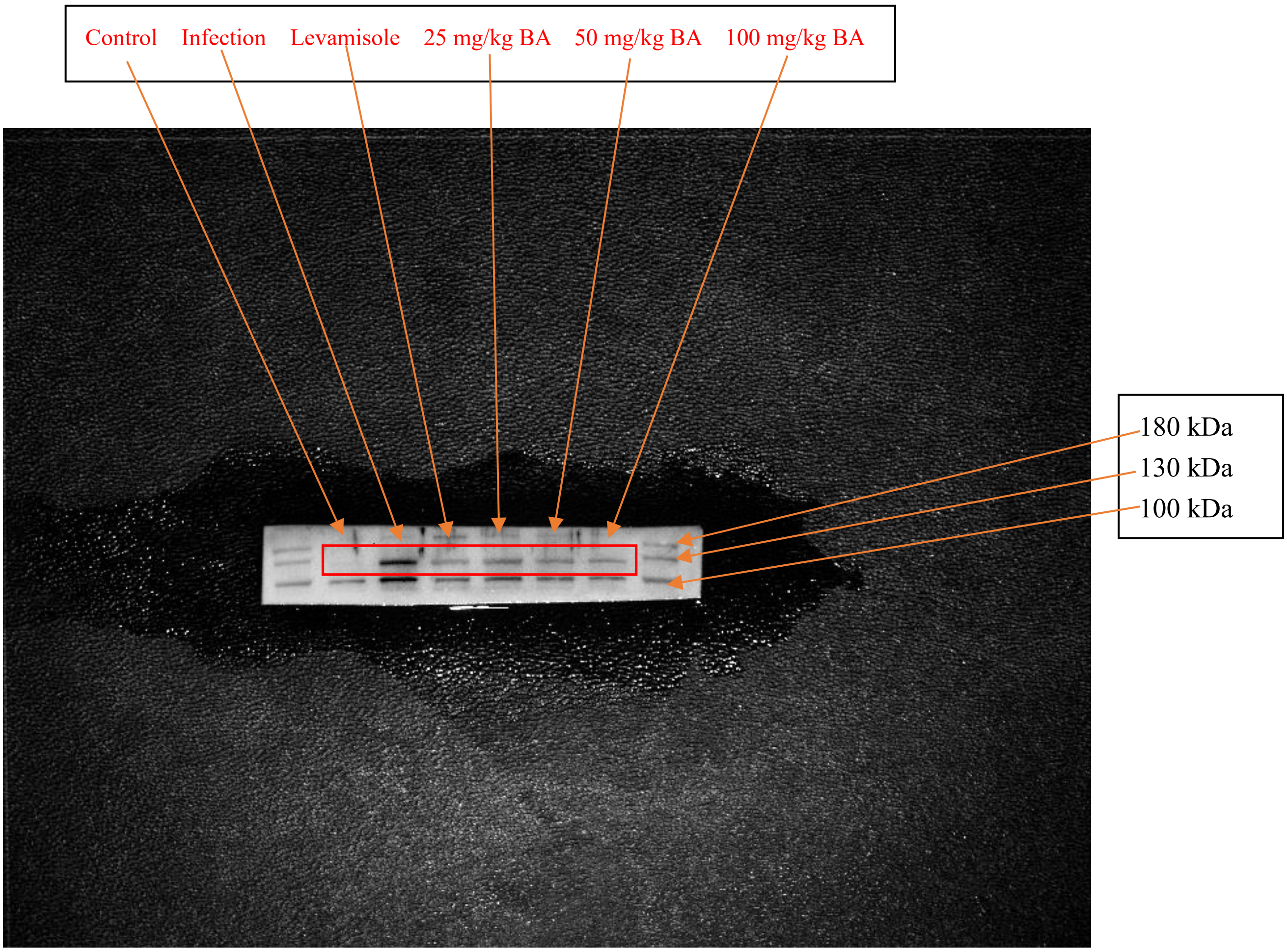

Fig 7

Fig 7. B:

NLRP3 (repeat 2)

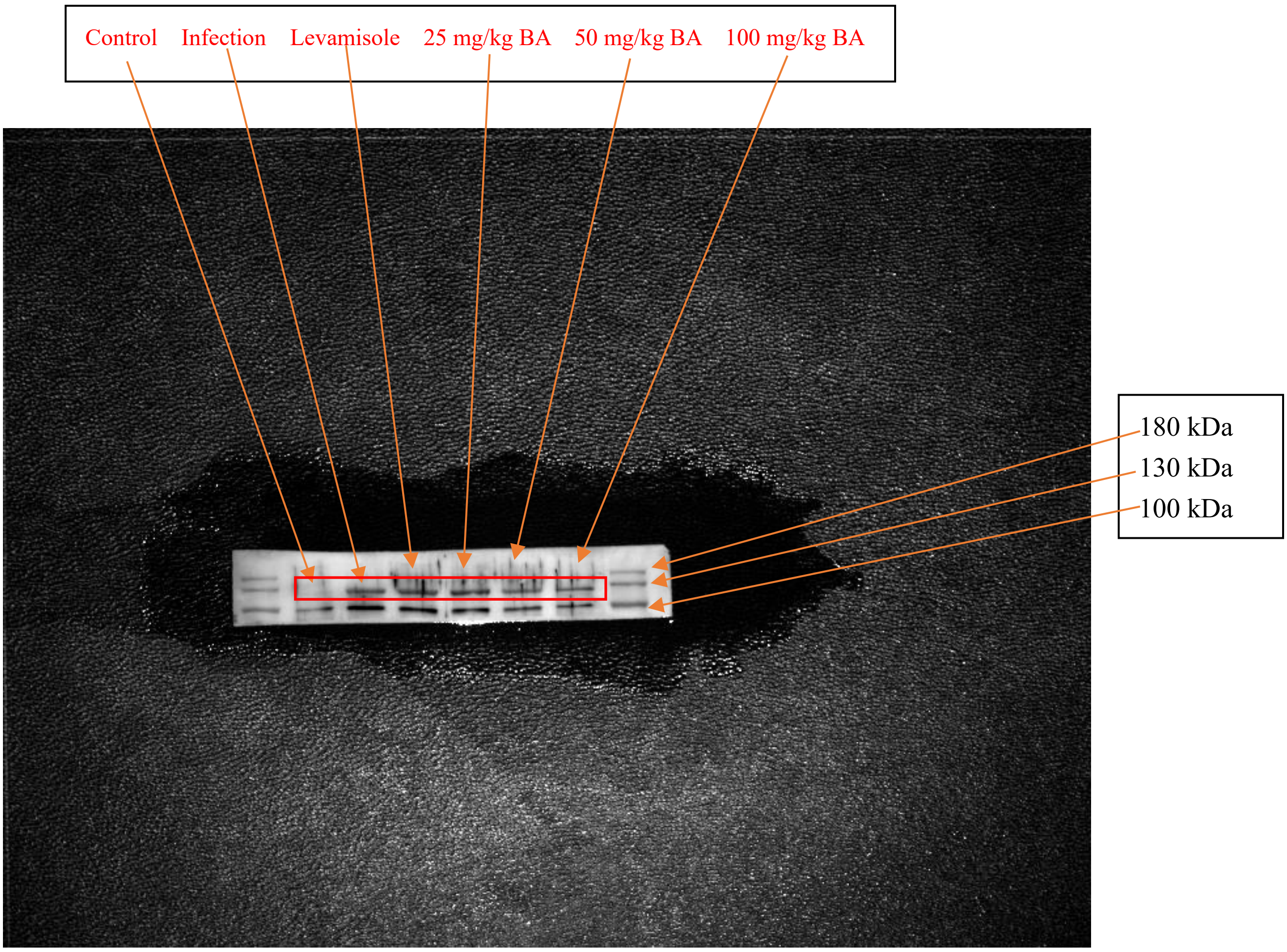

Fig 7

Fig 7. B:

NLRP3 (repeat 3)

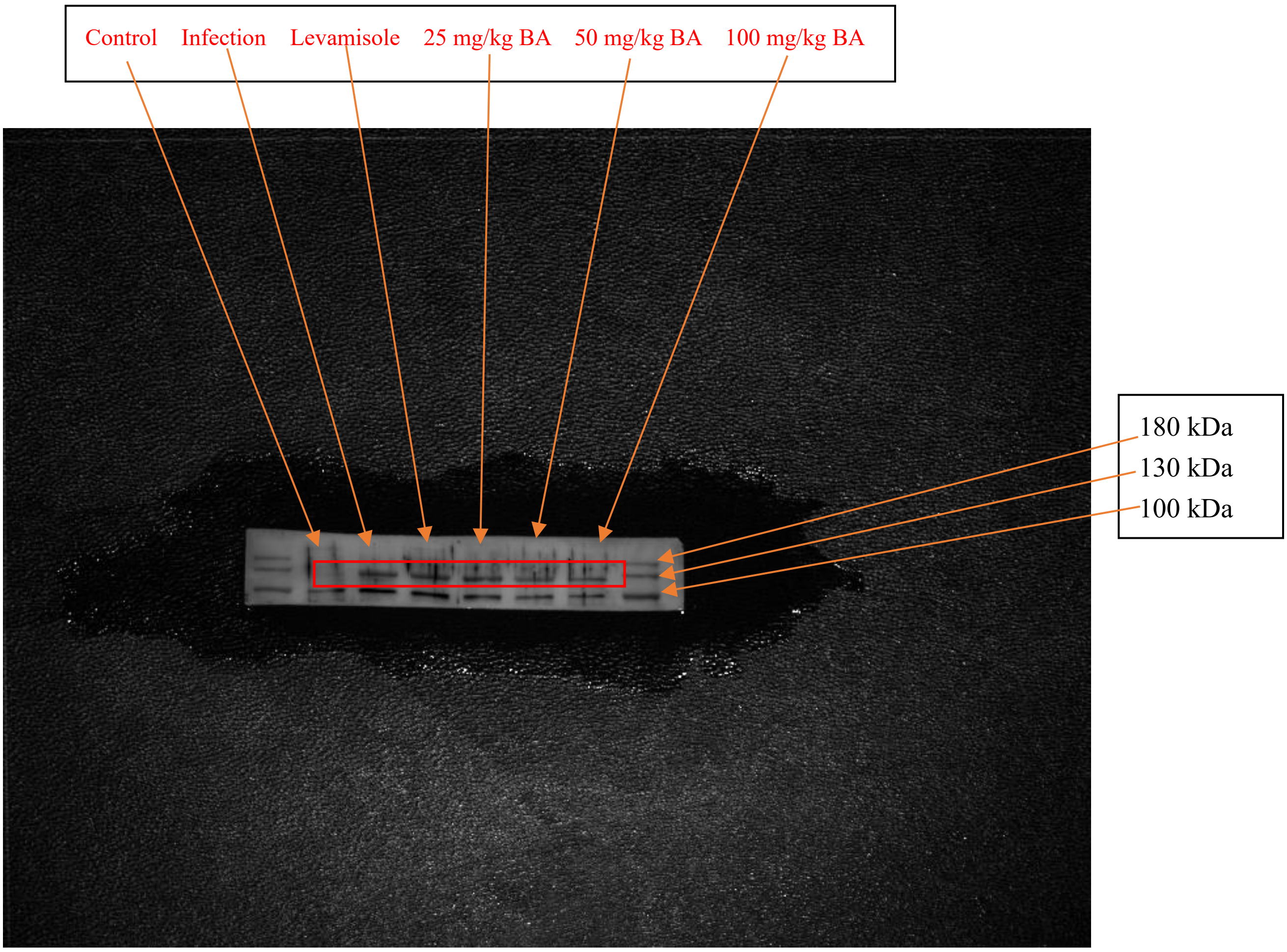

Fig 7

Fig 7. E:

GAPDH (repeat 1)

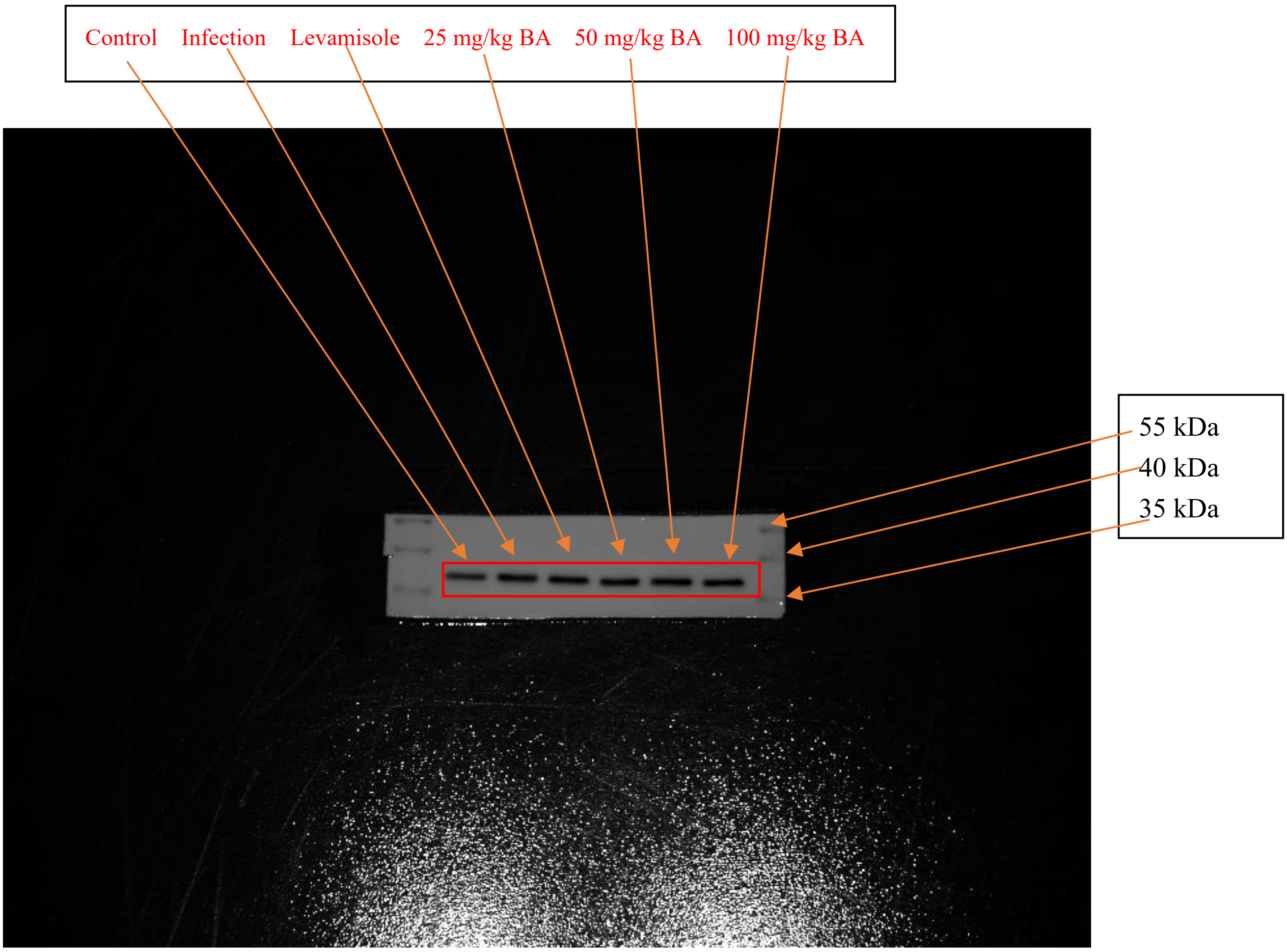

Fig 7

Fig 7. E:

GAPDH (repeat 2)

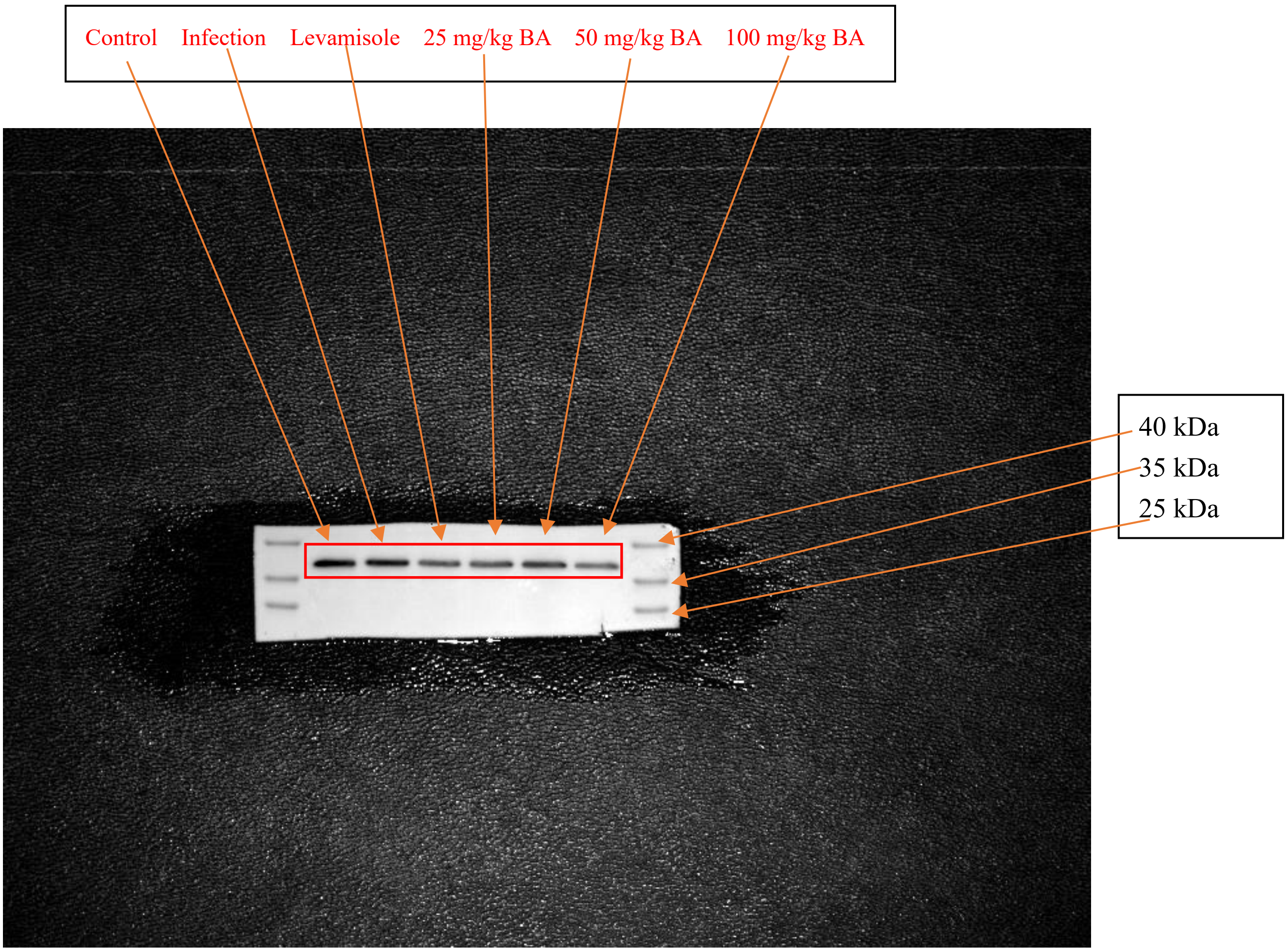

Fig 7

Fig 7. E:

GAPDH (repeat 3)

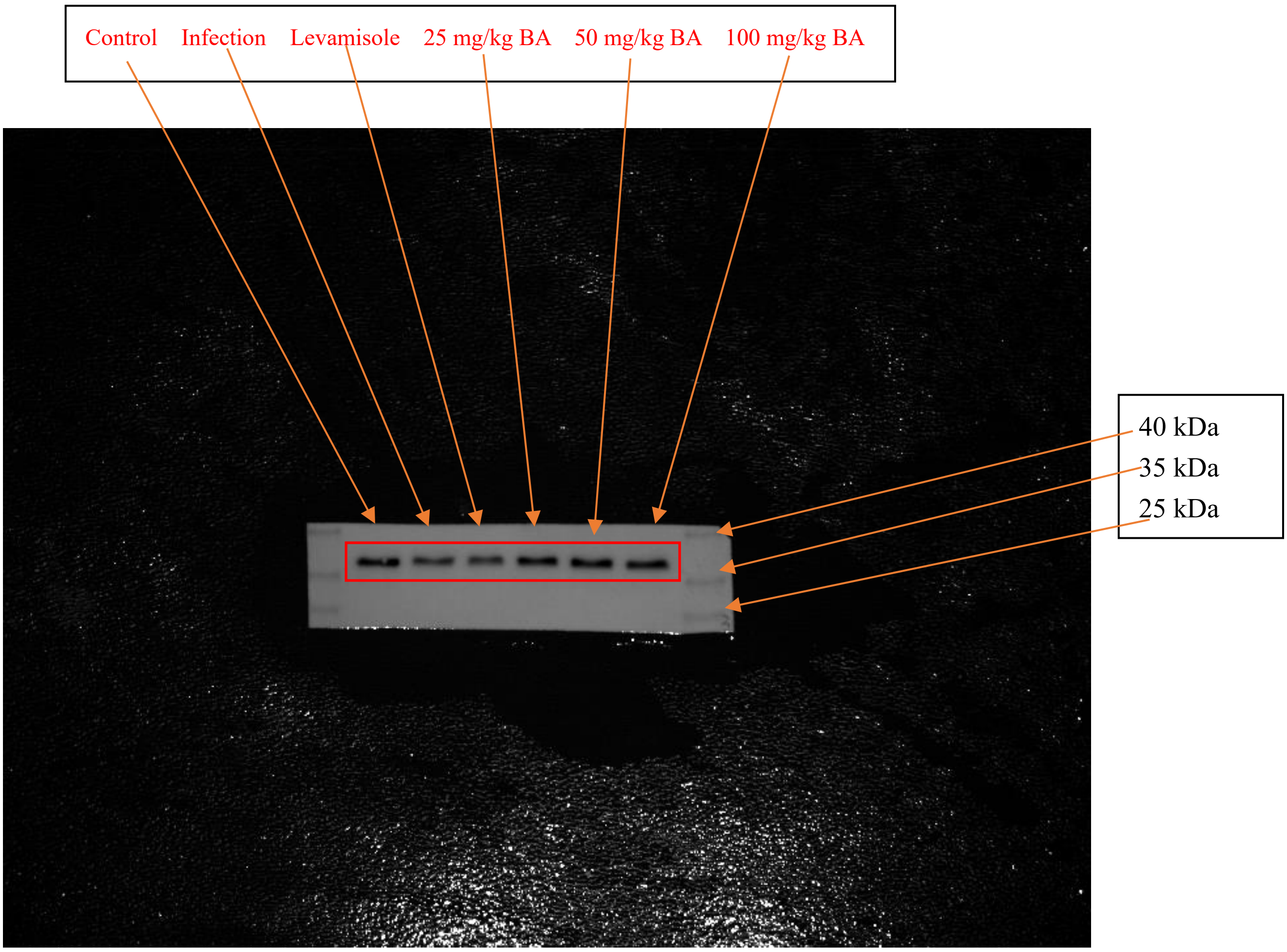

Fig 7

Fig 7. E:

Caspase1 (repeat 1)

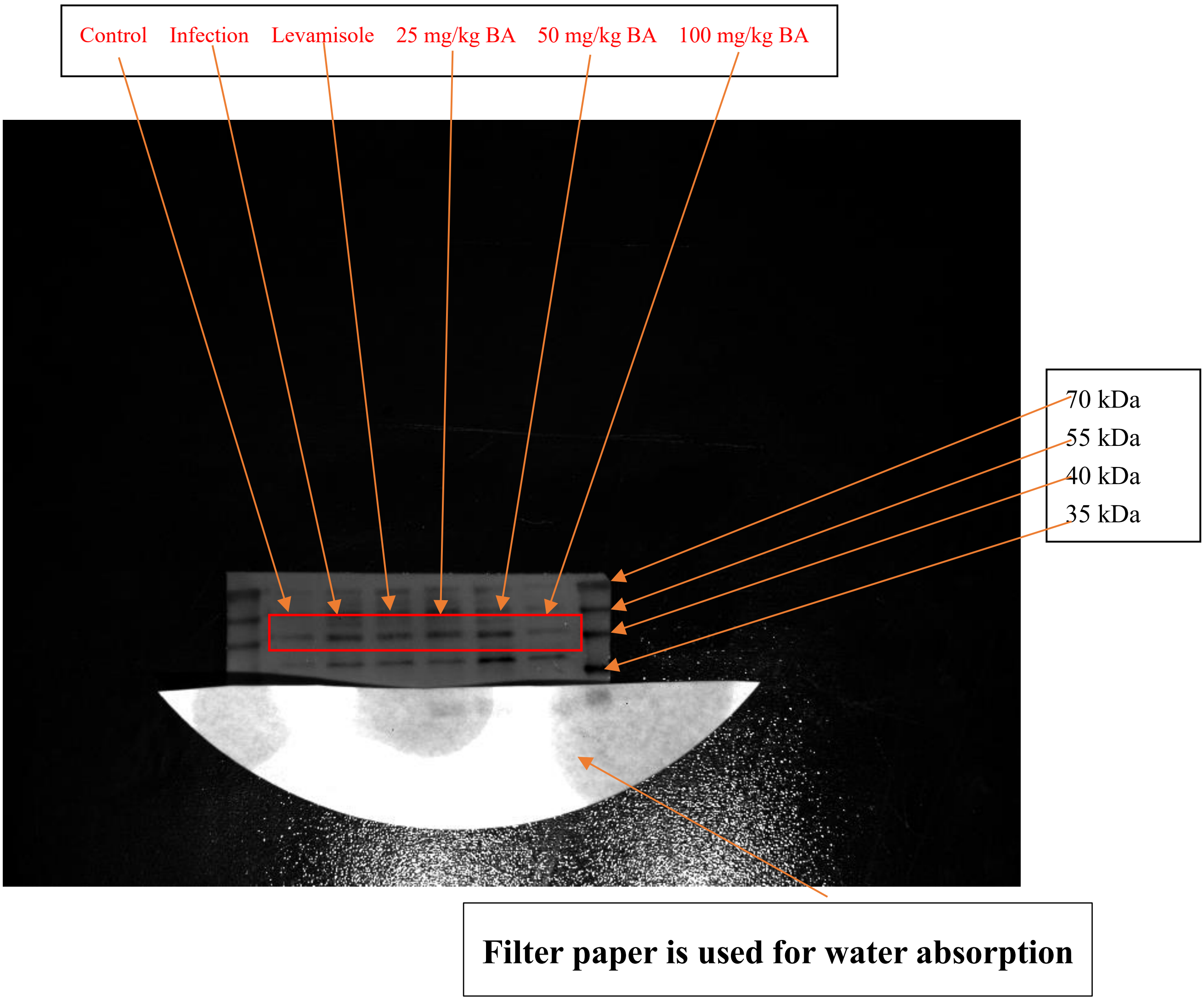

Fig 7

Fig 7. E:

Caspase1 (repeat 2)

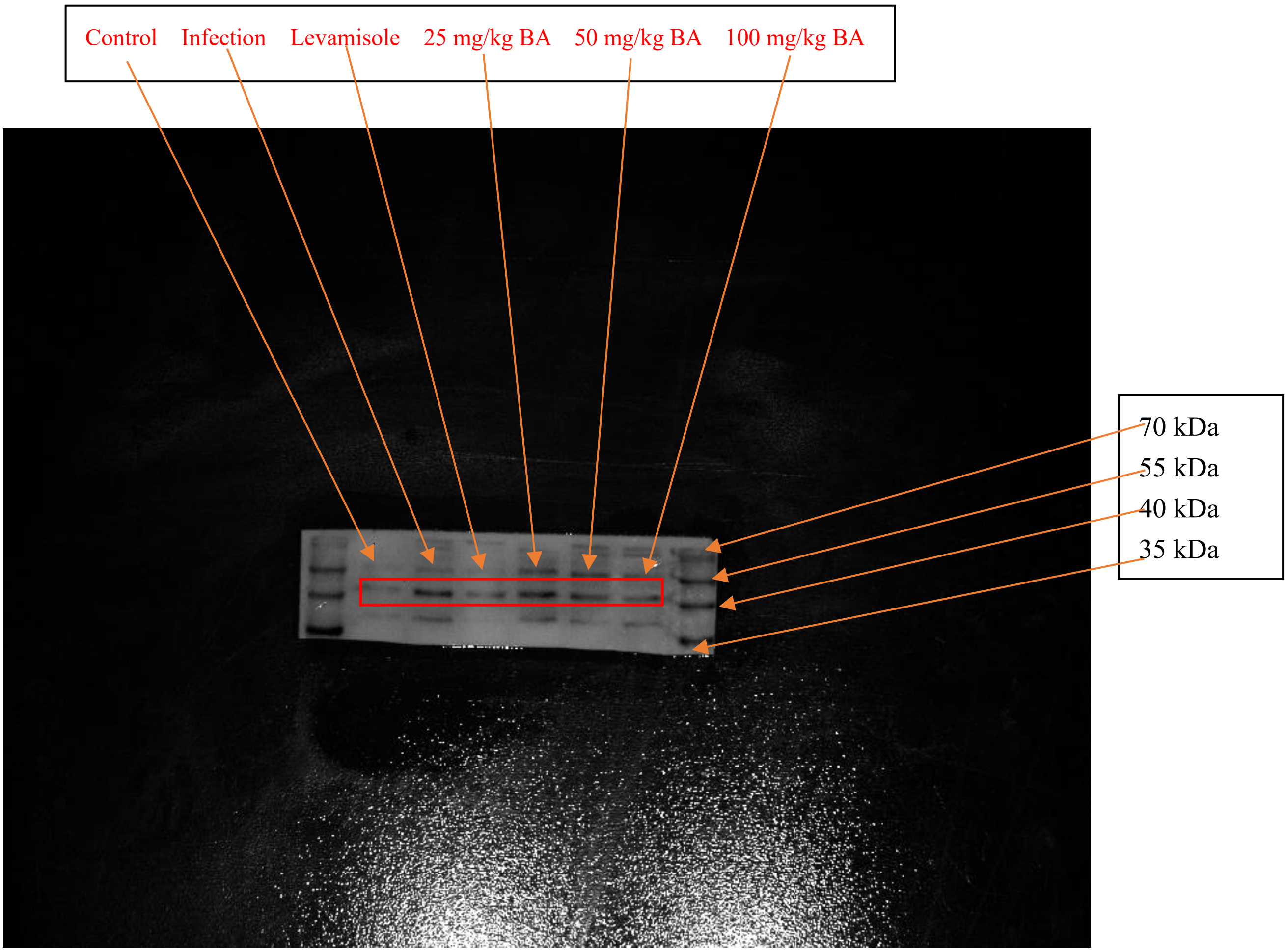

Fig 7

Fig 7. E:

Caspase1 (repeat 2)

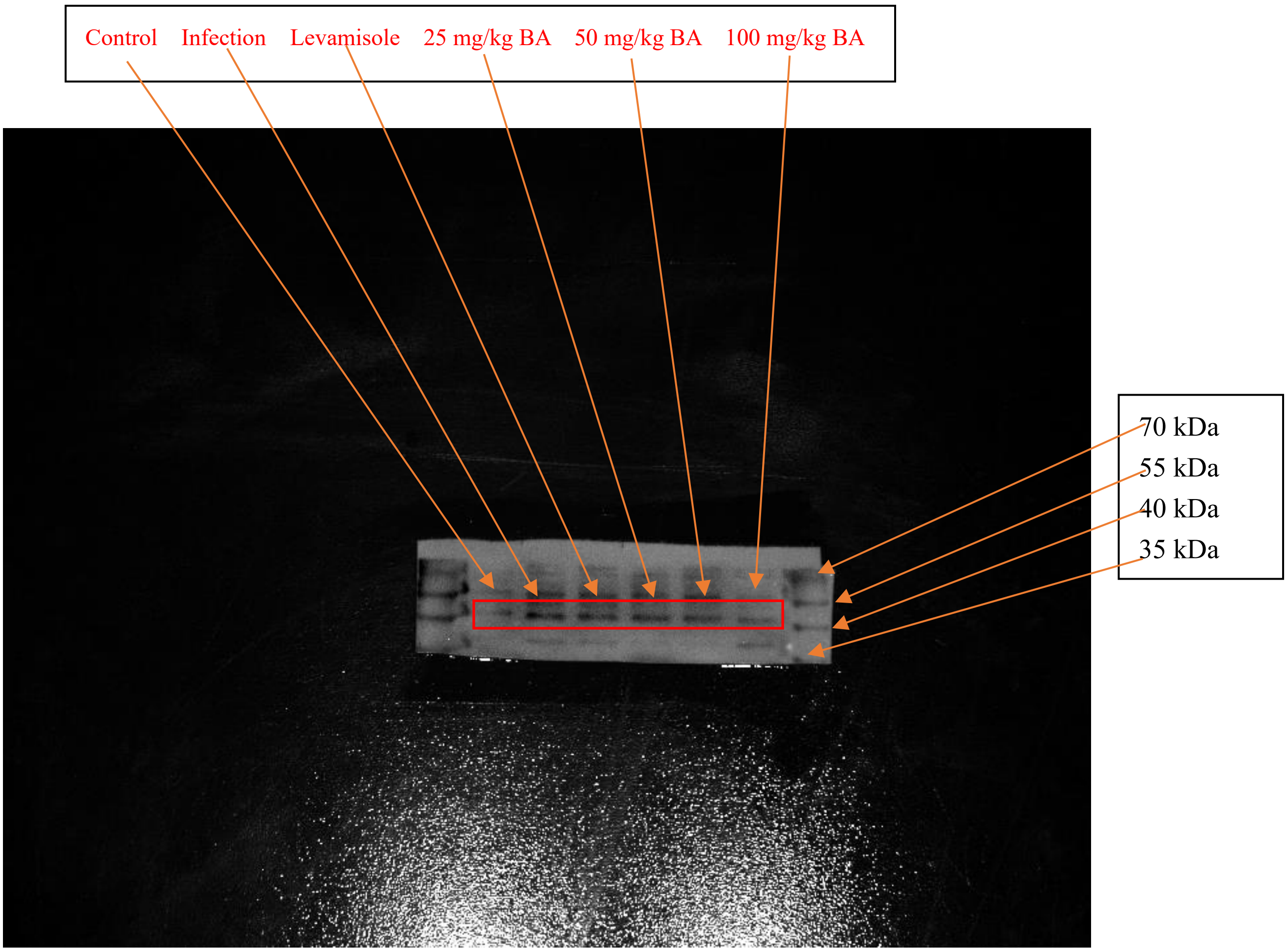

Supplement: Supplementary file 1 [file biomolecules-15-00722-s001.zip › biomolecules-3516576-supplementary.pdf]
